# Management of groundwater abstraction and seawater intrusion in the Moghra aquifer, Egypt

**DOI:** 10.1038/s41598-025-14432-y

**Published:** 2025-08-19

**Authors:** Asaad M. Armanuos, Martina Zeleňáková, Ahmed Shalby, Sobhy R. Emara, Abdelazeim Negm

**Affiliations:** 1 Irrigation and Hydraulics Engineering Department, Faculty of Engineering, Tanta University, Tanta, Egypt; 2 Institute of Environmental Engineering, Faculty of Civil Engineering, Technical University of Košice, 04200 Košice, Slovakia; 3 Department of Water and Water Structures Engineering, Faculty of Engineering, Zagazig University, Zagazig, Egypt

**Keywords:** Management, Groundwater, Seawater intrusion, Abstraction, Recharge, Civil engineering, Engineering

## Abstract

Groundwater is being utilized as a major freshwater resource in numerous nations across the world, particularly in arid and semi-arid countries like Egypt. The main objective of this study is to simulate the impact of groundwater abstraction and saltwater intrusion in the Moghra aquifer using MODFLOW and SEAWAT. Various abstraction rate scenarios from wells were modeled for a 100-year cultivation project period to study the impact of abstraction on aquifer drawdown. For 1000 wells, the maximum simulated drawdown equals 54, 66, 85, and 100 m for the abstraction rates of 1000, 1250, 1500, and 1750 m^3^/day/well, which represents about 18%, 22%, 28%, and 30% of the saturated Moghra aquifer thickness, respectively. The consequence of increasing the abstraction rate is substantial on seawater intrusion in the Moghra aquifer. The concentration line of 3500 mg/L intruded inland into the Moghra aquifer to a distance of 30.7, 52.7, and 57.1 km in the eastern, central, and western parts, respectively. Increasing the rate of abstraction from 1000 to 1750 m^3^/day/well led to the advance of the seawater (3500 mg/L) inland to the Moghra aquifer by a distance of 0.6, 5.6, and 4.4 km in the eastern, central, and western parts, respectively. Between the proposed controlling methods, artificial recharge, in conjunction with saline water abstraction, has a significant impact on attenuating the seawater back to the seaside compared with each method alone. The outcomes of this study can be used for developing groundwater resources in both arid and semi-arid areas in a sustainable manner.

**Supplementary Information:**

The online version contains supplementary material available at 10.1038/s41598-025-14432-y.

## Introduction

Groundwater is the primary source of freshwater in the world, representing approximately 33% of freshwater abstraction ^1^. Chakrabarti ^2^ estimates that over 2.5 billion people globally depend on groundwater to meet their daily requirements. Arid and semi-arid areas experience this state due to limited rainfall and surface water availability. The main factors that alter groundwater storage and aquifers’ dynamic state are extensive exploitation and climate change ^3^. Excessive pumping of groundwater reserves, particularly in low and nonrenewable aquifers, leads to significant decreases in groundwater levels and consequently deterioration of water quality ^4^. In many nations throughout the world, particularly in dry and semi-arid areas like Egypt, groundwater has emerged as a vital freshwater supply ^5^. Due to significant population growth and shortages of surface water, the Egyptian government is looking for other water supplies like groundwater and water from desalinating seawater. Following the completion of the Grand Ethiopian Renaissance Dam, which decreased Egypt’s quota of the Nile River, the Egyptian government declared plans to boost development within the western desert to relieve the stress placed on available resources of surface water ^6,7^. The Moghra aquifer provides the water needed for the population of Moghra, a recently constructed neighborhood. This reservoir of water needs to be well maintained to guarantee that there is enough water in it for continued growth, as it is regarded as a vital aquifer in the Western Desert’s northern region. According to Mansour et al. ^8^, there are numerous potential causes of groundwater contamination, but saltwater intrusion (SWI) is particularly frequent. SWI is a natural occurrence that causes seawater to displace the aquifer’s freshwater ^9,10^. SWI is divided into active and passive categories by Badaruddin et al. ^9^. Whether the freshwater flows toward the sea or the land depends on that direction.

Ezzat ^11^ conducted a hydrogeological study to anticipate the impacts of the projected Qattara saltwater lake on the research area’s groundwater, notably the sandstone Moghra aquifer, which was hydrodynamically balanced. A regional numerical model was developed for the Moghra aquifer, which extends from Wadi El Natrun to the Qattara Depression and continues north to the Mediterranean Sea. The study gave an overview of the hydrogeology, geology, and geomorphology of the aquifer groundwater system in the study region. The study contained hydraulic characteristics, contouring maps for the Moghra aquifer’s upper and lower limits, and an explanation of groundwater movement based on data acquired at the time. Rizk and Davis ^12^ developed a two-dimensional model using geology and hydrogeological data, as well as some field data, to represent the Moghra aquifer in the Western Desert area and the groundwater status at the time. The study intended to forecast groundwater levels upon applying the model in both steady-state and unsteady circumstances. The investigation demonstrated that the computed heads by the model matched the field data. This ensures that the Qattara Depression serves as a sink for groundwater in the region. Furthermore, the steady-state water levels in the Qattara reservoir are projected to increase due to the Mediterranean Sea surface water inflow of 500 m^3^/s, as opposed to the unstable state run, which was unable to do this. El Sabri et al. ^13^ created a mathematical model for the Moghra oasis and tested multiple scenarios to determine the ideal operation scenario for maintaining the aquifer for 100 years. The study focused on hydrochemical facies, which were revealed through examining the collected water samples.

Groundwater quality is influenced by the interactions of soil formation and water; therefore, analysis is required to comprehend the variation in the quality of groundwater ^13^. As stated by RIGW ^14^, the salinity of the water varies between 1500 to 8000 mg/L based on the chemical analysis of groundwater samples obtained from wells dispersed throughout the Moghra area. Furthermore, salinity reduces near the source of fresh water supply to the east (coming across the western delta), whereas it gradually rises to the west and northwest, to around 8,500 mg/L, due to the impact of saltwater invasion from the north. In accordance with El Sabri et al. ^13^, the aquifer’s Iso salinity map indicates that salinity rises in the north and northwest. Furthermore, it flows in the same direction as the groundwater. This investigation was conducted in a tiny domain of the Moghra oasis. The salinity levels in the oasis differ significantly depending on where the wells are located. This variation in salinity levels is additionally linked to the lithologic features present in the formation that contains water. Given that the Moghra aquifer’s groundwater contains higher concentrations of TDS, B, Fe, and Mn than the highest limits permitted and advised by the WHO ^15^ – 1,000 mg/L for TDS, 0.5 mg/L for B, 0.3 mg/L for Fe, and 0.4 mg/L for Mn – El-Sayed and Morsy ^16^ deemed both the groundwater’s chemical and physical quality unfit for human consumption. Furthermore, the Moghra aquifer’s groundwater was categorized as extremely hard water in accordance with Durfor and Becker’s ^17^ hardness rating, making it unfit for use in residential settings. However, they additionally emphasized that, under certain circumstances, such water may be utilized for irrigation. Emara et al. ^18^ conducted a GIS-based vulnerability assessment using the GALDIT index, enhanced by hydrochemical analyses (HFE diagram and GQISWI). The findings revealed that most of the area is moderately to highly vulnerable to SWI. The modified GALDIT model provided improved spatial risk mapping, supported by groundwater chemistry data indicating widespread salinization. The study underscores the need for sustainable groundwater management to prevent further degradation.

Shalby et al., ^19^ used the FEFLOW model to construct a model in three dimensions for the Moghra aquifer. The observed water levels were used for model calibration. GRACE-based storage of groundwater was implemented in the process of tuning the established model. Eight various groundwater abstraction scenarios were checked from 1000 groundwater wells, with abstraction rates ranging between 800 to 1500 m^3^/day/well for 100 years for project cultivation. The outcomes confirmed that the maximum expected drawdown varied between 59 to 112 m and represented about 20 to 40% of the Moghra aquifer saturated thickness. The suggested development plan, which aims to keep the consequent drawdown below one meter annually, is in compliance with the withdrawal rationing law. Gomaa et al., ^20^ utilized the SEAWAT code of the GMS for simulating the groundwater flow and salt transport in the Moghra groundwater aquifer. The outcomes confirmed that the groundwater flow and groundwater quality were affected by the groundwater abstraction from wells. The salinity concentrations are predicted to increase based on the position of wells.

Selim et al. ^4^ evaluated the sustainability of using groundwater from the nonrenewable Moghra aquifer to support a rural development plan for reclaiming 135,000 acres. A combined groundwater flow and salinity transport model was created and validated using recent data. Simulations over 32 years under the original plan (570 wells) showed a significant drawdown of 26.68 m and inland saltwater intrusion of 32.94 km. Under a worst-case climate change scenario, these impacts worsened to a 31.16 m drawdown and 35.21 km intrusion. The findings indicated that full implementation of the plan could lead to aquifer depletion and major salinization. To ensure long-term viability, the study recommends reducing the cultivated area by 50%.

Given that the main source of water in coastal areas is groundwater, the development of those areas is mostly dependent on it. A major environmental issue that reduces groundwater quality is the invasion of saltwater into freshwater coastal aquifers ^21^. Several suggestions have been made for reducing or eliminating SWI in coastal aquifer systems ^22–24^. Abstraction of saltwater along the coastline (Pumping or negative barriers) ^25,26^, moving abstraction wells, using physical surface or underground barriers ^18,27,28^, artificial or natural replenishment (pressure barriers or positive barriers) ^27,29–31^, and combining methods (mixed barriers) ^30,32–35^ are some of these controlling approaches ^36^. To maintain a system’s coastal gradient, it should be controlled by increasing the inland groundwater heads; artificial replenishment is used for coastal aquifers with high-quality water (such as surface water, precipitation, abstracted underground water, recycled water, or desalinated water), primarily inside positive or pressure barriers.

Previous research in the Moghra aquifer focused on investigating the impact of groundwater abstraction from wells according to different scenarios to fulfill the agricultural development on the area. Only one published research simulated the seawater intrusion in the Moghra aquifer, but it did not configure the advancement of salinity in the Moghra aquifer with respect to the proposed groundwater abstraction and did not consider any adaptation measure to seawater intrusion through the agricultural development plan. Therefore, among the main objectives of this study is to investigate the impact of abstraction on the expected groundwater drawdown in the Moghra aquifer. The second objective is to simulate the impact of various abstraction schemes in agricultural development areas on seawater intrusion in the Moghra aquifer. The current study presents integrated research with a simulation of the flowing of groundwater and the invasion of saltwater, taking different agricultural cultivation scenarios into consideration, and provides different plans and scenarios for controlling saltwater intrusion in the Moghra aquifer for better management of the Moghra aquifer’s groundwater to sustain the agriculture ecosystem in the study area.

## Methodology

**Fig. 1:**
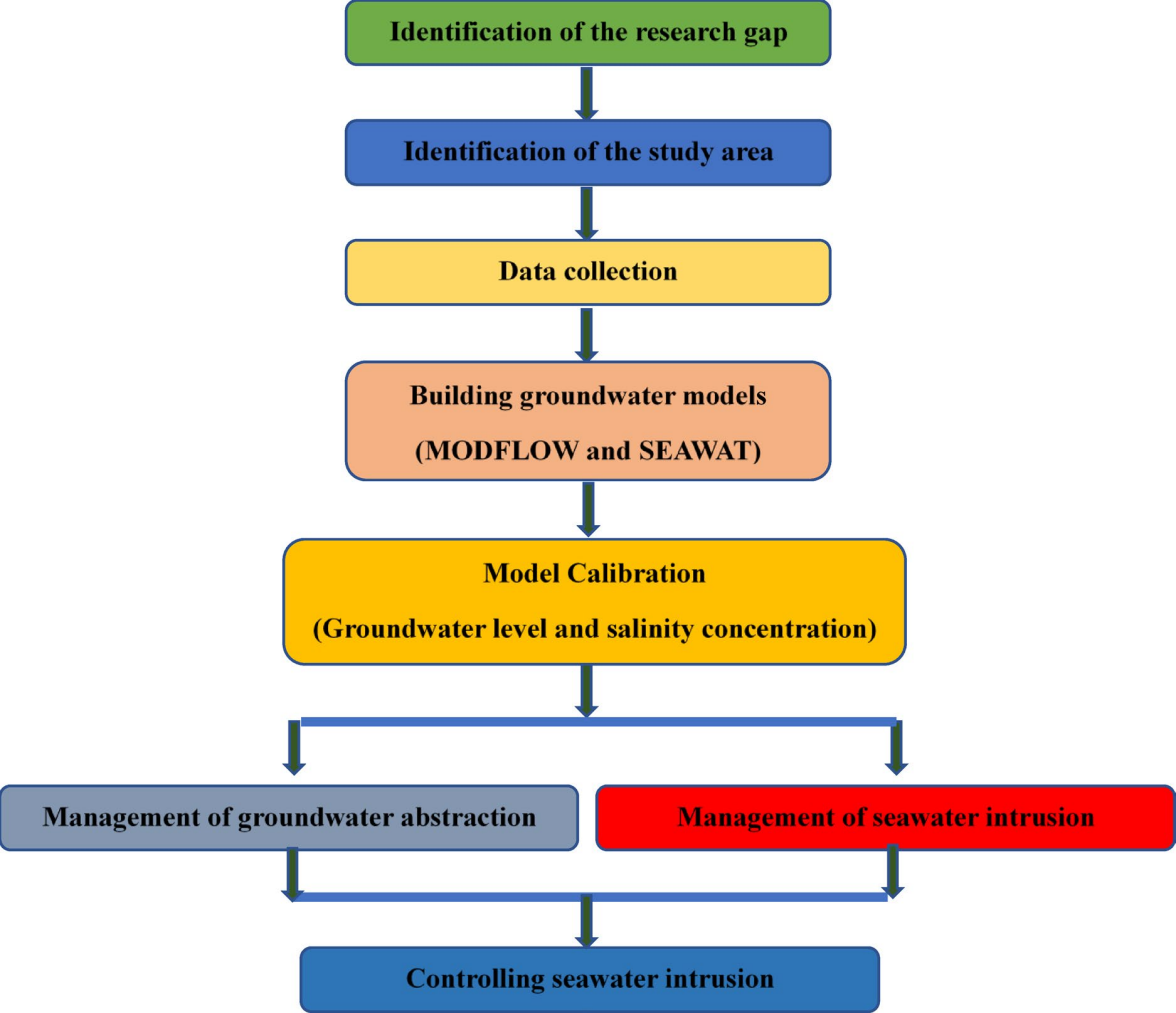
Proposed research study flow chart.

Figure 1 represents the research study flowchart which will be explained in the next subsections. The proposed methodology of the current research includes identification of the research gap, identification of the study area, data collection, building the groundwater models using MODFLOW and SEAWAT, model calibration, proposed pumping schemes scenarios, management of groundwater pumping, management of seawater intrusion, and regulating the Moghra aquifer’s saltwater invasion.

## Study area

**Fig. 2:**
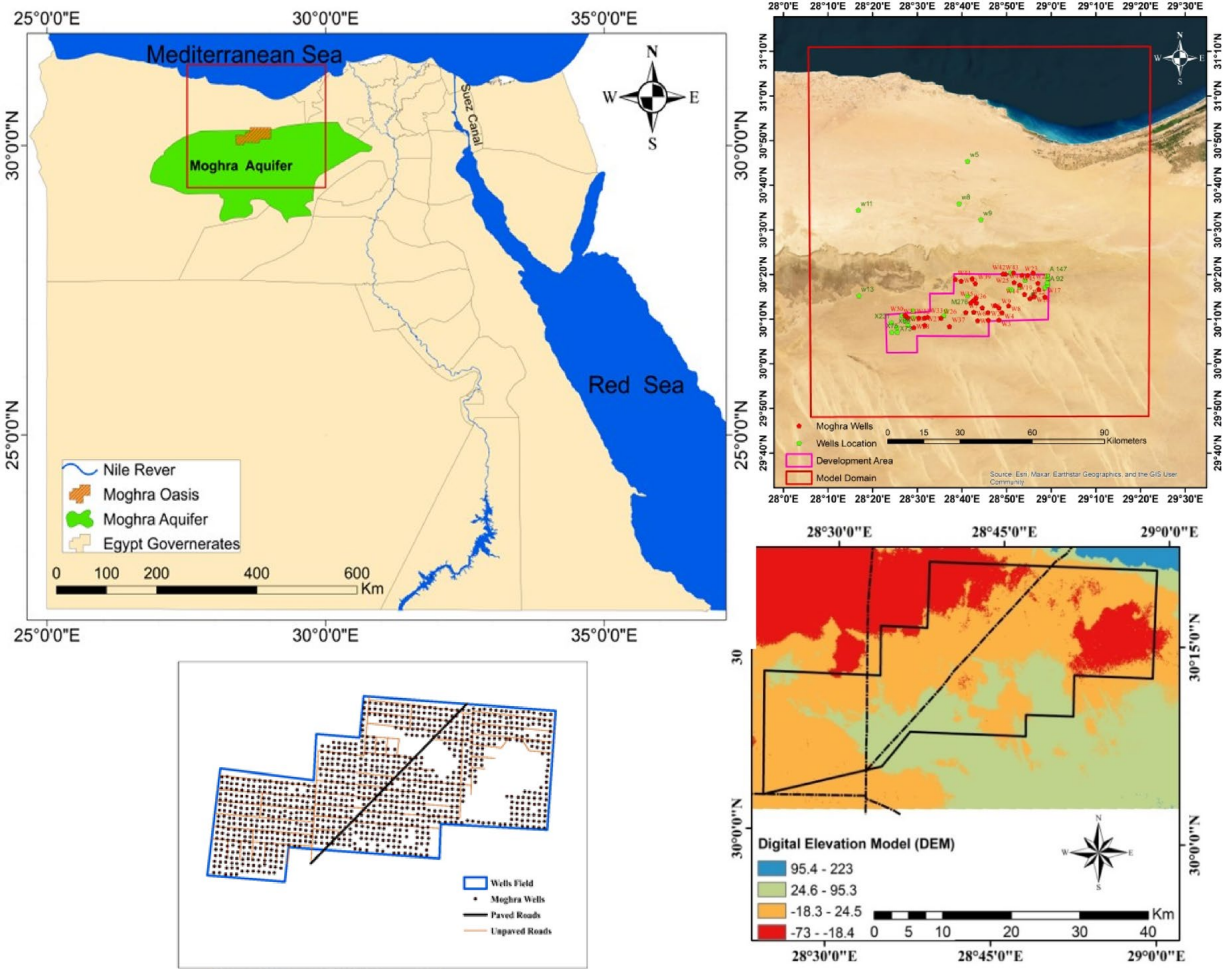
Location map, wells locations, sample locations, and DEM of the study area.

The study area is the Moghra aquifer. It was selected due its importance, as about 170 million feddans in the Moghra region are proposed to be cultivated as a part of a mega cultivation project in Egypt based on groundwater as a primary source. The research region is located in Egypt’s northernmost region of the Western Desert. It spans the geographic locations of 29°50′, 31°10′ N latitude and 28°10′, 29°20′ E longitude. Looking south, the agricultural development area based on groundwater abstraction is located around 60 km away from the shore (Fig. 2).

The Moghra aquifer system, a key water-bearing formation in the northeastern part of Egypt’s Western Desert, is composed primarily of Miocene-era fluviatile sediments, as well as coarse marine sand and gravel ^37^. This aquifer is thickest in its central region, reaching approximately 900 m, but it gradually thins as it extends westward and northward, where normal fault lines are prevalent ^38^. The average thickness of the water-bearing layer across the system is about 300 m. Generally, the Moghra aquifer is unconfined, meaning that its water is freely flowing. However, it becomes confined in the northern regions, where it is overlaid by limestone from the Marmarica aquifer, which lies atop Miocene deposits ^39^. Faults are visible on both the eastern and western sides of the aquifer, near the Wadi El-Farigh and Qattara depressions, respectively.

The water table in the Moghra aquifer varies significantly, with levels ranging from approximately 20 m below mean sea level (b.m.s.l) near El-Dabaa Road to 50 m (b.m.s.l) close to the Qattara depression in the west. This gradient causes the water to flow from the northeast towards the southwest, with the Qattara depression acting as a natural discharge point where a sharp drop in water levels occurs. The quality of water in the Moghra aquifer also varies, ranging from freshwater with total dissolved solids (TDS) of 1,000 ppm or less in a narrow eastern strip to saline water with TDS levels up to 12,000 ppm across much of the aquifer’s area ^40^. The research region is in the subtropical Mediterranean climate’s northwest coastal zone, which has hot, dry summers and mild, rainy winters. Winter precipitation in Matrouh averages 155 mm per year ^41–43^.

The groundwater in the study area is stored primarily in four major aquifers dating from the Tertiary and Quaternary periods ^44^. In the northern region, a complex system of three hydraulically connected aquifers is notably affected by seawater intrusion ^18,45,46^. Due to uncommon occurrences in the study region, the subsequent characteristics are only of secondary relevance in this region. The Holocene Coastal Dune Aquifer, composed of unconfined white carbonate sands, is recharged by annual rainfall and stretches along the Mediterranean coast, covering about 13% (2,000 km^2^) of the area with a thickness exceeding 30 m ^47^. Beneath it lies the Pliocene Limestone Aquifer, a Tertiary unit composed of massive limestone, partly confined and partly unconfined, replenished by rainfall, covering roughly 30% (4,480 km^2^) of the area ^45^. Below that, the Upper Miocene Marmarica Aquifer, largely untapped by wells, spans 6,500 km^2^ (43% of the area) with a thickness of 50–250 m. It contains unconfined water that is recharged both by rainfall and leakage from overlying aquifers, especially through the limestone ridges that facilitate annual infiltration from the surrounding highlands ^47,48^.

**Fig. 3:**
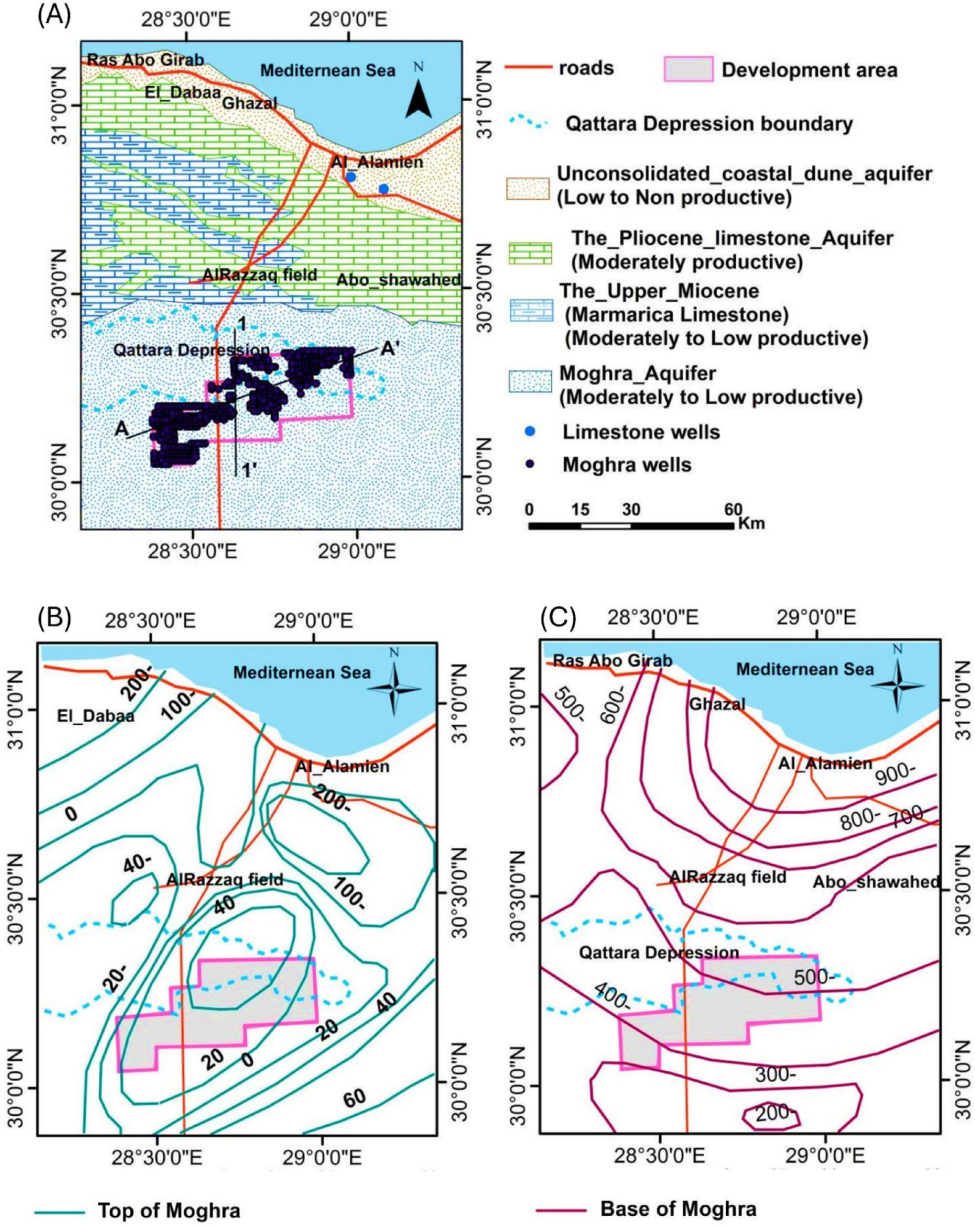
(**A**) The study region aquifers, (**B**) The Moghra groundwater aquifer top, and (**C**) The Moghra aquifer bottom after Ezzat ^11^ and RIGW ^51^.

In the southern part of the research area, the Moghra aquifer comes to the surface, close to the development region, wherein all these aquifers tend to vanish. The Moghra aquifer (Lower Miocene Aquifer) is made up of coarse sand lenses and sandstone with shale intercalations ^49^. According to Abdul Mogith et al. ^48^, it is the primary aquifer system of the research region, with thicknesses ranging from 0 to 500 and a base of Oligocene Dabaa shale. It covers an area of 50,000 km^2^ and is bound to the east by the Mediterranean coast, to the north by the Nile Delta, and to the south by the Bahariya–Abu Roash uplift. It stretches widely from the north to the Western Desert ^45^. The Moghra aquifer extends beyond the geographical domain but covers the entire area under study. In the southern portion of the region close to the development area, the aquifer functions as an unconfined aquifer; nevertheless, in the northern part, it is subject to confined circumstances (^50^; Fig. 3).

## Data collection, analysis, and sampling

In this study, the Ministry of Water Resources and Irrigation investigated around 450 wells drilled in 2018 in only two of the aquifers mentioned above (Table 1). The level of groundwater in the Moghra aquifer system ranged from − 10 m to − 60 m, with the primary flux flowing between the northeast and the southwest. To determine the salinity of the aquifer water, approximately 96 samples were collected from the screens of the Moghra Aquifer system. Their salt concentration ranges from 1200 – 9800 mg/L. In contrast, previous data from only 20 wells dispersed throughout the aquifer demonstrates that TDS varied between 3300 to 10,100 mg/L. This information contributed to the development of a salinity concentration profile for the Moghra groundwater aquifer. The Pliocene limestone aquifer, on the other hand, has water levels ranging from 6 to 24 m. The flow comes from the south direction to the sea in the north direction. It is noteworthy that most of the samples from Pliocene groundwater aquifers contain saltwater at 42,000 mg/L. These samples were too shallow, collected at depths ranging from 20 to 40 m; just one samples exhibited a TDS of 8813 mg/l. A broad variety of salinity fluctuations was identified in the water samples collected from the wells dug into the system of aquifers, proving that the aquifer system’s groundwater is brackish, as categorized by Davis and De Wiest’s ^52^, as illustrated in Table 2. This information was used to simulate the aquifer system and produce the first water quality picture. The locations of the case study wells and samples, in addition to the aquifers they pierce, are distributed as shown in Fig. 1. To observe the current salinity concentration in the Moghra aquifer after starting the pumping abstraction from 445 wells to fulfill the water requirement for cultivating about 135 feddans in the first stage of the project, 45 groundwater samples from wells were collected from the Moghra aquifer through a field trip by the authors in the year 2022 ^18^. According to a recent evaluation of groundwater samples, the salinity concentration in the agricultural development area in the Moghra aquifer ranged between 2446 to 8021 mg/L ^18^.

**Table 1:** Wells situated inside the case study’s domain ^11,18,50^.

No. of wells		Well no	Penetrated aquifer	Total depth (m)	D.T.W (m)	Water level (m)	TDS (mg/L)	Q (m^3^/h)
450 wells	New samples	445	Moghra	90–200	30 to 90	− 10 to − 60	1200 to 9800	–
		5	Pliocene	15–110	2.1to18.8	6 to 24	8813 to 42,000	–
20 wells	Literature		Moghra	275–985	− 1 to 29.7	− 26 to − 48.7	3300 to 10,100	8:108

**Table 2:** The expected drawdown (m) in the Moghra region for 100 years of agricultural development for 8 scenarios (Daily water duty = 14 m^3^/acre = 13.48 m^3^/feddans*).

Scenario No	No. of wells	Abstraction for each well m^3^/day/well	Total abstraction Mm^3^/day	Targeted cultivation area (Feddans)	Drawdown (m) for 100 years of agricultural development									
					10	20	30	40	50	60	70	80	90	100
1	445	1000	0.445	30,622	7.5	11	13	15	17	18	21	22	23	24
2		1250	0.556	38,260	10	14	17	20	22	24	25	27	28	29
3		1500	0.667	45,898	11	17	21	24	26	28	30	34	35	36
4		1750	0.788	54,224	12	17	22	26	29	31	33	35	37	39
5	1000	1000	1.00	68,813	16	24	30	36	40	44	46	50	53	54
6		1250	1.25	86,017	20	30	38	44	50	54	58	60	64	66
7		1500	1.50	103,220	24	38	48	54	58	65	70	76	80	85
8		1750	1.75	120,423	28	43	53	62	69	73	80	85	92	100

*1 Feddans = 1.038 acre = 0.42 Hectare.

## Groundwater model building

**Fig. 4:**
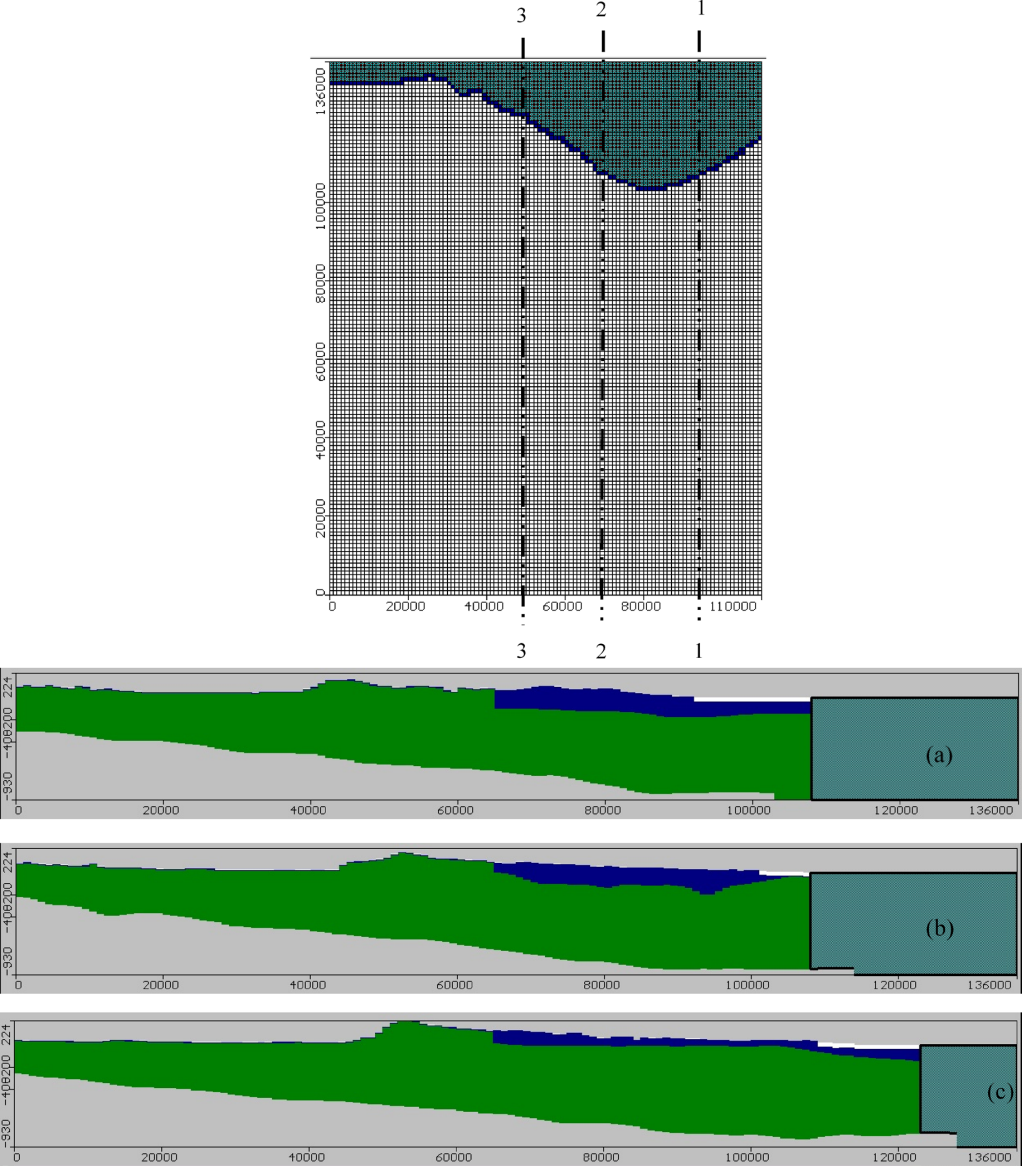
(**a**) 2D view of the model grid for the Moghra aquifer and three vertical cross-sections "Introduction" (**b**), 2 (**c**), and 3 (**d**) located in eastern, central and western parts (White, blue, and green colors indicate Pliocene, Marmarica, and Moghra layers, respectively).

Management of the Moghra aquifer in relation to the occurrence of SWI is viewed as a difficult procedure. Apart from considering the complexity of modeling the procedure, the aquifer in the research region was pumped after the start of cultivation. The salinity circumstances in Moghra are critical since the groundwater flow pattern is identical to that of the saltwater, a circumstance known to be active SWI, indicating that there is a high risk of incursion during pumping. As a result, a numerical model was created to simulate case scenarios. Utilizing the SEAWAT model, a three-dimensional model for the flowing of groundwater and solute transport was established to simulate groundwater flow and saltwater intrusion for the aquifer system while taking into account all available hydrogeological features and model calibration, and to estimate the aquifer dynamic behavior according to various pumping circumstances. The SEAWAT module consists of two modules. The initial module, MODFLOW, is employed to handle the flow of groundwater and direction of movement issues, while the following one, MT3DMS, is utilized to tackle transportation difficulties ^53^. A grid with a cell size of 1000 m (in x direction) × 1000 m (in y direction) and 136 rows × 110 columns across the horizontal plane covers the simulated region. The domain in the vertical direction was separated into six layers, each of which has 89,760 cells having various hydrogeological properties. The initial layer is representative of the Pliocene layer, the second layer represents the Marmarica Limestone layer, and the third layer to the sixth layer represents the Moghra aquifer. The fact that the Mediterranean Sea has a constant salinity limit of 35,000 mg/L and a constant head border of 0 m was taken into consideration. Having thicknesses of 38 and 250 m, respectively, the first and second layers correspond to the limestone aquifers of Pliocene and Upper Miocene limestone. The Moghra aquifer, represented by layers three to six, is 500 m thick and extends 980 m in the northern direction of the research area. Figure 4 shows a 2D view of the model grid for the study area and three cross-sections in the vertical direction of the groundwater model domain to represent salinity in the vertical direction. Cross-sections "Introduction", "Methodology", and "Study Area" are located in the eastern, central, and western areas of the Moghra area.

### Hydraulic parameters of the Moghra aquifer

Aquifer Test 2016.1 software was used to analyze pumping tests on a number of chosen wells and determine the Moghra hydraulic characteristics. The transmissivity values vary between 760 and 7600 m^2^/d, and the values of hydraulic conductivity range from 1 m/d to 24 m/d. The range of the storativity is 0.1 to 0.001. In contrast, the Pliocene limestone has a value of hydraulic conductivity of 9.1–39.8 m/d and the Marmarica aquifer has a value of hydraulic conductivity of 0.1–6 m/d ^11,54^. Regarding conservative saline transport, the developed model took into account the advection and hydrodynamic dispersion mechanisms. The longitudinal dispersivity (grid spacing) for the built model was 1000 m. The longitudinal dispersivity was 0.01 and 0.1 for the built model of the transversal vertical and horizontal dispersivities, correspondingly. According to Korany ^55^, the porosity of the sandy shales and the Moghra sandstone is 30%:58% and 26%:50%, correspondingly.

### Studied scenarios

The Groundwater Sector of the Ministry of Irrigation and Water Resources, in collaboration with the Egyptian Countryside Development Company, has drilled around 445 wells to meet the development’s water demand. The planned development region is around 60 km from the Mediterranean Sea, and there is a potential that seawater will enter the fresh zone of the Moghra aquifer as a result of continuous pumping. The present research will demonstrate how pumping can accelerate the degree of seawater invasion inland into the Moghra aquifer. About 445 pumping wells were drilled in the Moghra aquifer with total variable depth ranging from 90 to 200 m, and the depth to groundwater water ranged from 3 to 90 m ^56^. The aquifer is discharged by pumping groundwater for irrigation from already-existing wells and water delivery. In 1980, 20 wells were sunk over the aquifer extension in the research region domain using low abstraction rates. Recently, 445 wells have been drilled in the developing region domain in the southern half of the research region. They were not pumped until the end of 2020. They will be planned and implemented to serve an area of 240,000 feddans. The groundwater will be utilized for residential, irrigation, and industrial uses. The daily discharge rate will be 100 m^3^/h with 10 operating hours per day (163 MCuM/year), and they will be powered by a solar power system in accordance with the Egyptian Countryside Development Company’s guidelines. The aquifer also empties into adjacent reservoirs in the southwest via evaporation from free circumstances, waterlogged lands, cultivated regions, and subsurface flow ^14^. For Moghra, 20 wells from 1983 were pumped at modest rates and utilized for demonstration purposes. The new development area is ready to absorb the Moghra aquifer. As the project’s first stage, 445 extraction wells were drilled between 2016 and 2019 in the development area with the goal of extracting around 163MCuM/year, but they were not pumped until 2020; they will serve as observation wells till the project is operational ^20^. A development expansion of 550 wells is proposed ^57^. It is worth noting that the wells’ field was located in the proper place for such a growing project according to economic and environmental standards ^7^. Seven scenarios of groundwater abstraction were experienced to check the influence of the groundwater abstraction schemes on the expected drawdown in the Moghra region. It is expected that the total number of groundwater wells will be increased to reach 1000 wells to fulfill the agricultural development. For a total of 445 wells, four scenarios were considered: 1000 m^3^/day/well, 1250 m^3^/day/well, 1500 m^3^/day/well, and 1750 m^3^/day/well. For a total of 1000 wells, four scenarios were considered: 1000, 1250, 1500, and 1750 m^3^/day/well.

## Results

### Results of model calibration

**Fig. 5:**
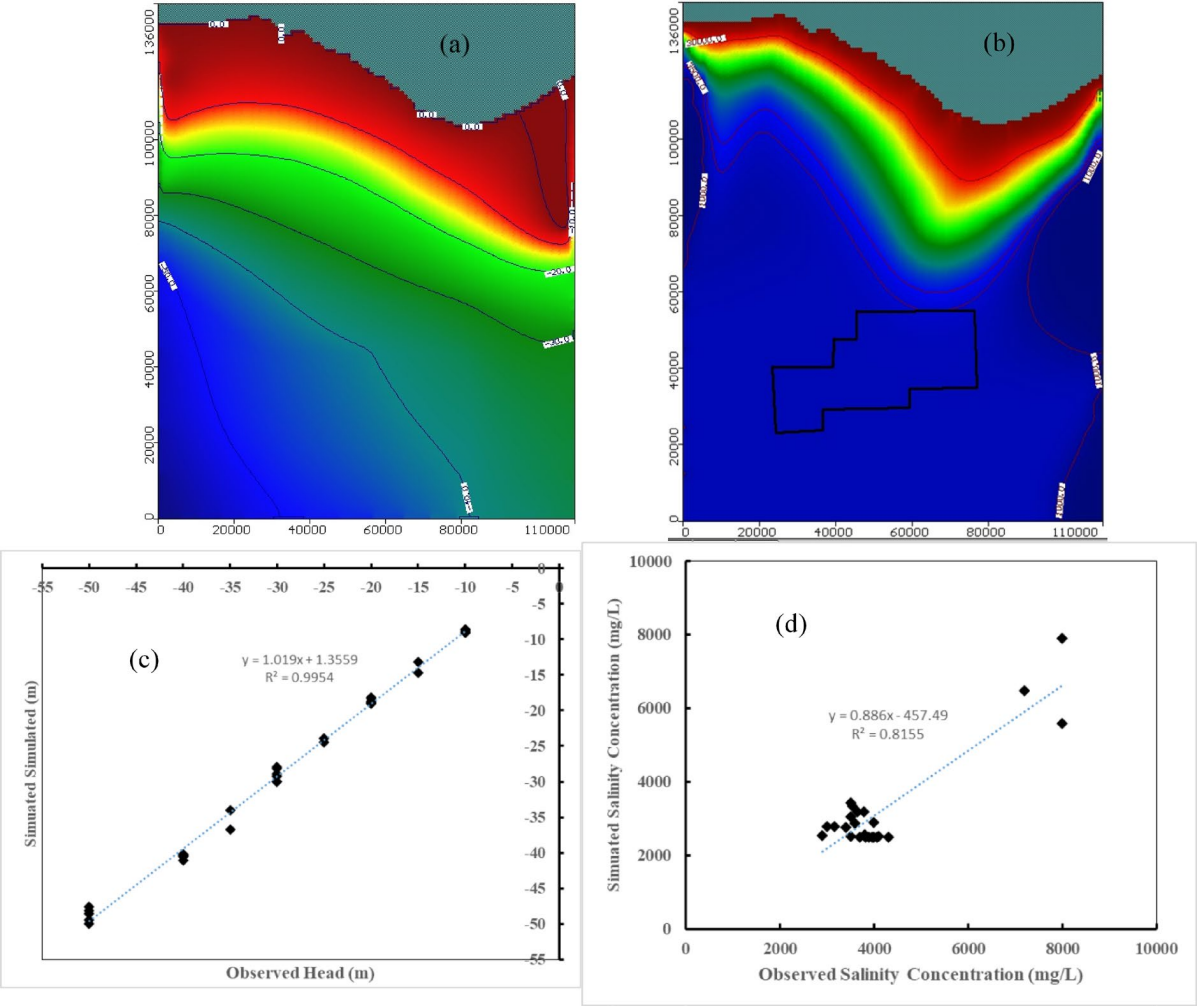
Simulation results for steady state: (**a**) Groundwater level in the Moghra aquifer, (**b**) Salinity distribution in the Moghra aquifer, (**c**) Simulated versus observed groundwater level, and (**d**) Simulated versus observed salinity concentration.

The simulated results of MODFLOW and SEAWAT were contrasted with observed data based on the literature for model calibration. The simulated groundwater level for 35 wells distributed in the Moghra region was compared with the observed groundwater level by the Research Institute of Groundwater for Egypt. The simulated salinity concentration for 30 locations was contrasted with the observed salt concentration for model validation. Figure 5 shows the simulation results for steady-state conditions for groundwater level in the Moghra aquifer, salt concentration distribution in the Moghra aquifer, simulated versus observed groundwater level, and simulated versus observed salinity concentration. A comparison between the simulated and measured groundwater levels shows a good agreement for both groundwater level and salinity concentration. The correlation coefficient between the measured and simulated results equal 0.998 and 0.903 for groundwater level and salinity concentration, respectively.

### Management of groundwater abstraction in the Moghra coastal aquifer

In scenario S1, there are 445 groundwater wells in total, and the abstraction rate equals 1000 m^3^/day/well. The total abstraction rate equals 0.445 × 10^6^ m^3^/day through 100 years of the cultivation project. The level of groundwater in the Moghra aquifer declined towards the south, ranging from 0.0 m in the northern coastal area to reach − 50.8 m after 10 years for the first scenario. The level of groundwater ranged from 0.0 to − 51 m, 0.0 to − 53 m, 0.0 to − 55 m, and 0.0 to − 56.25m after 20, 30, 40, and 50 years from the project’s start, respectively. In addition, the groundwater level ranged from 0.0 to − 57.6m, 0.0 to − 58.8 m, 0.0 to − 59.8 m, and 0.0 to − 60.5m, after 60, 70, 80, and 90 years, respectively. After 100 years of cultivation in the first scenario, the groundwater level reached –61.6 m in the south region of the Moghra groundwater aquifer in the middle area of cultivation. The total number of wells in this scenario is 445. The drawdown of groundwater level increased over time in the study area with an abstraction rate equal to 1000 m^3^/day/well, and the maximum drawdown is observed in the agricultural development area due to the groundwater abstraction. The drawdown reached 7.5, 11, 13, 15.5, 17.5, 18.5, 21, 22, 23, and 24m after 10, 20, 30, 40, 50, 60, 70, 80, 90, and 100 years; thus, the maximum drawdown for scenario No. 1 equals 24m, which is observed after 100 years of cultivation in the Moghra region, based on the groundwater as the main source of agricultural development. In Scenario S2, there are 445 wells in total, and the abstraction rate per well is 1250 m^3^/day. A total of 0.556 × 10^6^ m^3^/day (203 × 10^6^ m^3^/year) is the abstraction rate. From 0.0 m in the northern coastal region to − 52 m after 10 years for the first scenario, the groundwater level in the Moghra aquifer decreases southward. After 20, 30, 40, and 50 years from the project’s start, the level of groundwater varies from 0.0 to − 52.9 m, 0.0 to − 55.7 m, 0.0 to − 57.05 m, and 0.0 to − 59.8 m, respectively. Furthermore, after 60, 70, 80, and 90 years, the groundwater level varies from 0.0 to − 61.1 m, 0.0 to − 62.4 m, 0.0 to − 63.6 m, and 0.0 to − 65.03 m, respectively. In the second scenario, with 445 total wells, the groundwater level in the southern regions of the Moghra aquifer system in the middle area of cultivation dropped to − 66.3 m after 100 years of cultivation. As groundwater abstraction increased to 1250 m^3^/day/well, the study area’s groundwater level drawdown grew with time. The agricultural development area saw the greatest drawdown as a result of groundwater abstraction. After 10, 20, 30, 40, 50, 60, 70, 80, 90, and 100 years, the drawdown reached 10, 14, 17, 20, 22, 24, 25, 27, 28, and 29 m, respectively. After 100 years of agriculture, the maximum decline is 29 m. In the Moghra region, groundwater serves as the primary source of agricultural development in scenario No. 2.

In scenario S3, the total number of wells is again 445, and the abstraction rate per well is 1500 m^3^/day. A total abstraction rate of 243 × 10^6^ m^3^/year, or 0.667 × 10^6^ m^3^/day, is achieved. Beginning at 0.0 m in the northern coastal region, the level of groundwater in the Moghra aquifer decreases southward, reaching − 50.8 m after 10 years for the third scenario. The groundwater levels at 20, 30, 40, and 50 years following the project’s start are 0.0 to − 54.90 m, 0.0 to − 58.50 m, 0.0 to − 60.80 m, and 0.0 to − 63.10 m, respectively. Furthermore, the groundwater level varies after 60, 70, 80, and 90 years, respectively, from 0.0 to − 65.8 m, 0.0 to − 67.25 m, 0.0 to − 69.3 m, and 0.0 to − 70.1 m. The groundwater level in the southern parts of the Moghra aquifer in the middle region of farming dropped to − 72.05 m after 100 years of cultivation in the third scenario. As groundwater abstraction increased to 1500 m^3^/day/well, the drawdown of the study area’s groundwater level grew with time. The agricultural development area saw the greatest drawdown as a result of groundwater abstraction. After 10, 20, 30, 40, 50, 60, 70, 80, 90, and 100 years, the drawdown reached 11, 17, 21, 24, 26, 28, 30, 34, 35, and 36 m, respectively. The maximum drawdown equals 36 m, which is observed after 100 years of cultivation. For scenario S3, in the Moghra region, groundwater is the main source of agricultural development. Under scenario S4, there are again 445 wells in total. As groundwater abstraction increased to 1750 m^3^/day/well, the study area’s groundwater drawdown level grew with time. The agricultural development area saw the greatest drawdown as a result of groundwater abstraction. After 10, 20, 30, 40, 50, 60, 70, 80, 90, and 100 years, the drawdown reached 12, 17, 22, 26, 29, 31, 33, 35, 37, and 39 m, respectively. The maximum drawdown equals 39 m, which is observed after 100 years of cultivation. For scenario S4, in the Moghra region, groundwater is the main source of agricultural development.

In scenario S5, the abstraction rate in this case is 1000 m^3^/day/well, and there are 1000 wells in total. The abstraction rate is a total of 1 × 10^6^ m^3^/day (365 × 10^6^ m^3^/year). Starting at 0.0 m in the northern coastal region, the groundwater level in the Moghra aquifer decreases southward, reaching − 55.5 m after 10 years for the fifth scenario. After 20, 30, 40, and 50 years from the project’s start, the groundwater level varies from 0.0 to − 61.5 m, 0.0 to − 67.6 m, 0.0 to − 71.9 m, and 0.0 to − 75.60 m, respectively. The level of groundwater also varies after 60, 70, 80, and 90 years, from 0.0 to − 78.8 m, 0.0 to − 81.3 m, 0.0 to − 84.70 m, and 0.0 to − 86.4 m, respectively. Following a century of cultivation, the fifth scenario’s groundwater level in the southern Moghra aquifer region in the middle cultivation area was − 89 m. In the current scenario, there are now 1000 wells in total. The study region’s groundwater level decline grew over time as the groundwater abstraction reached 1000 m^3^/day/well; the agricultural development area saw the largest drawdown because of the groundwater extraction. The drawdown occurred after 10, 20, 30, 40, 50, 60, 70, 80, 90, and 100 years and reached 16, 24, 30, 36, 40, 44, 46, 50, 53, and 54 m, respectively. The maximum decline, which is seen after 100 years of farming, is 54 m. In scenario S5, the principal source of agricultural development in the Moghra region is groundwater.

In scenario S6, there are 1000 wells in total, and the abstraction rate is 1250 m^3^/day/well. An abstraction rate of 1.25 × 10^6^ m^3^/day (456 × 10^6^ m^3^/yr) is the total. At 0.0 m in the northern coastal region, the Moghra aquifer’s groundwater level drops southward, reaching − 56.7 m in the sixth scenario after 10 years. After 20, 30, 40, and 50 years from the start of the project, the level of groundwater varies from 0.0 to − 65.7 m, 0.0 to − 73.9 m, 0.0 to − 80.30 m, and 0.0 to − 85.20 m, respectively. Furthermore, after 60, 70, 80, and 90 years, the level of groundwater varies from 0.0 to − 89.1 m, 0.0 to − 92.8 m, 0.0 to − 96.4 m, and 0.0 to − 99.3 m, respectively. In the sixth scenario, the groundwater level in the southern parts of the Moghra aquifer in the middle region of cultivation dropped to − 101.9 m after 100 years of cultivation. As groundwater abstraction increased to 1250 m^3^/day/well, the study area’s groundwater level drawdown grew with time. The agricultural development area saw the greatest drawdown as a result of groundwater abstraction. After 10, 20, 30, 40, 50, 60, 70, 80, 90, and 100 years, the drawdown increased to 20, 30, 38, 44, 50, 54, 58, 60, 64, and 66 m. Based on groundwater as the primary source of agricultural development, S6 in the Moghra region shows a maximum drawdown of 66 m after 100 years of farming.

In scenario S7, the abstraction rate in this case is 1500 m^3^/day/well, and there are 1000 wells in total. An abstraction rate of 1.5 × 10^6^ m^3^/day (547 × 10^6^ m^3^/yr) is the total. Groundwater level maps were displayed every 10 years, and Fig. A.1 shows the Moghra aquifer’s groundwater level for scenario S7 for the course of the agriculture project’s 100 years. After 10 years, the Moghra aquifer’s groundwater level drops southward from 0.0 m in the northern coastal region to − 61.1 m in the seventh scenarios. Then 20, 30, 40, and 50 years after the project’s start, the groundwater level varies from 0.0 to − 72.8 m, 0.0 to − 81.7 m, 0.0 to − 88.6 m, and 0.0 to − 94.4 m, respectively. The level of groundwater also varies after 60, 70, 80, and 90 years, from 0.0 to − 100 m, 0.0 to − 103.08 m, 0.0 to − 106.5 m, and 0.0 to − 110 m, respectively. Following a century of farming, the seventh scenario’s groundwater level in the southern Moghra aquifer region in the middle cultivation area dropped to − 115 m. The Moghra aquifer’s groundwater level decline for scenario No. 7 is shown in Fig. A.2 every 10 years. In this scenario, there are now 1000 wells overall. As groundwater abstraction increased to 1500 m^3^/day/well, the study area’s groundwater level drawdown grew with time. Groundwater abstraction resulted in the largest decline in the agricultural development area. After 10, 20, 30, 40, 50, 60, 70, 80, 90, and 100 years, the drawdown increased to 24, 38, 48, 54, 58, 65, 70, 76, 80, and 85 m. After a century of agriculture, the maximum drawdown for S7 is 85 m.

**Fig. 6:**
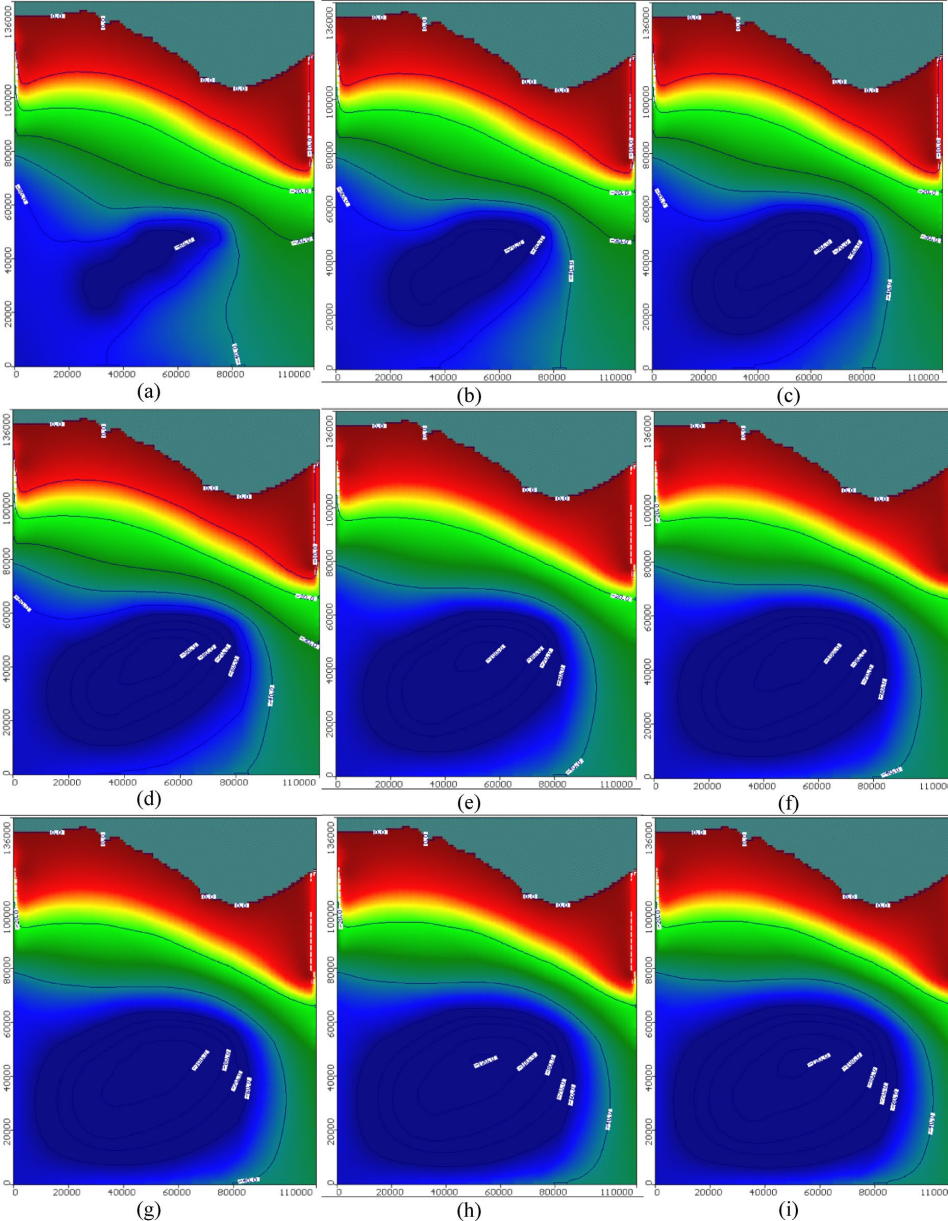
Simulation of groundwater level in the Moghra aquifer for scenario No. 8: 1000 wells with abstraction rate 1750 m^3^/day/well through 100 years test period: (**a**) 10, (**b**) 20, (**c**) 30, (**d**) 40, (**e**) 50, (**f**) 60, (**g**) 70, (**h**) 80, (**i**) 90, and (**j**) 100 years.

In scenario S8, the total number of wells is 1000, and the abstraction rate equals 1750 m^3^/day/well. The total abstraction rate equals 1.75 × 10^6^ m^3^/day (638 × 10^6^ m^3^/yar). Figure 6 describes the groundwater level in the Moghra aquifer for scenario No.8 through 100 years of the cultivation project, and the groundwater level maps were shown for each 10 years. The level of groundwater in the Moghra aquifer declined towards the south, ranging from 0.0 m in the northern coastal area, and declined towards the south to reach − 63.4 m after 10 years for the eight scenario. The level of groundwater ranged from 0.0 to − 87.14 m, 0.0 to − 88.3 m, 0.0 to − 97.01 m, and 0.0 to − 104.4m after 20, 30, 40, and 50 years from the beginning of the project, respectively. In addition, the groundwater level ranged from 0.0 to − 108.5m, 0.0 to − 115.6 m, 0.0 to − 120.8 m, and 0.0 to − 125.6 m, after 60, 70, 80, and 90 years, respectively. After 100 years of cultivation, in the first scenario, the level of groundwater reached –130 m in the south region of the Moghra aquifer in the middle area of cultivation.

**Fig. 7:**
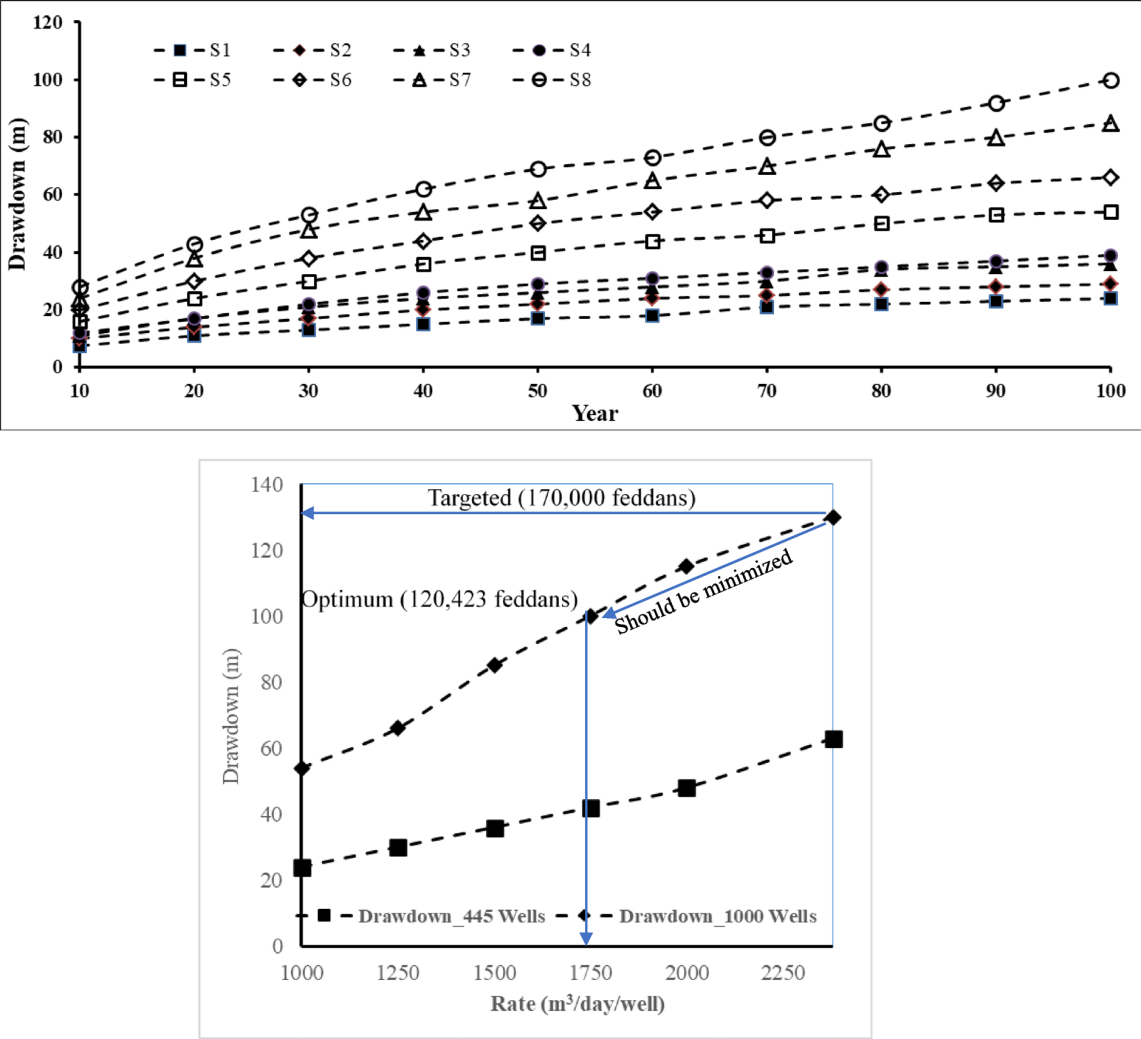
The drawdown in groundwater level in the Moghra aquifer for eight different abstraction scenarios for 100 years as a test period.

Table 2 and Fig. 7 present the drawdown in groundwater level in the Moghra aquifer for eight different abstraction scenarios for 100 years as a test period. The first four scenarios have a total number of wells equal to 445 wells for each scenario with various tested abstraction rates equal to 0.445, 0.556, 0.667, and 0.788 Mm^3^/day, and the proposed cultivated area equals 30,622, 38,260, 45,898, and 54,224 feddans, respectively. The fifth, six, seventh, and eighth scenarios have a total number of wells equal to 1000 for each scenario with various tested abstraction rates equals 1.00, 1.25, 1.50, and 1.75 Mm^3^/day, and the proposed cultivated area equals 68,813, 86,017, 103,220, and 120,423 feddans, respectively. Scenario No. 8 presents the optimum scenario. After a 100− year test period, the maximum drawdown equals 100 m within the maximum limit proposed by the MWRI of Egypt, and the corresponding cultivated land equals 120,423 feddans.

Increasing the rate of abstraction from 1750 m^3^/day/well to 2000 m^3^/day/well led to an increase in the maximum drawdown to 115 m, which exceeds the maximum acceptable drawdown (100m) by the MWRI of Egypt. Over a span of 100 years, 170,000 feddans were proposed to be cultivated in the Moghra aquifer. This scenario required an abstraction rate equal to 2.38Mm^3^/day, and the expected drawdown will would reach 130 m, exceeding the maximum limit (100 m). The proposed plan by the Egyptian government should minimize the area of cultivation from 170,000 feddans to 120,423 feddans, which achieves the optimum scenario with an acceptable maximum drawdown of 100 m over 100 years.

### Saltwater intrusion management in the Moghra coastal aquifer and proposed mitigation measures

**Fig. 8:**
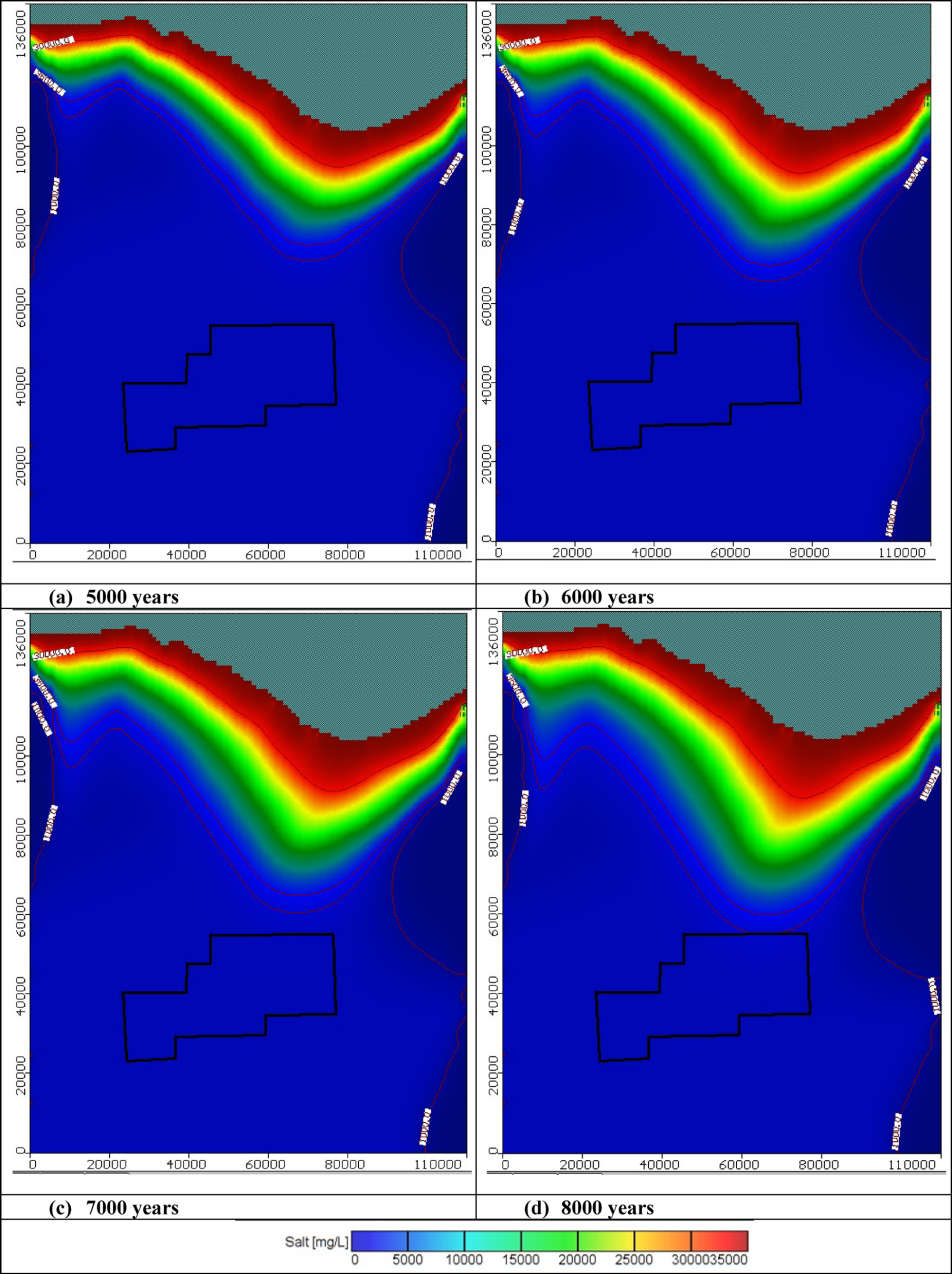
Advancement of seawater distribution in the Moghra aquifer for years: (**a**) 1000, (**b**) 2000, (**c**) 3000, (**d**) 4000, (**e**) 5000, (**f**) 6000, (**g**) 7000, and (**h**) 8000 years.

**Fig. 9:**
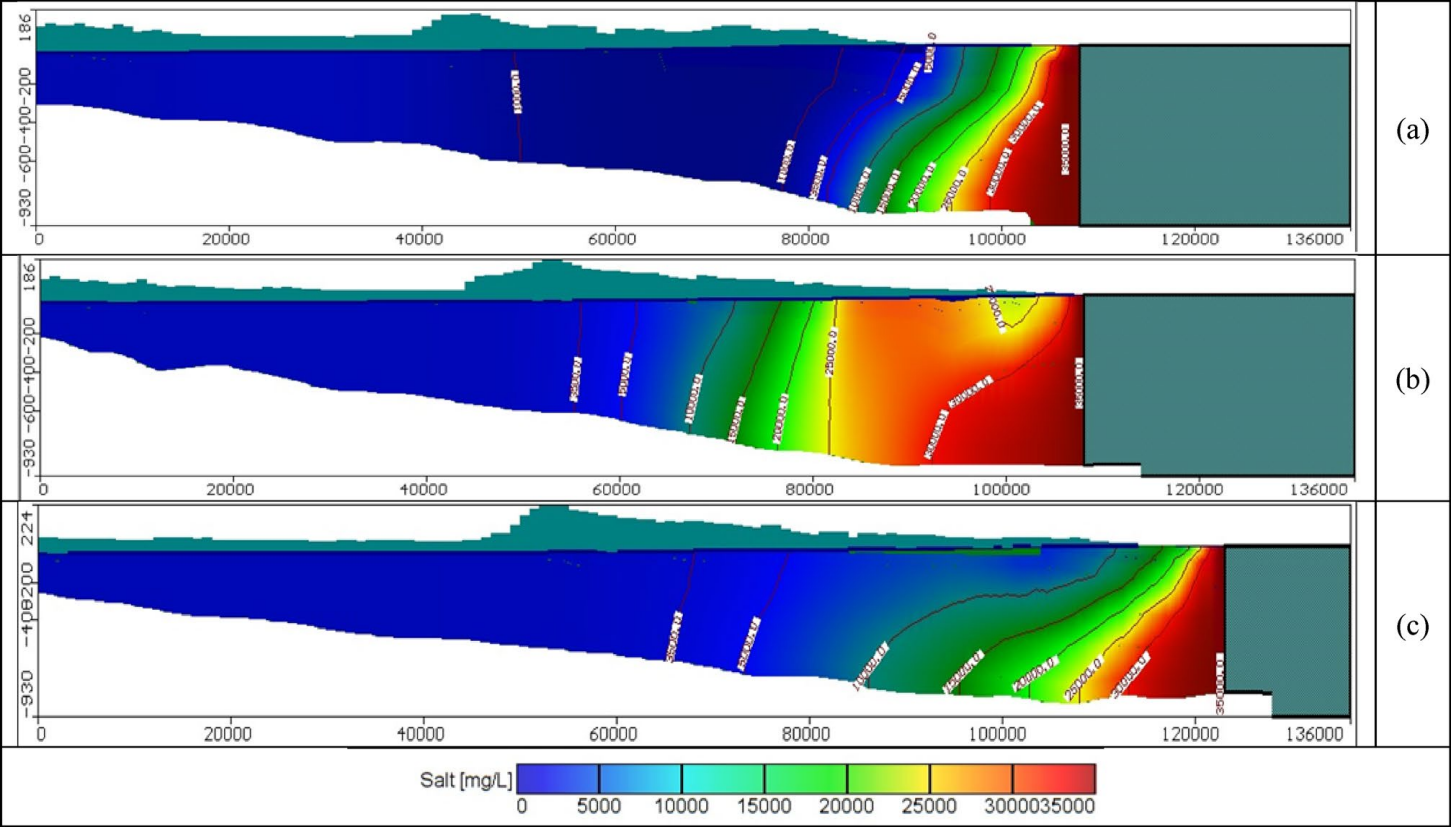
Steady state seawater distribution in cross-sections: (**a**) No. 1, (**b**) No. 2, and (**c**) No. 3 in the Moghra aquifer.

Figure 8 shows the distribution of saltwater intrusion in the Moghra aquifer. The interface between seawater and freshwater reached a steady state after 8000 years. The seawater distribution was shown in horizontal and vertical directions. Three cross-sections were considered: cross-sections No. 1, 2, and 3 are in the eastern, central, and western areas of the Moghra region. Figures A.3, A.4, and A.5 display the seawater intrusion in three vertical cross-sections: cross-sections No. 1, 2, and 3, respectively. The length of cross-sections measured from the sea to the south boundary equals 108 km, 108km, and 123 km, respectively. For cross-section No. 1, to the east, the initial intrusion length of the seawater for the concentration counter lines 1000, 3500, 5000, 17,500, and 30,000 mg/L equals 30.7, 27.2, 26, 18.4, and 9.2 km, consequentially. For cross-section No. 2, in the central of the study area, the initial intrusion length of the seawater for the concentration counter lines 3500, 5000, 17,500, and 30,000 mg/L equals 57.2, 47.9, 33.9, and 15.75 km, consequentially. For cross-section No. 3, in the western of the Moghra study region, the initial intrusion length of the seawater for the concentration counter lines 3500, 5000, 17,500, and 30,000 mg/L equals 57.1, 49.8, 23.5, and 10 km, consequentially. From the seawater distribution in the plan and three vertical cross-sections, it can be observed that the seawater intruded more inland in the central and western parts than in the eastern areas of the Moghra aquifer. The salinity concentration in the developed agricultural area in the Moghra region ranged from 2145 mg/l in the eastern parts increasing towards the central and western parts to reach 2590 mg/l, and high salinity was observed in the northern main area at about 4160 mg/L. After the model reached the steady state, different abstraction scenarios were considered to check the influences of abstraction schemes on saltwater invasion in the Moghra aquifer. Different mitigation methods were utilized to mitigate saltwater intrusion in the Moghra aquifer. Three techniques were tested, including the extraction of saltwater, artificial recharge through wells, and finally, a combination of saltwater abstraction and artificial recharge. Figure 9 displays the steady state seawater distribution in cross-sections No. 1, No. 2, and No 0.3 located in the eastern, central, and western areas in the Moghra aquifer.

#### Impact of groundwater abstraction on seawater intrusion in the Moghra aquifer (scenarios no. 1, 2, 3 and 4)

**Fig. 10:**
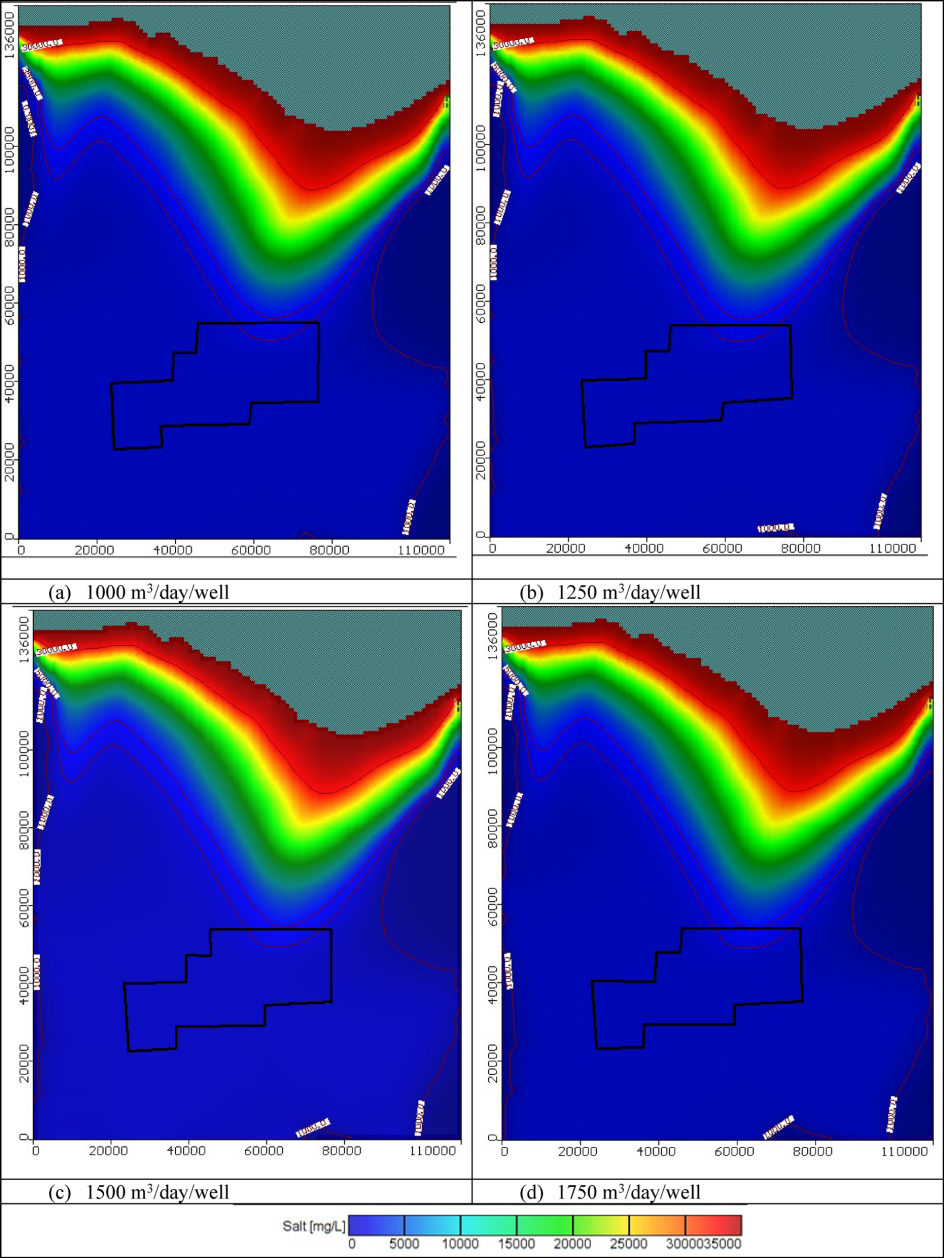
Seawater distribution in the Moghra aquifer for Scenarios 1 to 4 (445 wells): (**a**) 1000 m^3^/day/well, (**b**) 1250 m^3^/day/well, (**c**) 1500 m^3^/day/well, and (**d**) 1750 m^3^/day/well.

In these scenarios, the total number of wells is 445 and four abstraction rates of 1000, 1250, 1500, and 1750 m^3^/day/well were tested. The total abstraction rate equals 0.445 × 10^6^, 0.556 × 10^6^, 0.6675 × 10^6^, and 0.7787 × 10^6^ m^3^/day for scenarios 1, 2, 3, and 4, correspondingly. Figure 10 displays the seawater distribution in the horizontal direction in the Moghra aquifer for scenario No. 1 through 100 years of the cultivation project. Abstraction of groundwater water from the 445 wells in the first stage of the cultivation project led to a decline in the groundwater level, and the seawater intruded further inland into the Moghra aquifer compared with the steady state condition for the various tested abstraction schemes. For 1000 m^3^/day/well, the salinity concentration in the development agricultural area in the Moghra region ranged from 2315 mg/l in the eastern parts and increased towards the central and western parts to reach 2450 mg/l; high salinity of about 5063 mg/L was observed in the northern middle area. Salinity concentrations in the Moghra region’s development agricultural area for 1250 m^3^/day/well ranged from 2323 mg/L in the eastern portions to 2543 mg/L in the central and western regions, with the northern middle area exhibiting high salinity at roughly 5330 mg/L. For the 1500 m^3^/day/well the development agricultural area in the Moghra region had a salinity concentration of 2337 mg/l in the eastern portions, rising to 2550 mg/l in the center and western regions, and a high salinity of approximately 5597 mg/L in the northern middle area. According to the scenario of 1750 m^3^/day/well, the salinity concentration in the Moghra region’s development agricultural area ranged from 2352 mg/l in the eastern parts to 2602 mg/l in the central and western parts, with the northern middle area exhibiting high salinity of roughly 6231 mg/L.

**Fig. 11:**
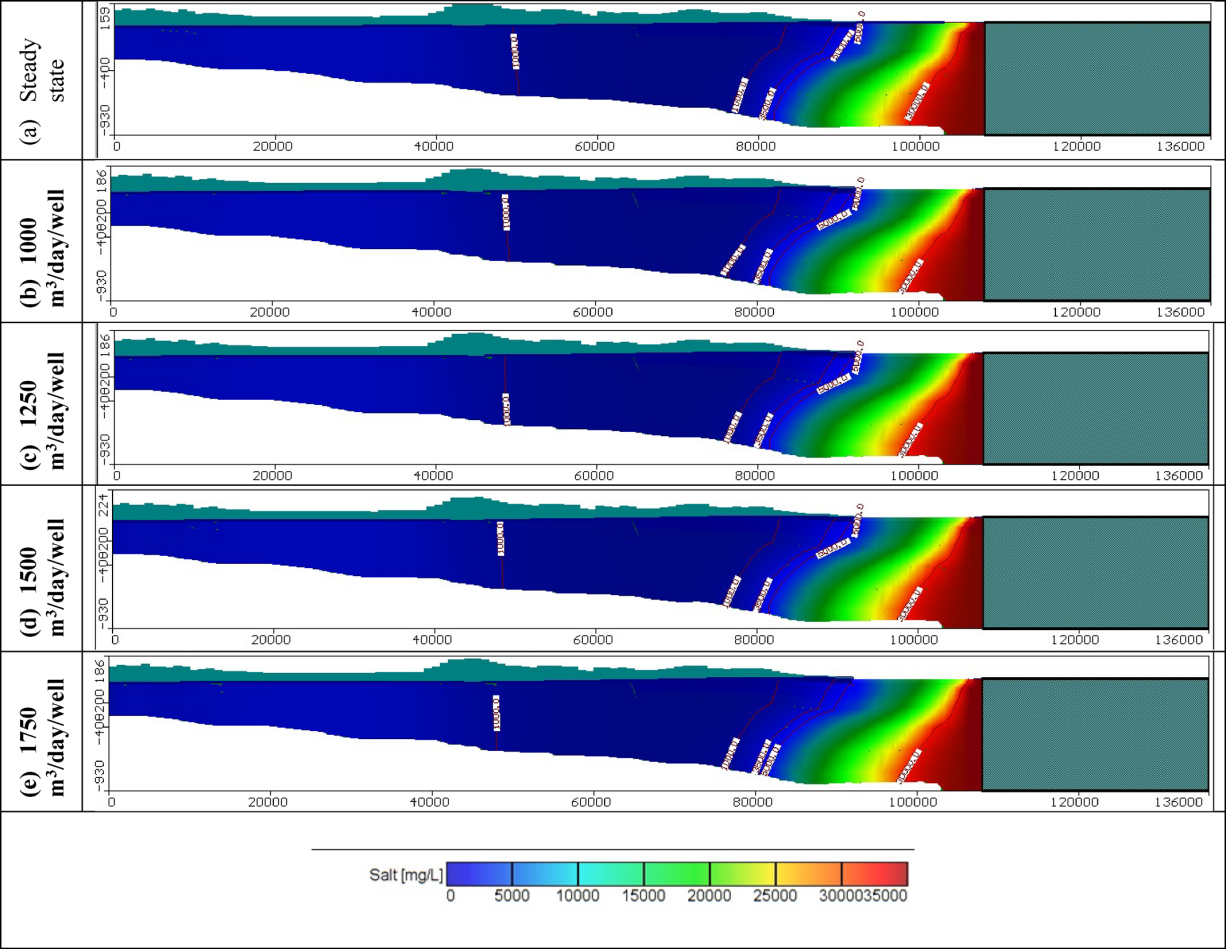
Seawater distribution in vertical cross-section No. 1 in the Moghra aquifer for Scenarios 1 to 4 (445 wells): (**a**) Steady state, (**b**) 1000 m^3^/day/well, (**c**) 1250 m^3^/day/well, (**d**) 1500 m^3^/day/well, and (**e**) 1750 m^3^/day/well.

**Fig. 12:**
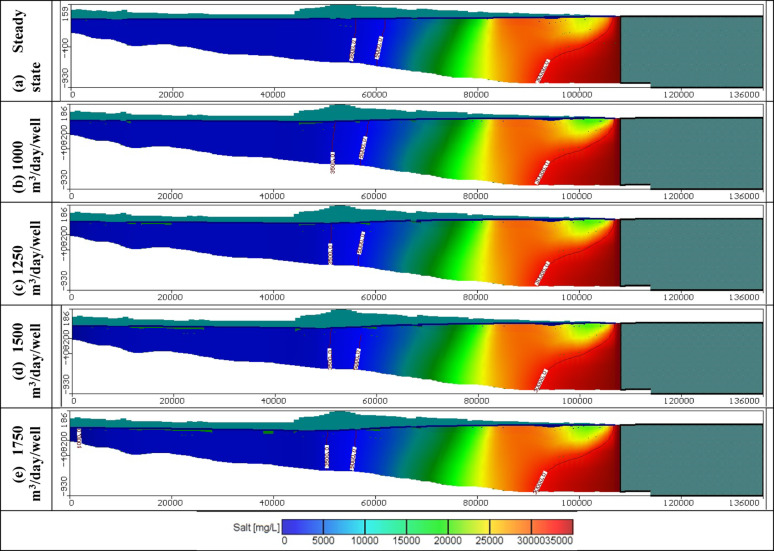
Seawater distribution in vertical cross-section No. 2 in the Moghra aquifer for Scenarios 1 to 4 (445 wells): (**a**) Steady state, (**b**) 1000 m^3^/day/well, (**c**) 1250 m^3^/day/well, (**d**) 1500 m^3^/day/well, and (**e**) 1750 m^3^/day/well.

**Fig. 13:**
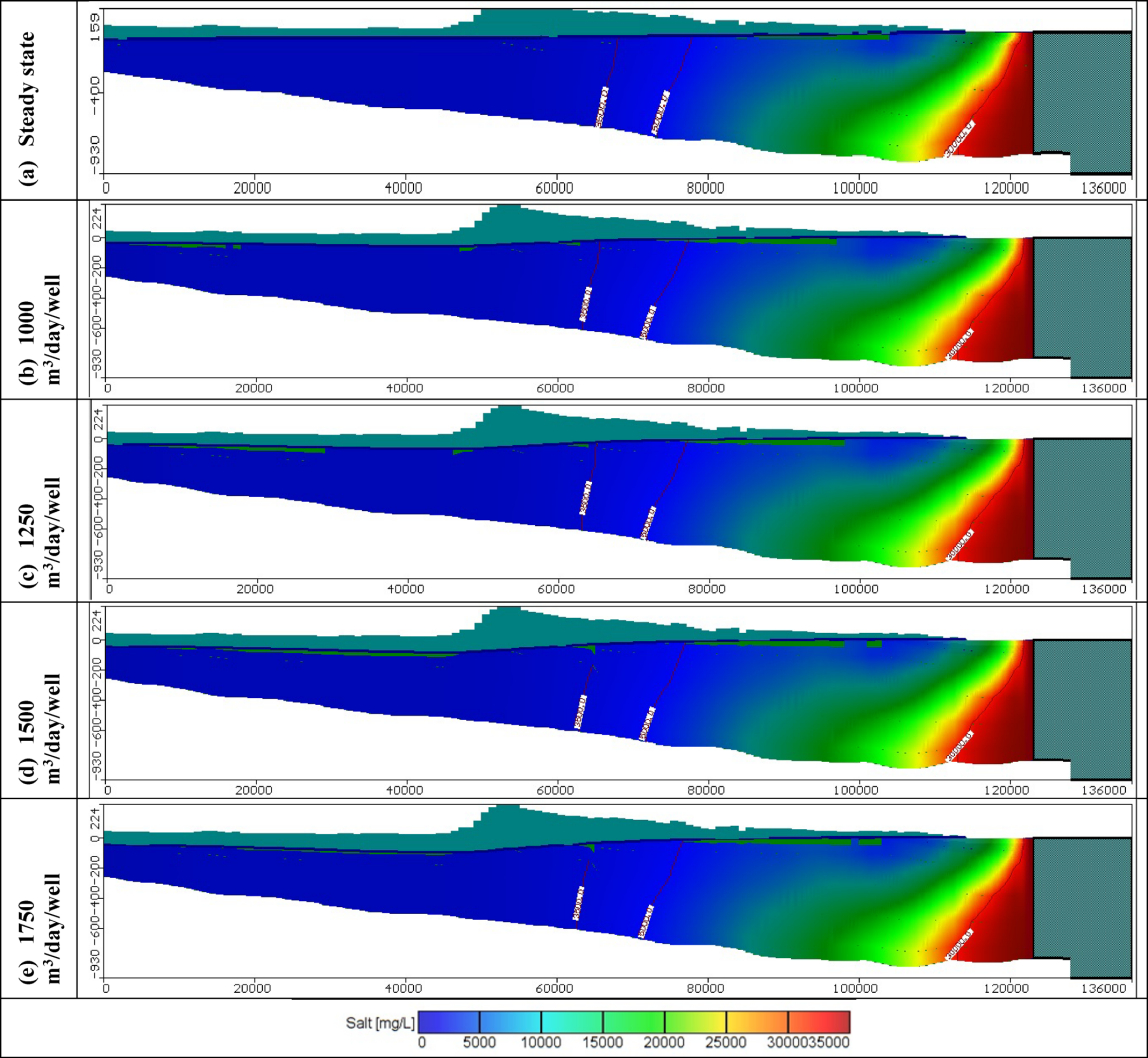
Seawater distribution in vertical cross-section No. 3 in the Moghra aquifer for Scenarios 1 to 4 (445 wells): (**a**) Steady state, (**b**) 1000 m^3^/day/well, (**c**) 1250 m^3^/day/well, (**d**) 1500 m^3^/day/well, and (**e**) 1750 m^3^/day/well.

Figures 11, 12, and 13 show the distribution of seawater in the Moghra aquifer in cross-section "Introduction", "Methodology", and "Study Area", located in the eastern, central and western parts, respectively, for scenarios No. 1, 2, 3, and 4. The 445 wells had abstraction rates of 1000, 1250, 1500, and 1750 m^3^/day/well, respectively. Table 3 summarized the intruded length in cross-section "Introduction", "Methodology", and "Study Area" for abstraction scenarios of 1, 2, 3, and 4. The concentration contour lines of saline water for 3500, 5000, and 30,000 mg/L were further advanced inland into the Moghra aquifer with distances of 0.8, 0.55, 0.4, and 0.1 km upon comparison with the base scenario case, when the rate of abstraction was increased to 1000 m^3^/day/well (Scenario No. 1). Increasing the rate of abstraction to 1250 m^3^/day/well (Scenario No. 2) led to an intrusion of the concentration contour lines of saline water for 3500, 5000, and 3000 mg/L inland more in the central of the Moghra aquifer to reach distances of 56.9, 51.3, and 16 km and advance more into the Moghra aquifer with distances of 4.2, 3.4, and 0.25 km compared with the base scenario case. The concentration contour lines of saline water for 3500, 5000, and 3000 mg/L intruded further inland in the western Moghra region to reach distances of 60.4, 51.5, and 10 km when the rate of abstraction was increased to 1750 m^3^/day/well (Scenario No. 4). In comparison to the base case, the saline water advanced further into the Moghra aquifer to reach distances of 3.3, 1.7, and 0.0 km.

**Table 3:** Seawater intrusion penetration length (km) compared with the base case in the Moghra aquifer for Different abstraction rates for scenarios 1, 2, 3, and 4 (Total number of wells: 445):

Cross-section	Contour line (mg/L)	Base case SWI length	Scenario no. 1	Scenario no. 2	Scenario no. 3	Scenario no. 4
			Total abstraction 0.445 Mm^3^/day	Total abstraction 0.556 Mm^3^/day	Total abstraction 0.667 Mm^3^/day	Total abstraction 0.778 Mm^3^/day
No. 1	1000	30.70	31.5	31.5	31.5	31.5
	3500	27.20	27.75	27.75	27.75	27.75
	5000	26.00	26.4	26.4	26.4	26.4
	30,000	9.20	9.3	9.3	9.3	9.3
No. 2	3500	53.3	56.9	56.9	57.2	57.5
	5000	47.9	51.3	51.3	52	52.5
	30,000	15.75	16.0	16.0	16.1	16.15
No. 3	3500	56.85	59.75	60.0	60.1	60.4
	5000	49.8	50.2	51.25	51.3	51.5
	30,000	10.0	10	10	10	10

#### Impact of groundwater exploitation in saltwater intrusion in the Moghra aquifer (scenarios no. 5, 6, 7 and 8)

**Fig. 14:**
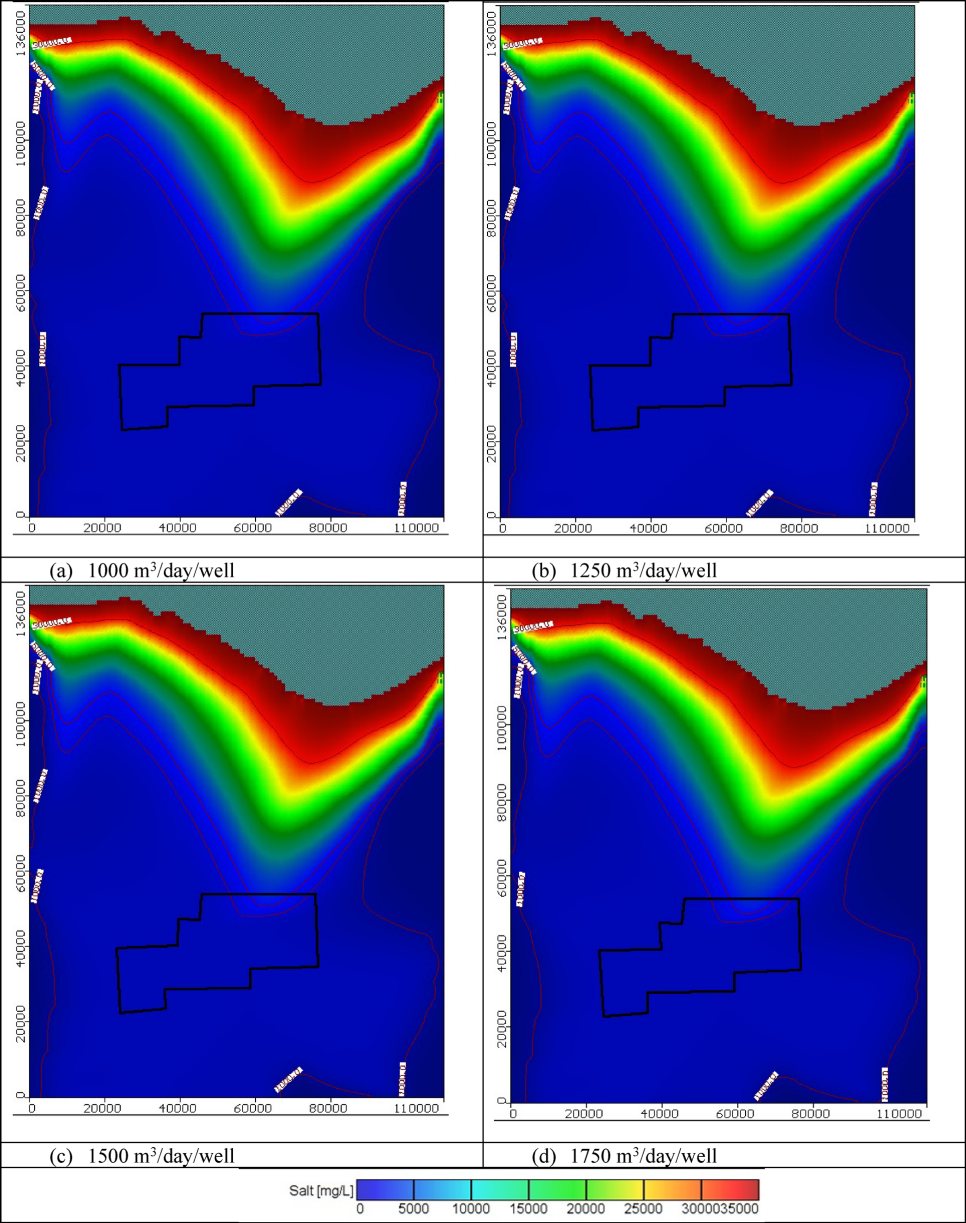
Seawater distribution in the Moghra aquifer for Scenarios 5 to 8 (1000 wells): (**a**) 1000 m^3^/day/well, (**b**) 1250 m^3^/day/well, (**c**) 1500 m^3^/day/well, and (**d**) 1750 m^3^/day/well.

In these cases, there are 1000 wells instead of 445, and tests were again conducted with four different abstraction rates: 1000, 1250, 1500, and 1750 m^3^/day/well. For scenarios 5, 6, 7, and 8, the overall abstraction rate is 1.00 × 10^6^, 1.25 × 10^6^, 1.50 × 10^6^, and 1.75 × 10^6^ m^3^/day, respectively. The horizontal distribution of seawater in the Moghra aquifer for scenarios No. 5, 6, 7, and 8 over the course of the agriculture project’s 100 years is depicted in Fig. 14. In comparison to the steady state condition for the different abstraction strategies studied, the groundwater level decreased, and seawater intruded more inland into the Moghra aquifer as a result of the extraction of groundwater water from the 1000 wells in the first stage of the agriculture project. The salinity concentration in the Moghra region’s development agricultural area for 1000 m^3^/day/well ranged from 2327 mg/L in the eastern sections to 2566 mg/L in the center and western regions, with the northern middle area showing the highest salinity at roughly 6304 mg/L. The development agricultural area in the Moghra region had a salt concentration of 2381 mg/l in the eastern sections, 2585 mg/l in the central and western parts, and a high salinity of 6415 mg/L in the northern middle area for the abstraction rate of 1250 m^3^/day/well. Salinity concentrations in the Moghra region’s development agricultural area range from 2337 mg/L in the eastern parts to 2550 mg/L in the central and western parts, with a high salinity of roughly 6734 mg/L in the northern middle area, for a scenario of 1500 m^3^/day/well. For 1750 m^3^/day/well, the salinity concentration in the Moghra region’s development agricultural area ranged from 2390 mg/l in the eastern sections to 3133 mg/l in the central and western portions. The northern middle area showed high salinity of almost 7360 mg/L. The distribution of seawater in the Moghra aquifer in cross-sections "Introduction", "Methodology", and "Study Area" which are situated in the eastern, central, and western regions, respectively, for scenarios No. 5, 6, 7, and 8 are depicted in Figs. A.6, A.7, and A.8. Abstraction rates for the 1000 wells were 1000, 1250, 1500, and 1750 m^3^/day/well, in that order. Table 4 summarizes the intrusion lengths for the same scenarios. In cross-section No. 1, the rate of abstraction was at 1000 m^3^/day/well in (Scenario No. 5), which resulted in a greater advance of SWI into the Moghra aquifer with distances of 0.1, 0.05, and 0.05 km in comparison to the base case. In Scenario No. 6, the SWI also advanced further into the Moghra aquifer, reaching distances of 0.3, 0.2, and 0.1 km. Increasing the abstraction rate to 1750 m^3^/day/well (Scenario No. 8) caused the concentration contour lines of saline water for 3500, 5000, and 30,000 mg/L to intrude more inland in the central Moghra region, reaching distances of 58.3, 54.75, and 16.25 km. It also spread further into the Moghra aquifer, reaching distances of 5.6, 6.65, and 0.3 km. In cross-section No. 3, the SWI was advanced further into the Moghra aquifer with distances of 4.4, 2.1, and 0.0 km in comparison to the base case when the rate of abstraction was increased to 1750 m^3^/day/well (Scenario No. 8).

**Table 4:** Seawater intrusion penetration length (km) compared with the base case in the Moghra aquifer for different abstraction rates for scenarios 5, 6, 7, and 8 (Total number of wells: 1000).

Cross-section	Contour line (mg/L)	Base case SWI length	Scenario no. 5	Scenario no. 6	Scenario no. 7	Scenario no. 8
			Total abstraction 1.00 Mm^3^/day	Total abstraction 1.25 Mm^3^/day	Total abstraction 1.50 Mm^3^/day	Total abstraction 1.75 Mm^3^/day
No. 1	1000	30.70	31	31.3	31.3	31.3
	3500	27.20	27.3	27.5	27.6	27.7
	5000	26.00	26.05	26.2	26.3	26.4
	30,000	9.20	9.25	9.3	9.33	9.4
No. 2	3500	53.3	54.05	58	58	58.3
	5000	47.9	48.6	53.6	54.05	54.75
	30,000	15.75	15.75	16	16.2	16.25
No. 3	3500	56.85	57.5	61	61.2	61.5
	5000	49.8	50	51.6	51.75	51.9
	30,000	10.0	10	10	10	10

### Mitigation of seawater intrusion in Moghra aquifer

#### Impact of abstraction of saline water to control saltwater intrusion in the Moghra aquifer

**Fig. 15:**
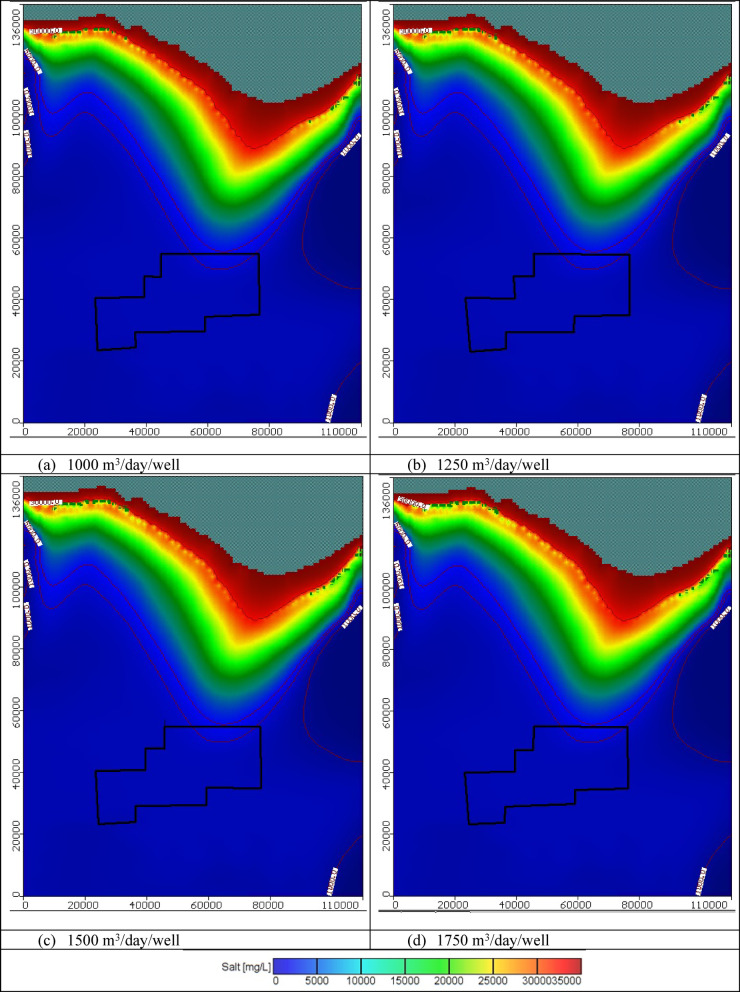
Seawater distribution in the Moghra aquifer after using the abstraction of saline water as a controlling method, Scenarios 9 to 12: (**a**) 1000 m^3^/day/well, (**b**) 1250 m^3^/day/well, (**c**) 1500 m^3^/day/well, and (**d**) 1750 m^3^/day/well.

There are 445 wells in all in these scenarios for agricultural management, and four different saline water abstraction rates of 1000, 1250, 1500, and 1750 m^3^/day/well were investigated to manage seawater intrusion into the Moghra aquifer. For scenarios 9, 10, 11, and 12, one hundred abstracted saline water wells were installed and placed in the contour line of concentration equal to 30,000 mg/L. The wells’ different abstraction rates were 1000, 1250, 1500, and 1750 m^3^/day/well, respectively. For scenarios 9, 10, 11, and 12, the total amount of saline water abstracted is thus 0.1 × 10^6^, 0.125 × 10^6^, 0.15 × 10^6^, and 0.175 × 10^6^ m^3^/day, respectively. The horizontal distribution of seawater in the Moghra aquifer for scenarios No. 9, 10, 11, and 12 over the course of the agriculture project’s 100 years is depicted in Fig. 15. In comparison to the steady state condition for the different evaluated abstraction saline water schemes, the Moghra aquifer’s backwater towards the Mediterranean Sea was attenuated when salty water was extracted from the 100 wells. In the Moghra region’s development agricultural area, the salinity concentration for 1000 m^3^/day/well ranged from 2325 mg/l in the eastern sections to 2566 mg/l in the center and western portions. The northern middle area showed the highest salinity, at roughly 5314 mg/L. For 1250 m^3^/day/well, the salinity concentration in the development agricultural area in the Moghra region ranged from 2320 mg/l in the eastern parts, increased towards the central and western parts to reach 2553 mg/l, and high salinity was observed in the northern middle area about 5132 mg/L. Salinity concentrations in the Moghra region’s development agricultural area for 1500 m^3^/day/well range from 2313 mg/L in the eastern portions to 2543 mg/L in the central and western regions, with the northern middle area exhibiting high salinity at roughly 5054 mg/L. According to the scenario of 1750 m^3^/day/well, the salinity concentration in the Moghra region’s development agricultural area ranged from 2310 mg/l in the eastern parts to 2505 mg/l in the central and western parts, with the northern middle area exhibiting high salinity of roughly 5003 mg/L. Figures A.9, A.10, and A.11 show the distribution of seawater in the Moghra aquifer for abstraction of saline water scenarios No. 9, 10, 11, and 12 in cross-sections "Introduction", "Methodology", and "Study Area", which can be found in the eastern, central, and western regions, respectively. In addition, Table 5 summarizes the saltwater intrusion lengths for the same scenarios. Furthermore, the saltwater intrusion lengths for the same scenarios are summarized in Table 6 In cross-section No. 1, the SWI contour line 30,000 mg/L attenuated back towards the sea with distances of − 0.7, − 0.8, − 1.0 and − 1.05 km compared with the base case. In Scenarios 13, 14, 15, and 16, respectively, the SWI contour line of 30,000 mg/L attenuated back towards the sea with distances of − 1.0, − 1.3, − 1.8, − 2.7 km through comparing with the base case scenario in cross-section No. 2. In addition, the SWI contour line 30,000 mg/L retreated back towards the sea with distances of − 1.95, − 2.0, − 2.1, and − 2.2 km in comparison to the base scenario in cross-section No. 3.

**Table 5:** Seawater intrusion penetration length (km) compared with the base case in the Moghra aquifer for abstraction of saline water scenarios 9, 10, 11, and 12 (Total number of wells: 445) for SWI control:

Cross-section	Contour line (mg/L)	Base case SWI length	Scenario no. 9	Scenario no. 10	Scenario no. 11	Scenario no. 12
			Total abstraction 1000 m^3^/day/well	Total abstraction 1250 m^3^/day/well	Total abstraction 1500 m^3^/day/well	Total abstraction 1750 m^3^/day/well
No. 1	3500	27.20	28.1	28.1	28.05	28.05
	5000	26.00	26.9	26.9	26.8	26.8
	30,000	9.20	8.6	8.5	8.3	8.25
No. 2	3500	53.3	57	57	57	57
	5000	47.9	51.5	51.5	51.5	51.5
	30,000	15.75	14.9	14.6	14.1	13.2
No. 3	3500	56.85	60.6	60.6	60.6	60.6
	5000	49.8	52	52	52	52
	30,000	10.0	8.05	8.0	7.9	7.8

**Table 6:** Seawater intrusion penetration length (km) compared with the base case in the Moghra aquifer for artificial recharge scenarios 13, 14, 15, and 16 (Total number of wells: 445) for SWI control:

Cross-section	Contour line (mg/L)	Base case SWI length	Scenario no. 13	Scenario no. 14	Scenario no. 15	Scenario no. 16
			Total abstraction 1000 m^3^/day/well	Total abstraction 1250 m^3^/day/well	Total abstraction 1500 m^3^/day/well	Total abstraction 1750 m^3^/day/well
No. 1	3500	27.20	27.05	27	26.9	26.9
	5000	26.00	26.5	26.3	26.2	26.1
	30,000	9.20	9.15	9.15	9.15	9.15
No. 2	3500	53.3	50.1	50	50	50
	5000	47.9	49.5	49.5	49.5	49.5
	30,000	15.75	15.8	15.75	15.75	15.75
No. 3	3500	56.85	60.9	61	61	61
	5000	49.8	49.9	48.5	48.15	48.15
	30,000	10.0	9.05	9.1	9	9

#### Impact of artificial recharge to control seawater intrusion in the Moghra aquifer

**Fig. 16:**
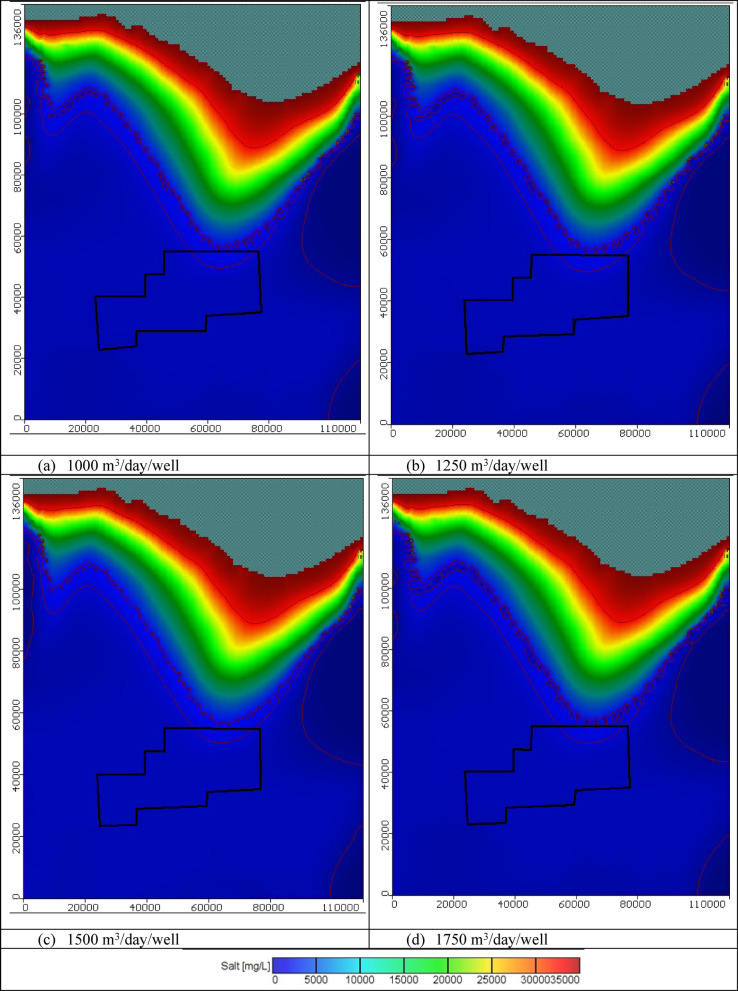
Seawater distribution in the Moghra aquifer after using artificial recharge as a controlling method, Scenarios 13 to 16: (**a**) 1000 m^3^/day/well, (**b**) 1250 m^3^/day/well, (**c**) 1500 m^3^/day/well, and (**d**) 1750 m^3^/day/well.

In these scenarios, four various artificial recharge rates of freshwater equal to 1000, 1250, 1500, and 1750 m^3^/day/well were tested to control saltwater intrusion in the Moghra aquifer. One hundred artificial recharge water wells were implemented and positioned in the contour line of concentration equal to 5,000 mg/L with various recharge rates on wells equal to 1000, 1250, 1500, and 1750 m^3^/day/well for scenarios 13, 14, 15, and 16, consequently. The total volumes of artificial recharge rates thus equal 0.1 × 10^6^, 0.125 × 10^6^, 0.15 × 10^6^, and 0.175 × 10^6^ m^3^/day for scenarios 13, 14, 15, and 16, respectively. Figure 16 displays the seawater distribution in the horizontal direction in the Moghra aquifer for scenarios No. 13, 14, 15, and 16 over 100 years of the cultivation project. Artificial recharge through 100 wells led to attenuated back seawater towards the Mediterranean Sea in the Moghra aquifer compared with the steady state condition for the various tested artificial freshwater schemes. For 1000 m^3^/day/well, the salinity concentration in the development agricultural area in the Moghra region ranged from 2319 mg/l in the eastern parts, increased towards the central and western parts to reach 2529 mg/l, and high salinity was observed in the northern middle area about 5071 mg/L. The agricultural development area in the Moghra region has a salt concentration of 2308 mg/l in the eastern sections, 2523 mg/l in the central and western portions, and high salinity of 4973 mg/L in the northern middle area for 1250 m^3^/day/well. In the Moghra region’s development agricultural area, the salinity concentration for 1500 m^3^/day/well ranged from 2293 mg/l in the eastern sections to 2510 mg/l in the center and western portions. The northern middle area showed the highest salinity, at roughly 4867 mg/L. For 1750 m^3^/day/well, the development agricultural area in the Moghra region has a salt concentration of 2261 mg/l in the eastern portions, rising to 2494 mg/l in the center and western regions, and a high salinity of 4755 mg/L in the northern middle area. In cross-sections "Introduction", "Methodology", and "Study Area", which are situated in the eastern, central, and western regions, respectively, the distribution of seawater in the Moghra aquifer for artificial recharge of freshwater scenarios No. 13, 14, 15, and 16 is depicted in Figs. A.12, A.13, and A.14. For scenario No. 15, in cross-section No. 3, the artificial recharge at a rate of 1500 m^3^/day/well resulted in attenuating the contour lines of seawater concentrations of 3500, 5000, and 30,000 mg/L towards the Mediterranean Sea, reaching distances of 61, 48.5, and 9.1 km, correspondingly. Furthermore, compared to the base case, the seawater contour lines of concentrations 5000 and 30,000 mg/L in the Moghra aquifer receded towards the shore by − 1.7 and − 0.95 km, respectively. In scenario 16, in cross-section No. 1, with comparison to the base scenario case, the seawater contour lines of concentration 3500, 5000, and 30,000 mg/L in the Moghra aquifer retreated towards the shore by distances of − 0.85, − 0.3, and − 0.15 km, respectively. These distances equal − 6.3, − 1.3, and − 0.15 km, respectively. In cross-section "Methodology", the seawater contour lines at concentrations of 5000 and 30,000 mg/L in the Moghra aquifer receded towards the sea by − 2.05 and − 0.95 km, respectively, compared to the base case (Scenario 16).

#### Impact of combination between groundwater abstraction and artificial recharge

**Fig. 17:**
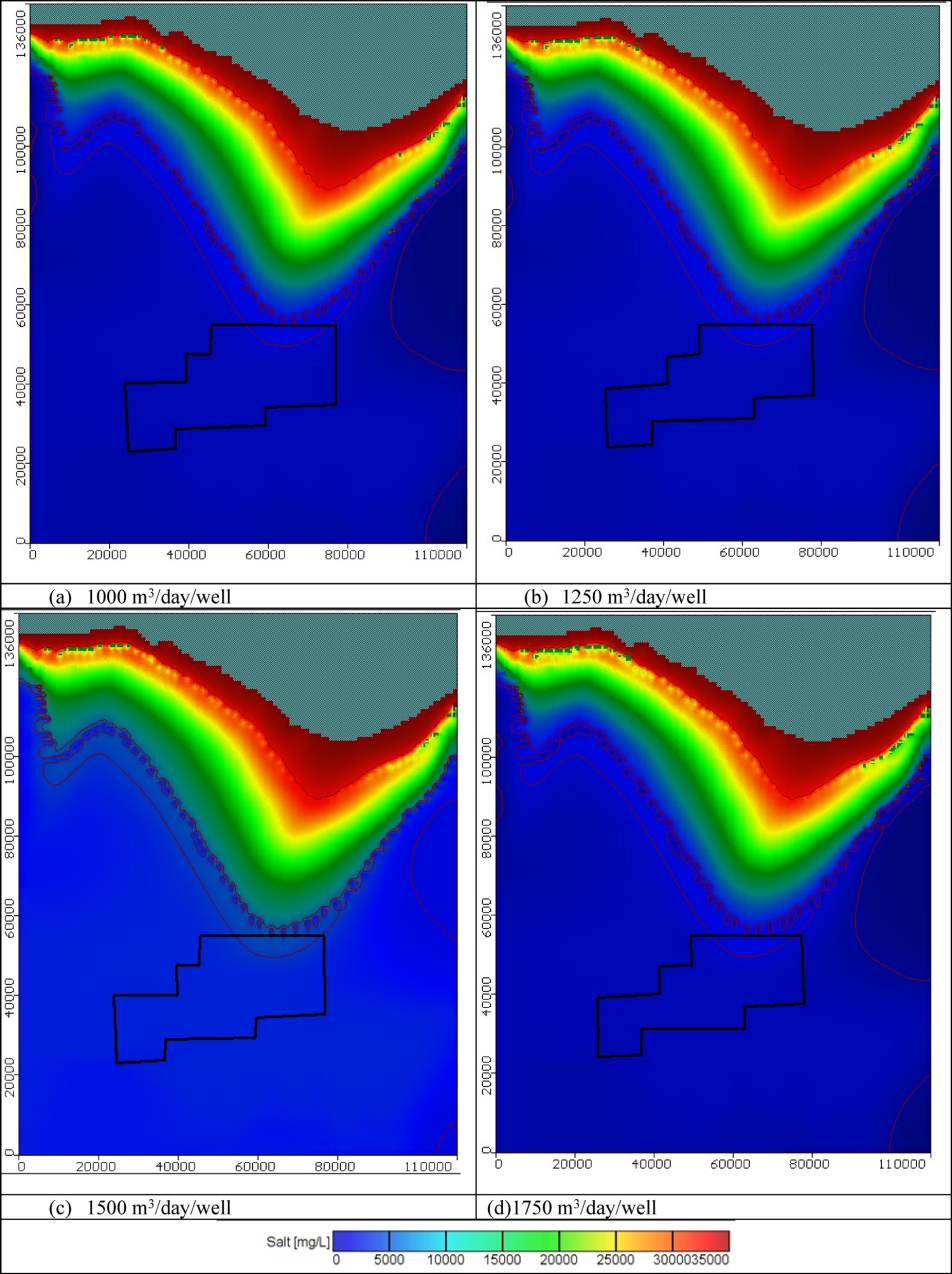
Seawater distribution in the Moghra aquifer after using a combination of saline water abstraction and artificial recharge as a controlling method, Scenarios 17 to 20: (**a**) 1000 m^3^/day/well, (**b**) 1250 m^3^/day/well, (**c**) 1500 m^3^/day/well, and (**d**) 1750 m^3^/day/well.

**Fig. 18:**
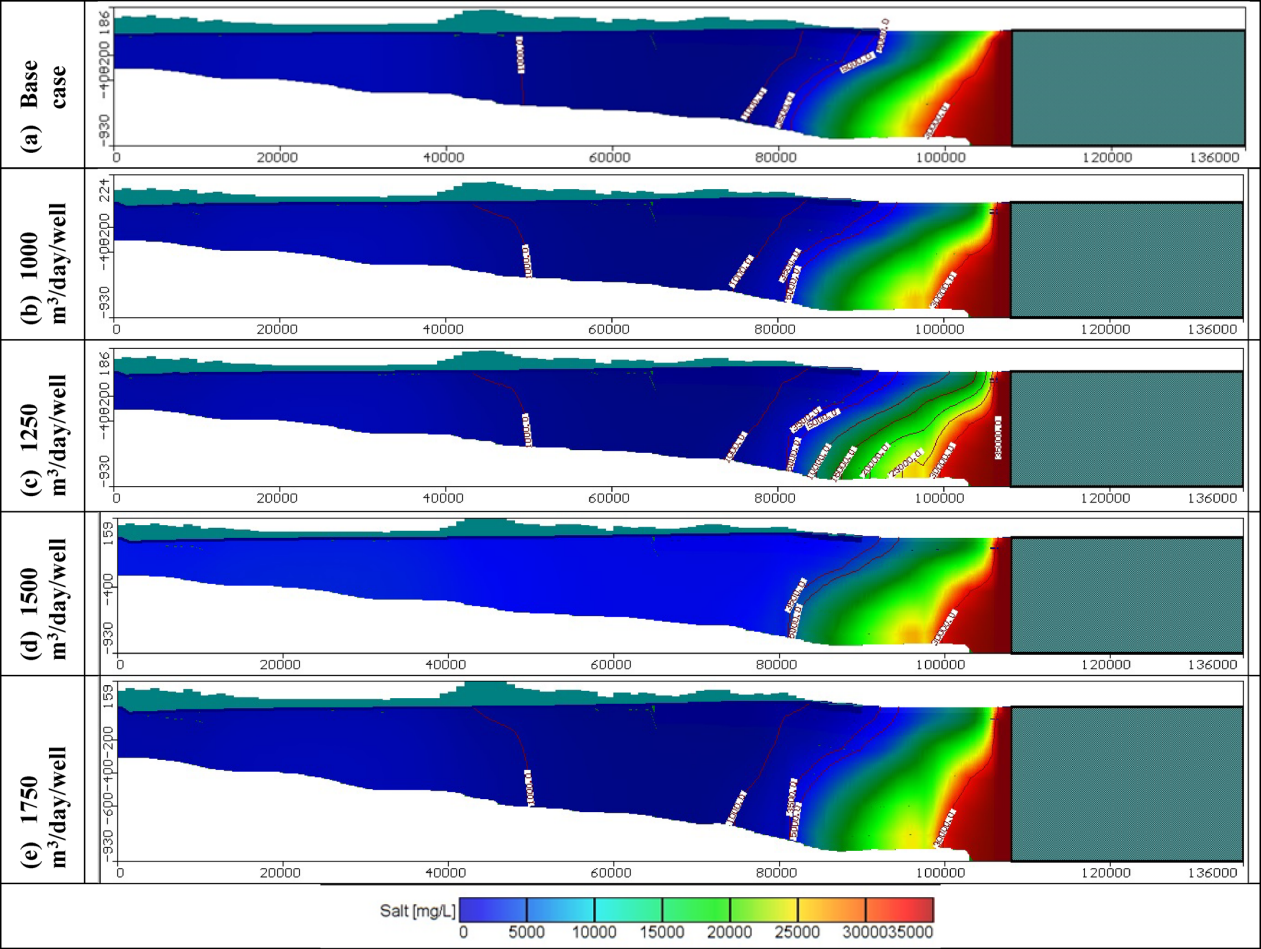
Seawater distribution in vertical cross-section No. 1 in the Moghra aquifer for a combination of saline water abstraction and artificial recharge controlling scenarios 17 to 20: (**a**) Base Case, (**b**) 1000 m^3^/day/well, (**c**) 1250 m^3^/day/well, (**d**) 1500 m^3^/day/well, and (**e**) 1750 m^3^/day/well.

**Fig. 19:**
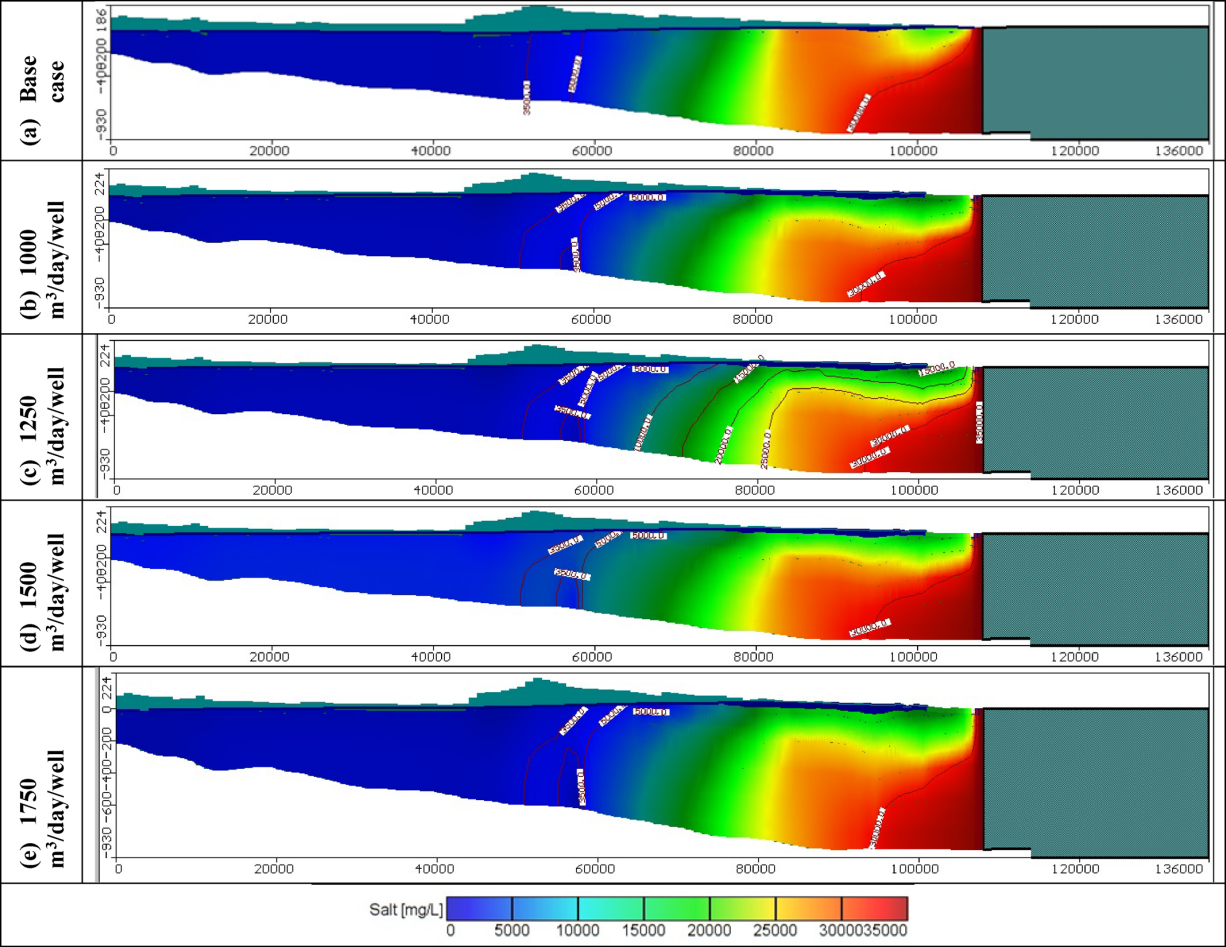
Seawater distribution in vertical cross-section No. 2 in the Moghra aquifer for a combination between saline water abstraction and artificial recharge controlling scenarios 17 to 20: (**a**) Base Case, (**b**) 1000 m^3^/day/well, (**c**) 1250 m^3^/day/well, (**d**) 1500 m^3^/day/well, and (**e**) 1750 m^3^/day/well.

**Fig. 20:**
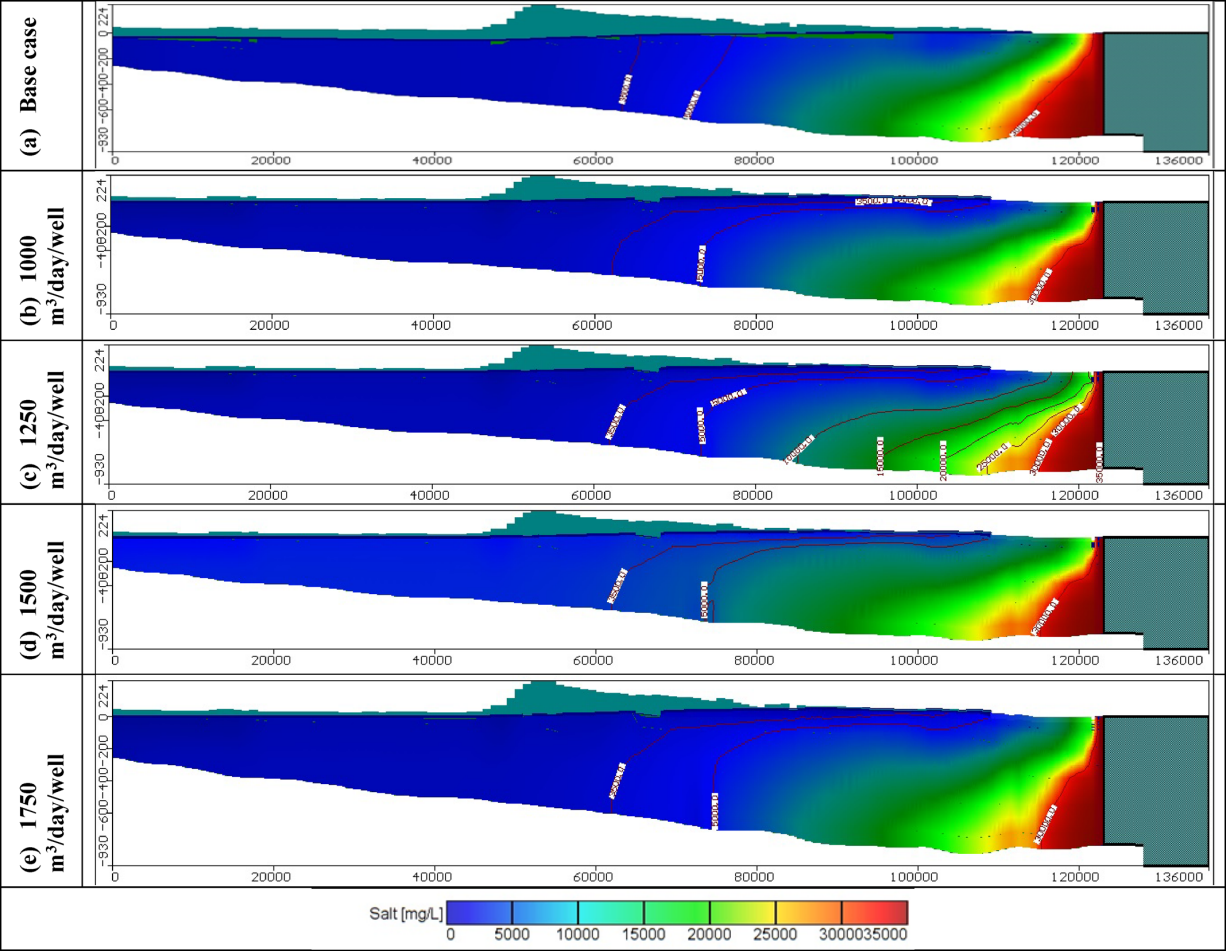
Seawater distribution in vertical cross-section No. 3 in the Moghra aquifer for a combination between saline water abstraction and artificial recharge controlling scenarios 17 to 20: (**a**) Base Case, (**b**) 1000 m^3^/day/well, (**c**) 1250 m^3^/day/well, (**d**) 1500 m^3^/day/well, and (**e**) 1750 m^3^/day/well.

In these scenarios, four various abstraction rates of saline water equal to 1000, 1250, 1500, and 1750 m^3^/day/well combined with four different scenarios of artificial recharge were tested to control saltwater intrusion in the Moghra aquifer. One hundred saline water abstraction wells were implemented and positioned in the contour line of concentration equal to 30,000 mg/L combined with hundred artificial recharge rates through wells located on the contour line of 5,000 mg/L, with various abstraction and recharge rates equal to 1000, 1250, 1500, and 1750 m^3^/day/well for scenarios 17, 18, 19, and 20, consequently. The total volume of abstracted saline water thus equals 0.1 × 10^6^, 0.125 × 10^6^, 0.15 × 10^6^, and 0.175 × 10^6^ m^3^/day for scenarios 17, 18, 19, and 20, respectively. In addition, The total volume of recharged freshwater equals 0.1 × 10^6^, 0.125 × 10^6^, 0.15 × 10^6^, and 0.175 × 10^6^ m^3^/day for scenarios 17, 18, 19, and 20, respectively. Figure 17 shows the distribution of seawater in the horizontal direction in the Moghra aquifer for scenarios No. 17, 18, 19, and 20 over 100 years of the cultivation project. Abstraction of saline water from the 100 wells combined with artificial recharge of 100 wells, led to attenuated back seawater towards the Mediterranean Sea in the Moghra aquifer compared with the steady state condition for the various tested abstraction saline water schemes and compared with abstraction and artificial recharge only. For 1000 m^3^/day/well, the salinity concentration in the developing agricultural area in the Moghra region ranged from 2293 mg/l in the eastern sections to 2540 mg/l in the center and western regions, with high salinity recorded in the northern middle area about 4937 mg/L. For 1250 m^3^/day/well, the salinity concentration in the Moghra region’s development agricultural area ranged from 2293 mg/l in the eastern sections to 2536 mg/l in the central and western portions, with a high salinity of 4818 mg/L in the northern middle. For 1500 m^3^/day/well, the salinity concentration in the developing agricultural area in the Moghra region ranged from 2243 mg/l in the eastern sections to 2510 mg/l in the center and western portions, with a high salinity of 4692 mg/L in the northern middle area. For 1750 m^3^/day/well, the salinity concentration in the development agricultural area in the Moghra region ranged from 2243 mg/l in the eastern parts, increased towards the central and western parts to reach 2505 mg/l, and high salinity was observed in the northern middle area about 4562 mg/L. Table 7 summarizes the seawater intrusion lengths for the same scenarios and the seawater intrusion distribution in sections "Introduction", "Methodology", and "Study Area" are showed in Figs. 18, 19, and 20, respectively. In scenario No. 20, saline water abstraction combined with artificial recharge at rates of 1750 m^3^/day/well resulted in attenuation of the contour lines of seawater concentrations of 3500, 5000, and 30,000 mg/L towards the Mediterranean Sea, reaching distances of 26.9, 26.1, and 8.2 km, respectively. Furthermore, the seawater contour lines of concentration 3500, 5000, and 30,000 mg/L in the Moghra aquifer withdrew towards the shore with distances of − 0.85, − 0.2, and − 1.05 km, respectively, when comparing to the base scenario case (cross-section No. 1). In cross-section "Methodology", and scenario No. 20, comparing with to the base scenario case, the seawater contour lines with concentrations of 3500, 5000, and 30,000 mg/L in the Moghra aquifer retreated towards the shore by − 6.3, − 1.3, and − 2.5 km, respectively. In addition, in cross-section "Study Area", the seawater contour lines at concentrations of 5000 and 30,000 mg/L in the Moghra aquifer receded towards the shore by − 2.0 and − 2.4 km, respectively, compared to the base condition.

**Table 7:** Seawater intrusion penetration length (km) compared with the base case in the Moghra aquifer for combination of saltwater extraction and artificial recharge scenarios 17, 18, 19, and 20 (Total number of wells: 445) for SWI control:

Cross-section	Contour line (mg/L)	Base case SWI length	Scenario No. 17	Scenario No. 18	Scenario No. 19	Scenario No. 20
			Total abstraction 1000 m^3^/day/well	Total abstraction 1250 m^3^/day/well	Total abstraction 1500 m^3^/day/well	Total abstraction 1750 m^3^/day/well
No. 1	3500	27.20	27.0	26.9	26.9	26.9
	5000	26.00	26.5	26.3	26.2	26.1
	30,000	9.20	8.6	8.3	8.3	8.2
No. 2	3500	53.3	50.1	50	50	50
	5000	47.9	49.5	49.5	49.5	49.5
	30,000	15.75	14.9	14.6	14.25	13.4
No. 3	3500	56.85	61	61	61	61
	5000	49.8	49.9	49.6	48.3	48.2
	30,000	10.0	8.05	8.0	7.8	7.6

## Discussion

### Variation of zone budget in the Moghra aquifer system

**Fig. 21:**
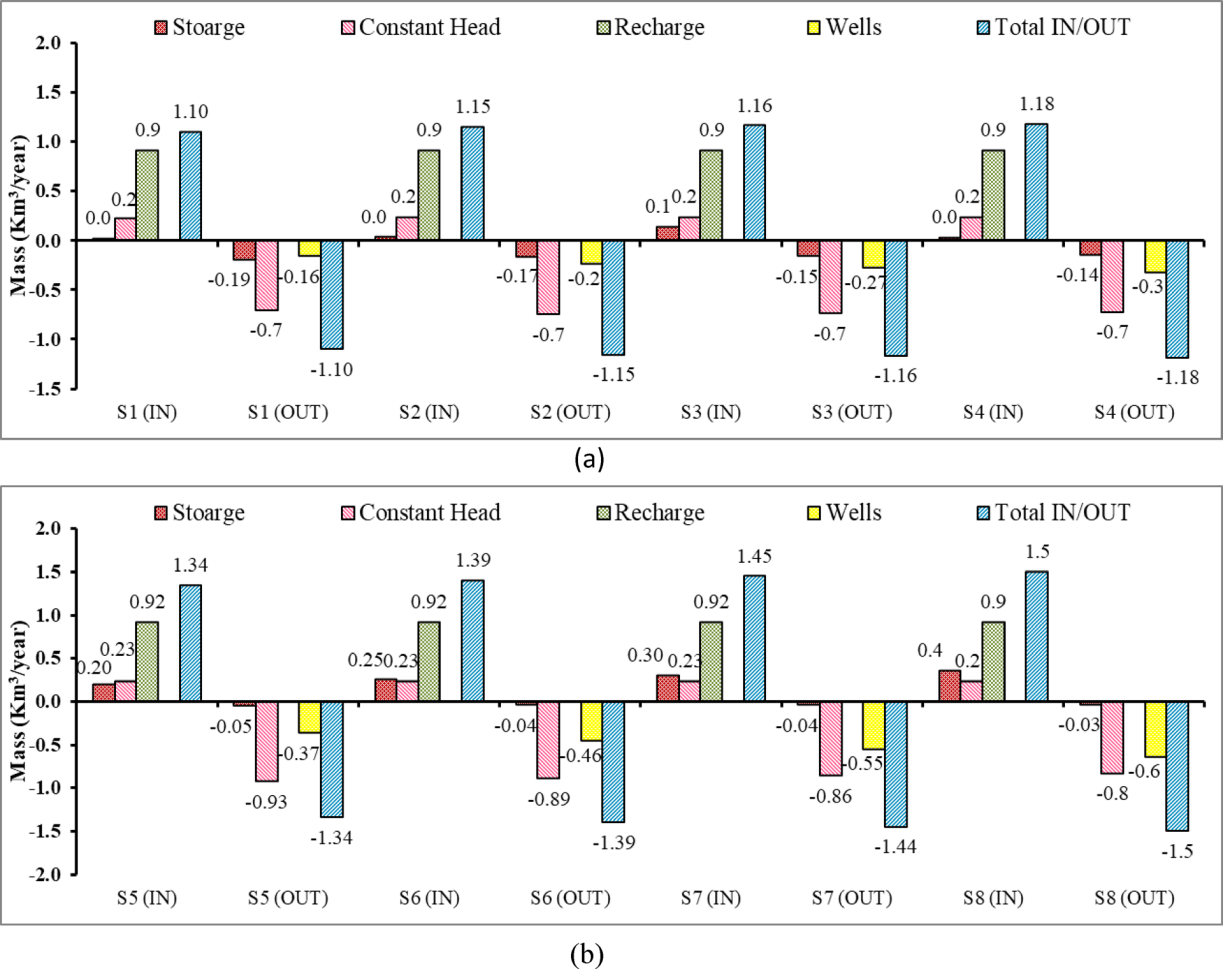
Components of zone budget in the Moghra aquifer for different agricultural management scenarios: (**a**) S1, S2, S3, S4, and (**b**) S5, S6, S7, S8.

Figure 21 presents the variation in the zone budget of flow for the Moghra aquifer system for eight various agricultural management scenarios. The inflow and outflow for each scenario have five various components. These are storage, constant head, recharge, wells, and total flux. Figure 21a and b confirmed that the recharge is the major contributor to the flow budget, followed by the storage, and finally by the flux through the boundary. On the other hand, constant head and wells are the major contributor to the outflow from the Moghra aquifer system. The abstraction through wells increased slightly to reach 0.16, 0.20, 0.24, and 0.28 km^3^/year for S1, S2, S3, and S4 respectively. In addition, the pumping rate rose slightly to be 0.36, 0.46, 0.55, and 0.64 km^3^/year for agricultural management scenarios S5, S6, S7, and S8, respectively. The total budget remained balanced, thus confirming that the Moghra aquifer reached a steady state.

**Fig. 22:**
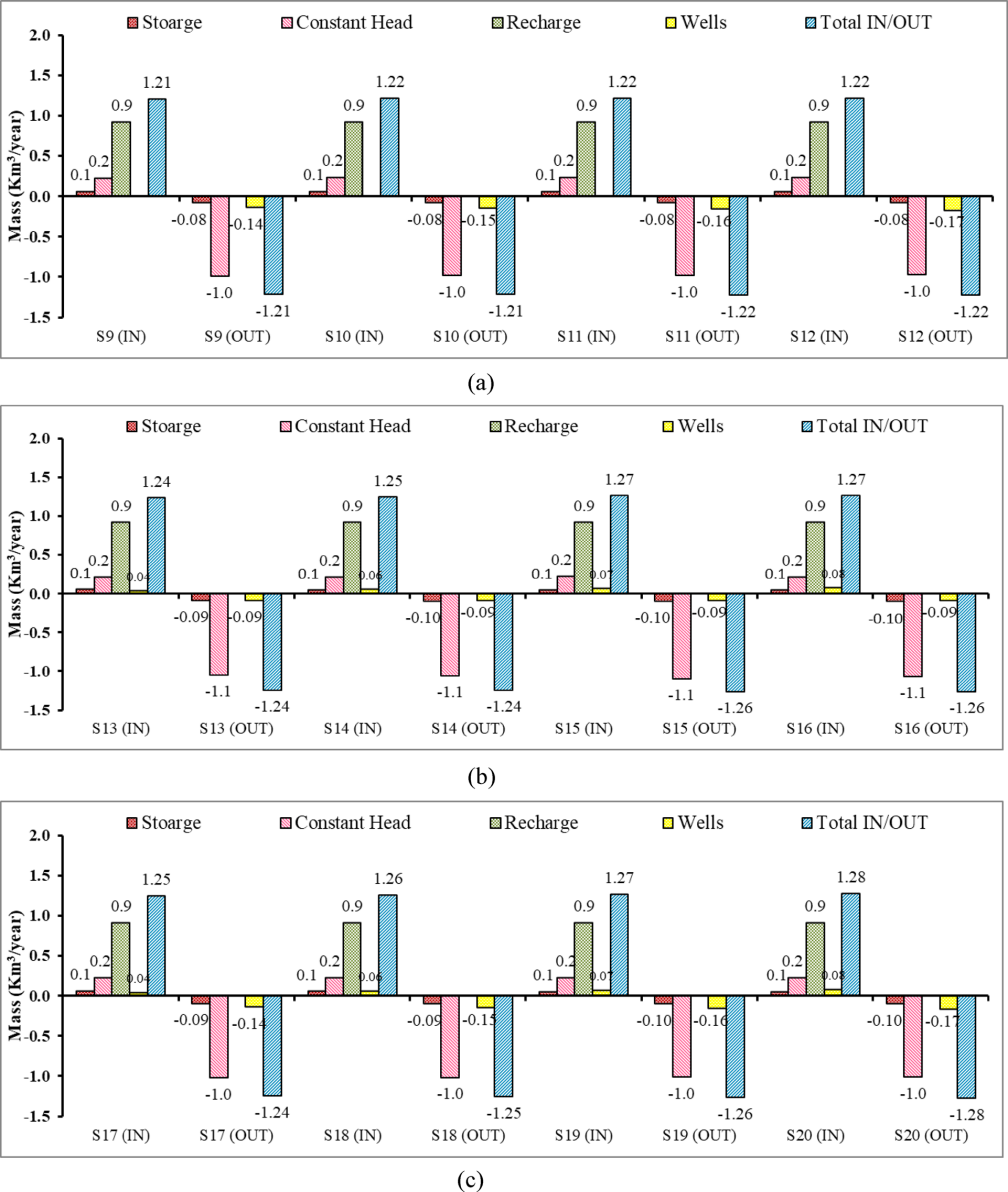
Components of the zone budget in the Moghra aquifer for different scenarios for the mitigation of SWI: (**a**) S9, S10, S11, S12, (**b**) S13, S14, S15, S16, (**c**) S17, S18, S19, S20.

Figure 22 presents the variation in the zone budget of flow for the Moghra aquifer system for twelve different scenarios for mitigation of SWI. Figure 22a, b and c confirmed that the recharge is the major contributor to the flow budget, followed by the constant head, and finally by the storage term. On the other hand, constant head and wells are the major contributors to the outflow from the Moghra aquifer system. The total budget remained balanced, thus confirming that the Moghra aquifer reached a steady state. The total flux into of the Moghra aquifer increased to reach 1.22, 1.27, 1.28 km^3^/year for scenarios: S12, S16, and S20, respectively.

### Mass balance concentration in the Moghra aquifer system

**Fig. 23:**
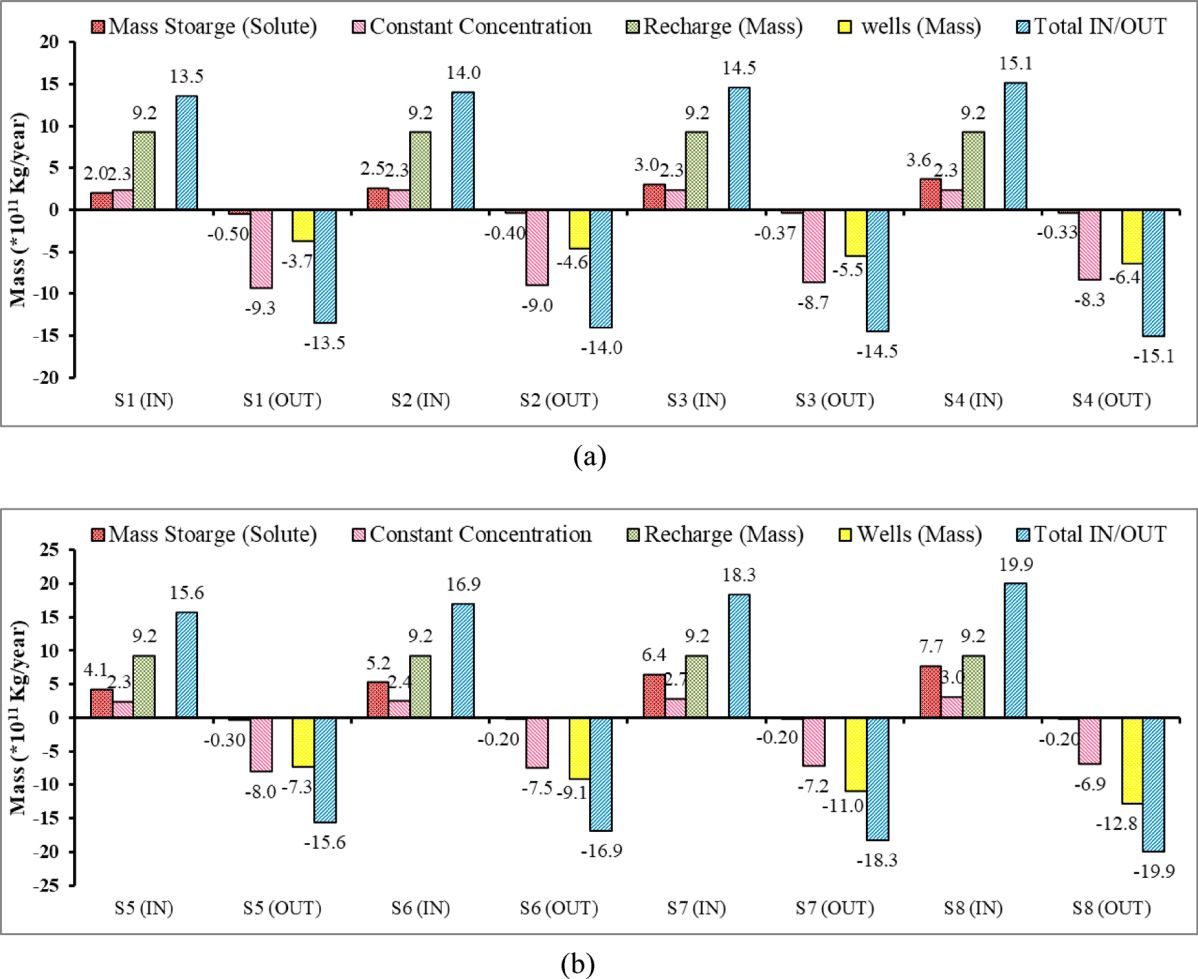
Components of mass balance of the Moghra aquifer for different agricultural management scenarios: (**a**) S1, S2, S3, S4, (**b**) S5, S6, S7, S8.

Figure 23 presents the solute mass balance of the Moghra aquifer across eight various agricultural management scenarios (S1 to S8), with each scenario having inflow (IN) and outflow (OUT) components. The results of this figure show the Moghra aquifer is influenced by heavy groundwater pumping through wells and seawater intrusion, which are reflected in the values of mass flows under different scenarios. The mass storage introduces the variation in the mass stored in the Moghra aquifer, where a positive value means accumulation and a negative value means depletion. The term *constant concentration* indicates the mass entering or leaving the Moghra aquifer due to the fixed concentration zones. The term *recharge* reflects the mass added to the Moghra aquifer through the groundwater recharge through rainfall. The term *wells* presents the mass removed from the Moghra aquifer due to abstraction. Total IN/OUT indicates the total mass flux entering or leaving the aquifer system. Figure 5a indicates that recharge through rainfall is the major contributor by 9.2 × 10^11^ kg/year, followed by the mass through constant concentration ranging from 2 × 1011 to 3.6 × 10^11^ kg/year, and there is a minor increase in storage, followed by a constant value of constant concentration 2.3 × 10^11^ kg/year. The solute mass increased from 4.1 to 7.7 × 10^11^ kg/year, indicating that increasing the groundwater abstraction led to depletion of groundwater level and enhances seawater intrusion for scenarios S5 to S8, respectively. As a result of that, the total mass entering the aquifer system increased to 15.6, 16.9, 18.3, and 19.9 × 10^11^ kg/year for scenarios S5, S6, S7, and S8, respectively.

**Fig. 24:**
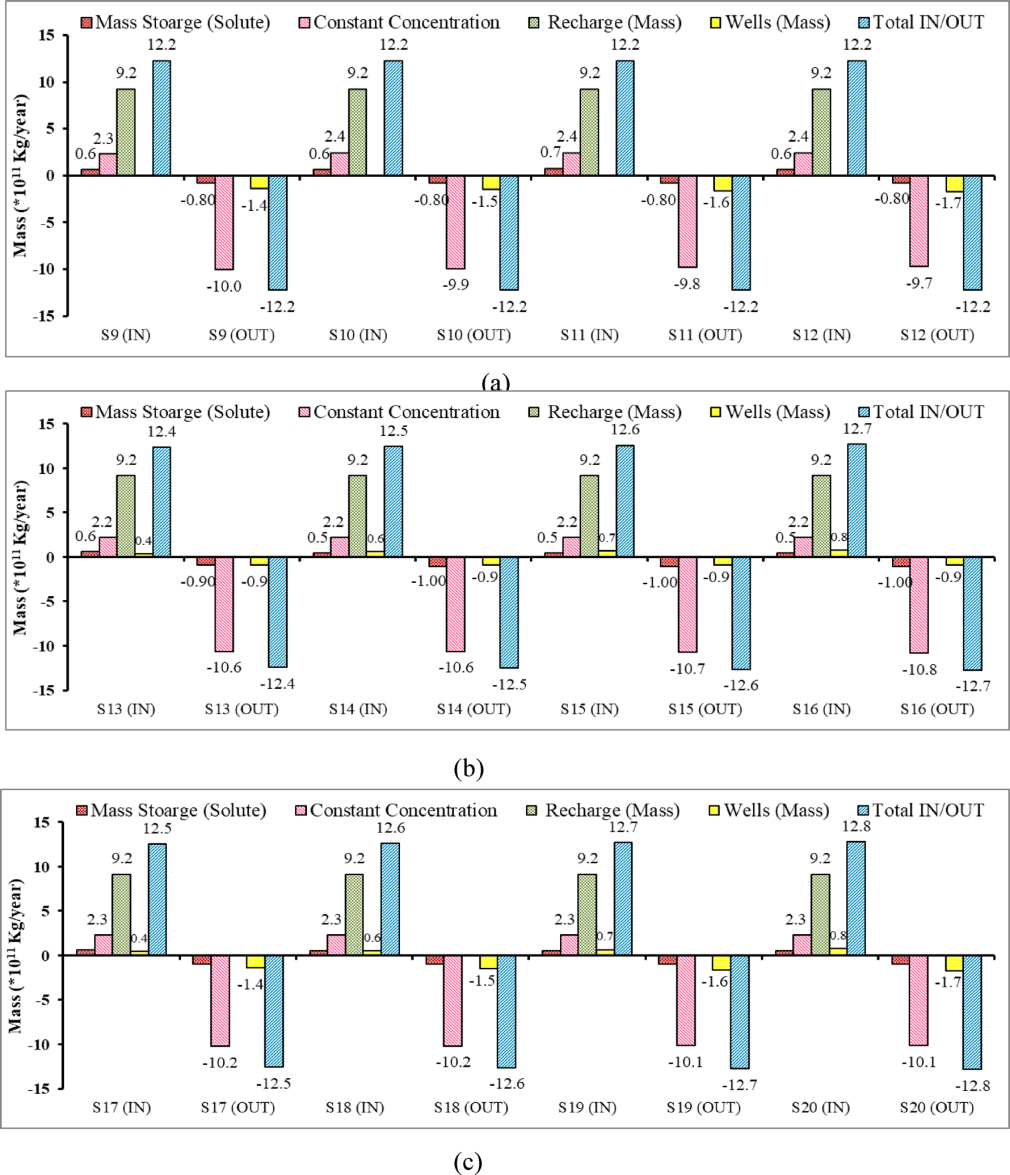
Components of mass balance of the Moghra aquifer for different mitigation of SWI scenarios: (**a**) S9, S10, S11, S12, (**b**) S13, S14, S15, S16, (c) S17, S18, S19, S20.

Figure 24 indicates the solute mass balance of the Moghra aquifer across twelve different mitigation of SWI scenarios (S9 to S12), with each scenario having inflow (IN) and outflow (OUT) components. It can be concluded that the recharge is the major contributor of the mass entering to the aquifer system, followed by mass through boundary condition, and a minor value is observed for solute mass entering the system as a minor contributor. The results of Fig. 24a, b, and c indicates that the mass solute entering the aquifer declined on average to 0.6, 0.5, and 0.4 × 10^11^ kg/year, respectively, due to controlling the SWI through abstraction of saline water (S9 to S12), Recharge (S13 to S16), and a combination of both (S17 to S20) compared with range from 2 to 7.7 × 10^11^ kg/year (S1 to S8) for agricultural management scenarios without conducting any SWI mitigation measures. In S9 to S12, the solute mass declined due to the abstraction of saline water; on the other hand, the freshwater volume is expected to increase. In the S13 to S16 recharge scenarios the mass through the wells reflects the mass of freshwater through this mitigation scenarios. The results confirmed that the combined scenarios S17 to S20 are the best mitigation scenarios to control SWI and achieve sustainable agricultural management in the Moghra region.

### Drawdown in selected groundwater wells

**Fig. 25:**
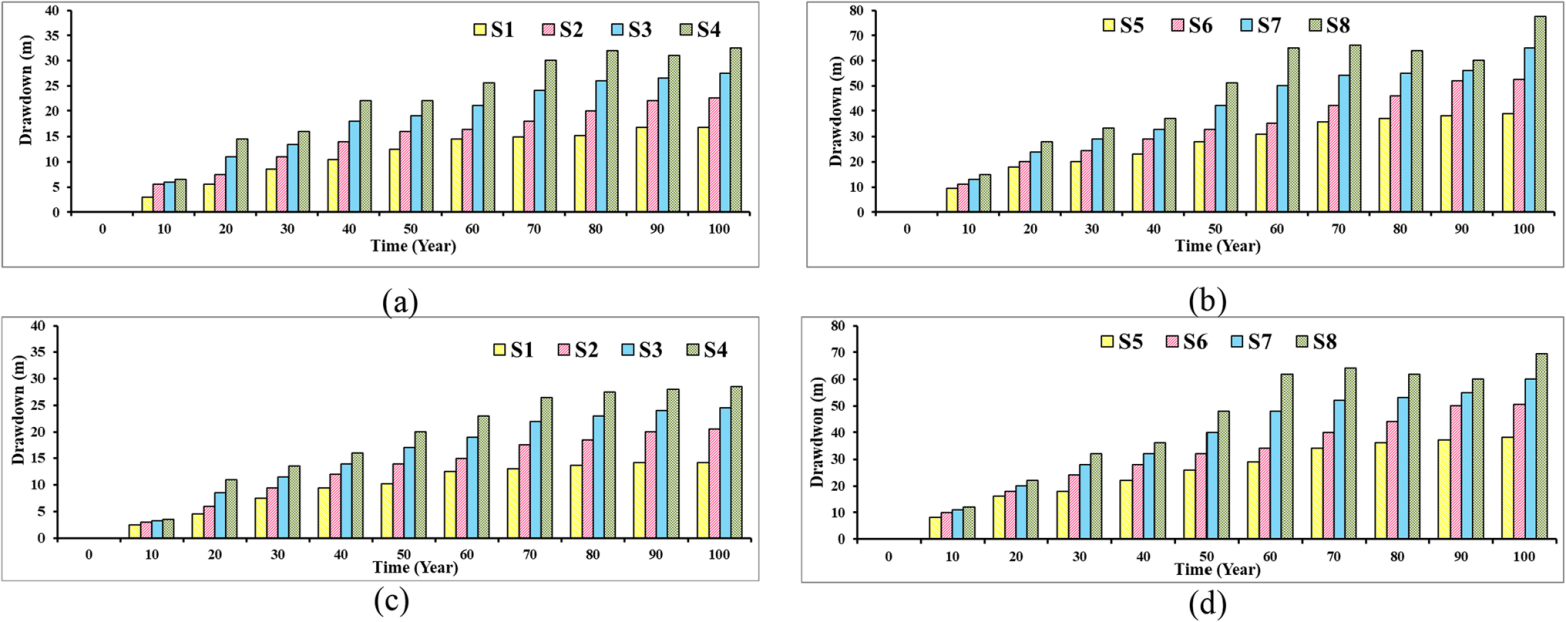
Groundwater level decline in the following wells: (**a**) well (23) with S1, S2, S3, and S4, (**b**) well (23) with S5, S6, S7, and S8, (**c**) well (42) with S1, S2, S3, and S4, (**d**) well (42) with S5, S6, S7, and S8.

Figure 25 illustrates the groundwater level decline in the following wells 23 and 42, near the agricultural development area under different abstraction scenarios from S1 to S8. The results confirmed that the Moghra aquifer is suffering from heavy groundwater abstraction to support the agricultural development plan. This has likely disturbed the natural balance of the dynamics of groundwater flow, and the groundwater level declined over the proposed 100 years plan for different abstraction scenarios. For wells 23 and 42, close to the northern part of the development region, the groundwater level declined gradually for 100 year of development, and the drawdown increases upon increasing the abstraction rate to 32.5 m and 28.5m, respectively for S4. Increasing the pumping rate to 1000, 1250, 1500, 1750 m^3^/day/well for 1,000 wells led to a water level decline over the time in scenarios S5, S6, S7, and S8, respectively. Scenario 8 shows the worst-case scenario, with the sharpest drop in groundwater level, when the drawdown reached 77.5m, and 69.5m, respectively, thus representing poor groundwater management. The decline in groundwater levels in the two selected wells explains how the area is exposed to over-abstraction and confirms the extent of this impact on the intrusion of saline water into the Moghra aquifer and the deterioration of groundwater quality in the study area.

### Salinity variation in selected groundwater wells

**Fig. 26:**
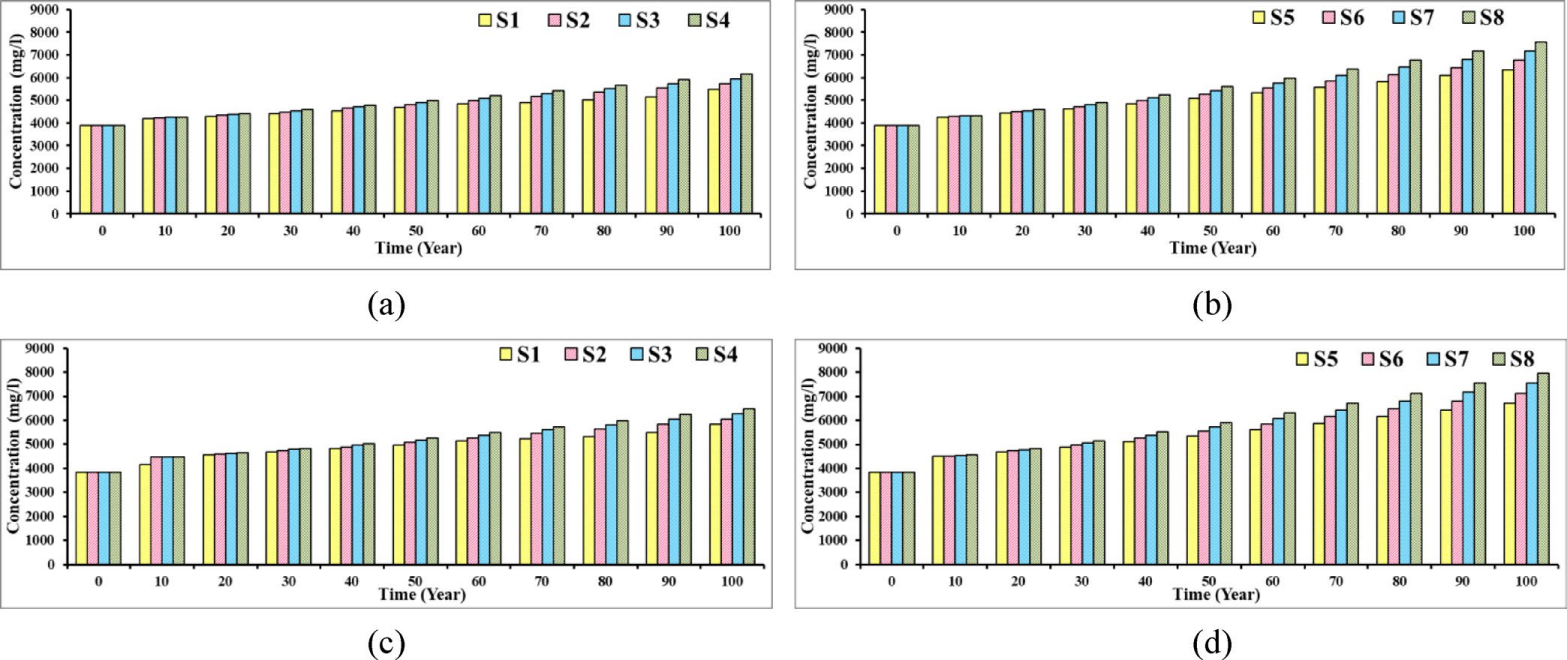
Concentration in two selected wells for different agricultural management scenarios: (**a**), and (**b**) for well 23, (**c**), and (**d**) for well 42.

Figure 26 illustrates the salinity concentration in two selected wells close to the development area in Moghra region for different abstraction scenarios. The outcomes of the figure confirmed that the Moghra region is experiencing seawater intrusion as a result of heavy groundwater pumping and groundwater level decline. This has likely disturbed the natural balance between the fresh groundwater and seawater in the Moghra aquifer, resulting in a gradual increase in the salinity concentration over the 100 years of proposed development plan. For abstraction scenario S4, with total number of wells at 445 and 1750 m^3^/day/well for abstraction rate, for observation well No. 23, close to the development area, the salinity concentration increased from 3875 to 6141 mg/L, and the TDS concentration rose from 3828 to 6481 mg/L for observation well No. 42. Increasing the total number of wells in the study area to reach 1,000 wells resulted in higher rate of TDS increase; S8 in particular considered the worst-case scenario. In addition, for scenario S8, for observation well No. 23, the TDS doubled from 3875 to 7558 mg/L, and the salinity concentration increased dramatically from 3828 to 7953 mg/L for well No. 42. The salinity variations in the two selected wells confirmed how the proposed heavy pumping contribute to increasing the seawater intrusion process and deterioration of the water quality in the study area.

**Fig. 27:**
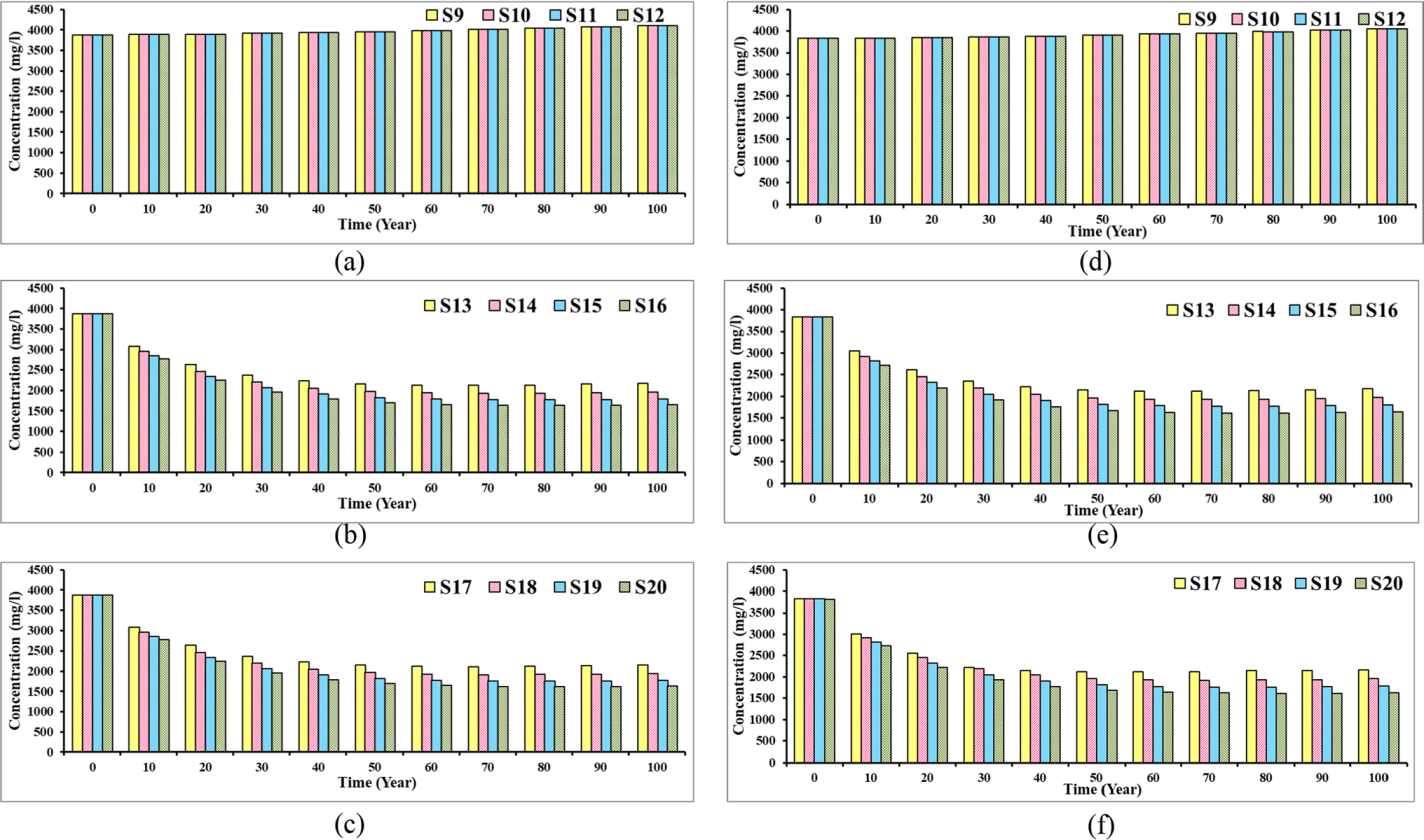
Concentration in two selected wells after conducting different scenarios for the mitigation of SWI: (**a**), (**b**), and (**c**) for well No. 23, (**d**), (**e**), and (**f**) for No. well 42.

Figure 27 presents the salinity concentration in two selected wells, No. 23 and No. 42, close to the development area in the Moghra region. The results from the proposed scenarios could reduce the risk of increased saltwater intrusion and improve the groundwater quality within the study area, thus increasing the likelihood of relying on groundwater resources to support the water requirements for the proposed agricultural reclamation plan. Scenarios S9, S10, S11, S12 depend on the abstraction of saline water near the coast by 100 wells with abstractions equal to 1000, 1250, 1500, 1750 m^3^/day/well. For scenario S12, the TDS concentration in well No. 42 increased slightly from 3875 to 4104 mg/L, and the salinity concentration rose gradually from 3828 mg/L to reach 4050 mg/L. A slight increase in salinity level was observed, and this scenario led to a decline of the groundwater volume of the seawater zone and a slight decrease in the salinity near the coast and had no observable impact in the development area in the Moghra aquifer. Following the results of scenarios S13, S14, S15, and S16 for recharging freshwater through 100 wells by injection rates equal to 1000, 1250, 1500, and 1750 m^3^/day/well, respectively, the salinity levels in the development area declined. In well No. 23, the TDS concentration decreased from 3837 to 1630 mg/L; in addition, the TDS declined from 3827 to 1690 mg/L for well No. 42. The results of these recharge scenarios indicated how recharging through wells is an effective method to increase the freshwater volume and control the salinity level in the study area. Finally, scenarios S17, S18, S19, and S20 present a combination of abstraction of saline water and recharge of freshwater. The results of these scenarios present the best proper method to control the seawater intrusion and improve the water quality level in the development area. The TDS concentration declined gradually over 100 years of the development plan, from 3827 to 1627 mg/L for well No. 23. In addition, the salinity was reduced from 3827 to 1630. This scenario also contributes to a reduction in the salinity level in the development area, as the recharge scenario led to the salinity level in the northern area decreasing, and also contributes to the increasing the freshwater volume and minimizing the seawater zone compared with abstraction only and recharge scenarios only.

### Variation of freshwater volume and saltwater volume in the Moghra aquifer

Table 8 displays the variations of the area and the groundwater volumes for three different zones: the saltwater zone, the mixing zone, and the freshwater zone under various agricultural management and mitigation of seawater intrusion scenarios. The three zones as characterized by various salinity levels in the Moghra aquifer groundwater system are: the saltwater zone contains saline water with 35,000 mg/L concentration, the mixing zone represents the transitional zone where the saltwater and the freshwater mix (1,000 to 35,000 mg/L), and finally the freshwater zone contains the fresh groundwater with salinity below 1,000 mg/L. For agricultural management with the number of wells equal to 445, increasing the abstraction rate in scenarios S1, S2, S3, and S4 led to decline of the groundwater level, and the seawater intruded more into the Moghra aquifer, as a result of which, the seawater volume increased to 101.43, 101.54, 102.06, and 102.38 Km^3^, respectively. In addition, the groundwater volume in the mixing zone increased, though on the other hand, the freshwater volume decreased. From scenarios S5 to S8, the number of wells was increased to 1,000, according to the agricultural plan proposed by the ministry of water resources and irrigation in Egypt. In scenarios S5 to S8, a significant extent in the saltwater and mixing zones is observed, possibly indicating high drawdown with respect to the abstraction strategies in these scenarios. The freshwater volume decreased sharply, particularly in S8 to 108.2 Km^3^ compared with 121.19 km^3^ in the base case scenario. For mitigation of SWI scenarios by abstraction (S9 to S12), a significant shrinkage of the saltwater zone and the mixing zone is observed, possibly indicating the impact of saline water abstraction by this mitigation strategy. The groundwater volume in the saltwater zone declined to 88.8, 87.9, 86.9, and 85.16 km^3^ compared with 101.22 km^3^ in the base case. On the other hand, the freshwater zones extended gradually to reach 94.56 km^3^ in S12. For artificial recharge scenarios as a measure to mitigate SWI, a slight decline in the saltwater zone and mixing zone is observed in the recharge scenarios compared with abstraction scenarios, and the freshwater volume increased gradually in the same scenarios. This indicates the high efficiency of abstraction scenarios to control SWI compared with the recharge scenarios. Finally, in the combined scenarios of abstraction of saline water and recharge of freshwater, the seawater wedge attenuated back more compared with the separate scenarios. From S17 to S20, the saltwater zone and mixing zone decline indicate successful SWI control by these combined scenarios. The groundwater volume in the saltwater zone in scenario S20 declined to reach to 81.9 km^3^ compared with 85.16 km3 and 94.79 km^3^ for S12 (abstraction only) and S16 (recharge only) separately. Decision-makers could utilize the results of these scenarios for managing groundwater resources under various agricultural planning in the Moghra aquifer.

**Table 8:** Areas and volume of saltwater, mixing, and freshwater zones under various agricultural management scenarios and mitigation of SWI scenarios.

Scenario	Saltwater zone		Mixing zone		Freshwater zone	
	Area (km^2^)	GW volume (km^3^)	Area (km^2^)	GW volume (km^3^)	Area (km^2^)	GW volume (km^3^)
Base case	964	101.22	11,049.81	1160.22	1154.19	121.19
*Impact of abstraction on seawater intrusion (no. of wells = 445)*						
S1	966	101.43	11,102.22	1165.73	1099.79	115.48
S2	967	101.54	11,126.47	1168.28	1074.53	112.83
S3	972	102.06	11,149.96	1170.75	1046.04	109.83
S4	975	102.38	11,169.58	1172.81	1023.42	107.49
*Impact of abstraction on seawater intrusion (no. of wells = 1000)*						
S5	970	101.8	11,104.78	1166.00	1093.62	114.83
S6	977	102.2	11,137.61	1169.45	1057.391	111.03
S7	976	102.4	11,141.88	1169.90	1050.596	110.31
S8	981	103.0	11,156.56	1171.44	1030.444	108.20
*Mitigation seawater intrusion scenarios (abstraction)*						
S9	846	88.80	11,513.01	1208.87	809.29	84.98
S10	837	87.90	11,495.52	1207.03	835.34	87.71
S11	828	86.90	11,475.02	1204.88	865.37	90.86
S12	811	85.16	11,456.32	1202.91	900.59	94.56
*Mitigation seawater intrusion scenarios (recharge)*						
S13	937	98.35	11,448.75	1202.12	782.58	82.17
S14	934	98.06	11,445.97	1201.83	788.11	82.75
S15	914	96.01	11,440.64	1201.27	813.00	85.37
S16	903	94.79	11,429.6	1200.11	835.66	87.74
*Mitigation seawater intrusion scenarios (abstraction + recharge)*						
S17	820	86.13	11,579.42	1215.84	768.34	80.68
S18	817	85.75	11,570	1214.85	820.86	86.19
S19	798	83.83	11,560	1213.80	822.32	86.34
S20	780	81.90	11,550	1212.75	825.32	86.66

The limitation of the field data restricts proper calibration and validation of the model for the groundwater level and seawater intrusion. Given the limitations restricting the aquifer model assumptions and the data scarcity, there is not sufficient data for variations of groundwater level and the saltwater intrusion processes over the time and under various pumping rates. In addition, few salinity observations were available in the northern coastal transition zone. The lack of field measurements of groundwater levels and salinity data may have affected the precision of the predictions of groundwater level drawdown and the seawater intrusion wedge under various pumping schemes. To enhance the precision of Moghra aquifer model calibration and validation, future studies should focus on improving the spatial and temporal data resolution for salinity concentration and groundwater level variations, specifically in the area near the coast, where data gaps continue to exist. The limitations of field data may be addressed by a combination of remote sensing approaches with the high-frequency monitoring schemes. Scenario analyses would also be supported by increasing the groundwater model complexity to include the land-use change. Additional practical insights into sustainable groundwater management under the upcoming stressors may also be gained by combination of the hydrological models with the frameworks of socioeconomic decision-making.

## Conclusion

MODFLOW code was used in this research to build a three-dimensional model for the Moghra aquifers in order to simulate the impact of various abstraction schemes on the drawdown of groundwater levels. SEAWAT code was used to simulate the impact of various abstraction conditions on the saltwater invasion and to check the use of different controlling approaches to mitigate saltwater intrusion. Increasing the rate of abstraction significantly increased the drawdown in the development area in the Moghra aquifer. For scenarios S1, S2, S3, S4, the maximum projected drawdown was equal to 24m, 29m, 36m, and 39m over 100 years of agricultural growth, or about 8%, 10%, 12%, and 18% of the saturated Moghra aquifer thickness, respectively. Increasing the number of wells to 1000 to fulfill the water requirement of agricultural development in scenarios S5, S6, and S7, led to an increase in the expected drawdown equal to 54m, 66m, 58m meters after 100 years of agricultural development, or about 18%, 22%, and 28% of the saturated Moghra aquifer thickness, consequently. Scenario S8 presents the optimum scenario, where the greatest anticipated drawdown in the Moghra equals 100m for 100 years of agricultural development. And increasing the abstraction rate more than 1750 m^3^/day/well is expected to increase the drawdown by more than one-third of the saturated depth of the aquifer. Seawater intrusion inland into the Moghra aquifer is significantly impacted by increasing the rate of abstraction. For scenarios S1, S2, S3, and S4, the salinity concentration in the Moghra region ranged from 2400 to 4657 mg/L, 2431 to 4740 mg/L, 2441 to 5005 mg/L, and from 2460 to 5279 mg/L, consequentially. For scenarios S5, S6, S7, and S8, the salinity concentration in the Moghra region ranged from 2456 to 5552 mg/L, 2460 to 5485 mg/L, 2476 to 6143 mg/L, and from 2482 to 6439 mg/L, consequently. The outcomes confirmed that the Moghra region is experiencing seawater intrusion as a result of heavy groundwater pumping and groundwater level decline. This has likely disturbed the natural balance between the fresh groundwater and seawater in the Moghra aquifer and resulted in a gradual increase in the salinity concentration and deterioration of water quality over the 100 years of proposed development plan. The consequential increase in the Moghra aquifer salinity calls into question the suitability of groundwater for the desired agricultural purposes.

For controlling saltwater intrusion in the Moghra aquifer, three different techniques were checked. Through abstraction of saline water scenarios (S9, S10, S11, and S12), the salinity was minimized and ranged between 2431 to 4985 mg/L, 2420 to 4894 mg/L, 2410 to 4694 mg/L, and from 2400 to 4594 mg/L, consequently. In addition, in artificial recharge scenarios (S13, S14, S15, and S16), the salinity declined and ranged between 2430 to 4629 mg/L, 2420 to 4581 mg/L, 2410 to 4358 mg/L, and from 2400 to 4300 mg/L, respectively. The combination of saline water extraction and artificial recharge (S17, S18, S19, and S20) led to attenuating back the seawater towards the sea. Future studies should combine frequent monitoring with remote sensing to improve data resolution, particularly close to the coast, in order to improve the Moghra aquifer model. Better groundwater management can also be supported by integrating hydrological models with socioeconomic decision-making and increasing model complexity to account for changes in land use. The findings of this study can be used by decision-makers to manage groundwater abstraction, saltwater invasion and sustainable groundwater management in arid and semi-arid coastal aquifers.

## Supplementary Information

Below is the link to the electronic supplementary material.

Supplementary Material 1

## Data Availability

Data, models, or codes that support the findings of this study are available from the corresponding author upon reasonable request.
