# Supplementary material for: Management of groundwater abstraction and seawater intrusion in the Moghra aquifer, Egypt
Source: Sci Rep. 2025 Aug 19;15:30385. doi: 10.1038/s41598-025-14432-y (PMC12365243; doi:10.1038/s41598-025-14432-y)
Supplement: Supplementary file 1 — Supplementary Material 1 [file 41598_2025_14432_MOESM1_ESM.docx]

# Appendix File:

# Management of groundwater abstraction and seawater intrusion in the Moghra aquifer, Egypt

| 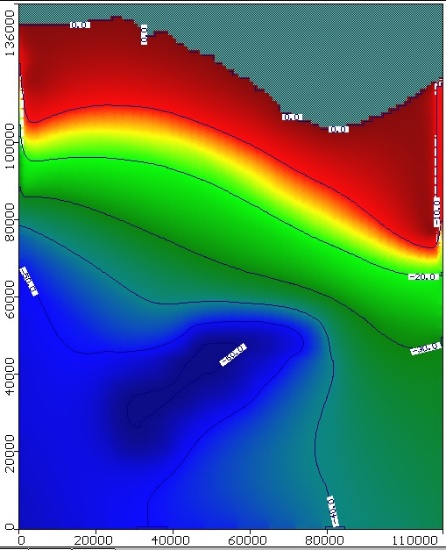  (a) | 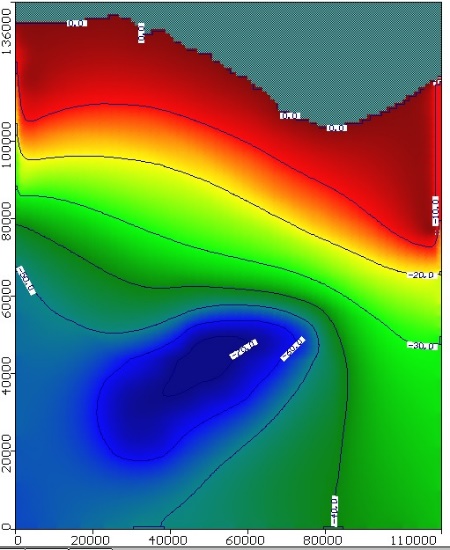  (b) | 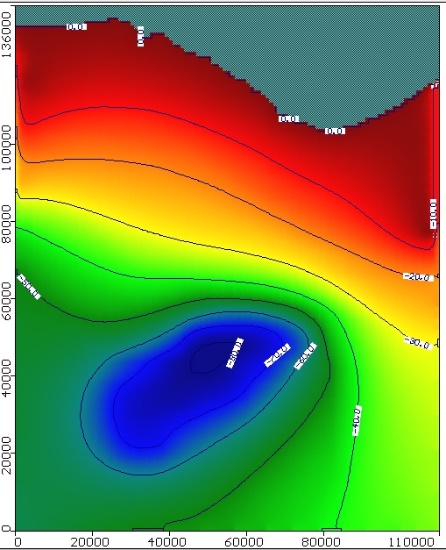  (c) |
| --- | --- | --- |
| 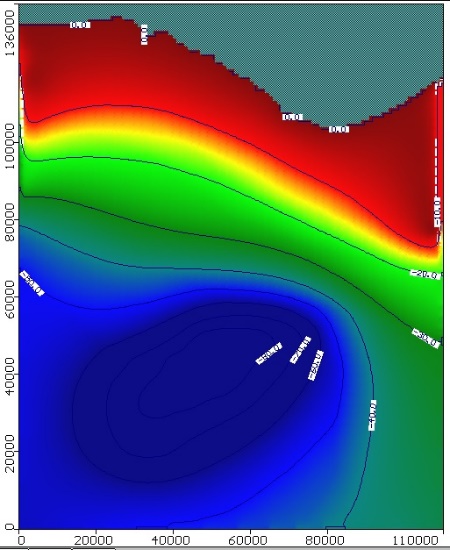  (d) | 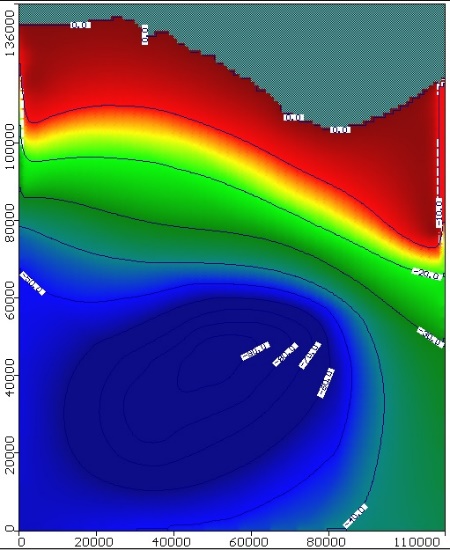  (e) | 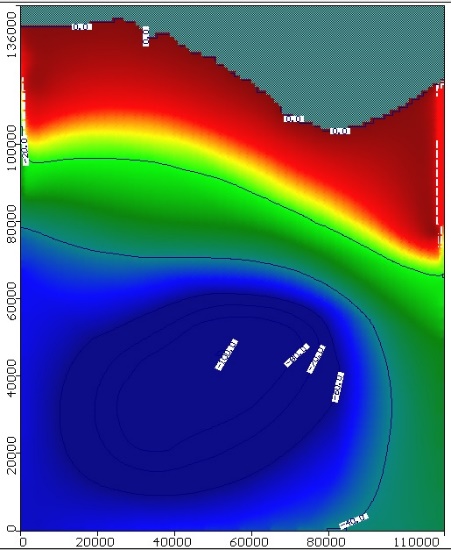  (f) |
| 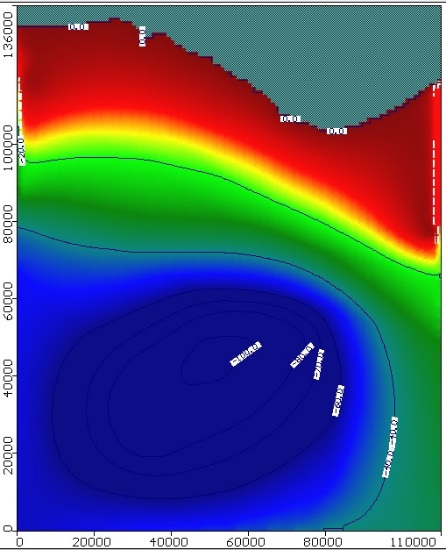  (g) | 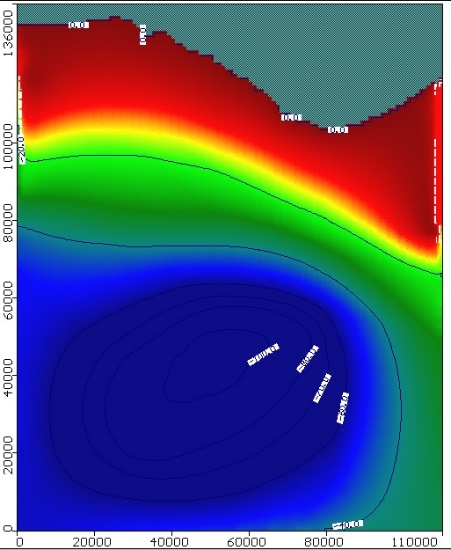  (h) | 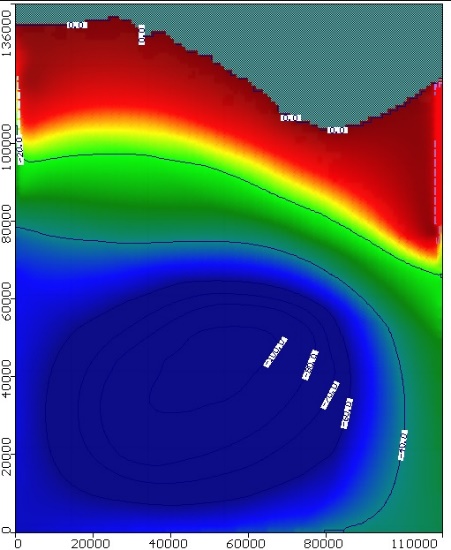  (i) |
| 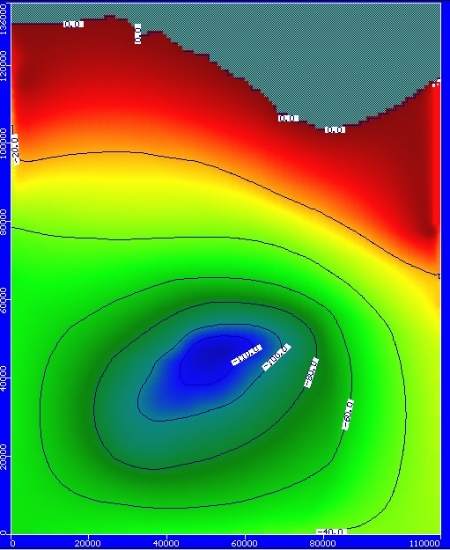  (j) |  |  |

**Figure A.1** Simulation groundwater level in the Moghra aquifer for scenario No.6: 1000 wells with abstraction rate 1500 m^3^/day/well through 100 test period: (a) 10, (b) 20, (c) 30, (d) 40, (e) 50, (f) 60, (g) 70, (h) 80, (i) 90, and (j) 100 years

| 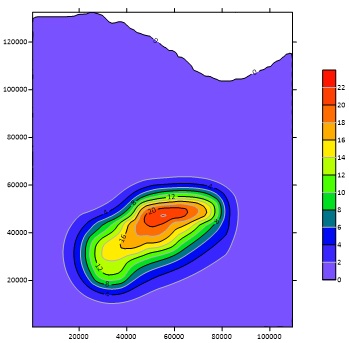  (a) | 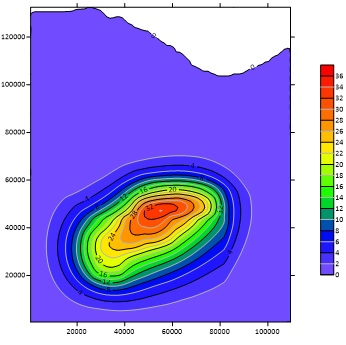  (b) | 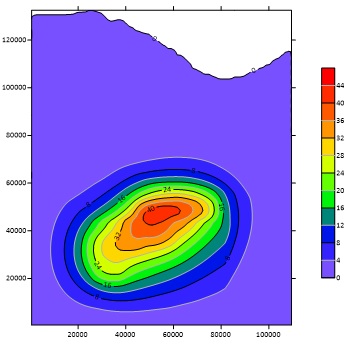  (c) |
| --- | --- | --- |
| 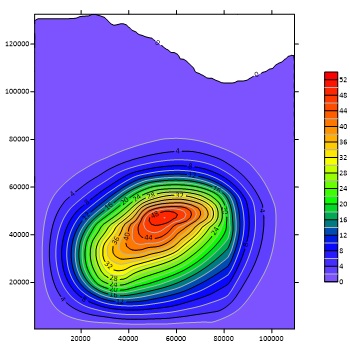  (d) | 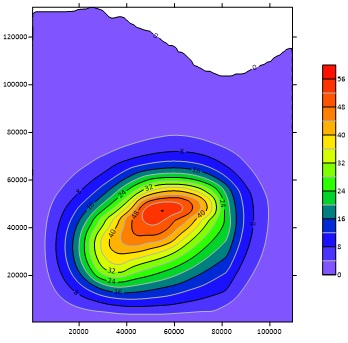  (e) | 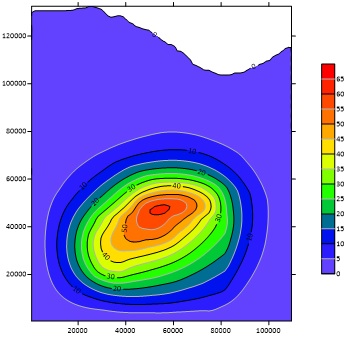  (f) |
| 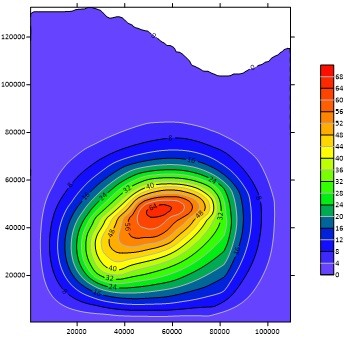  (g) | 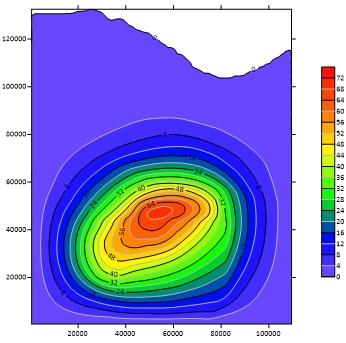  (h) | 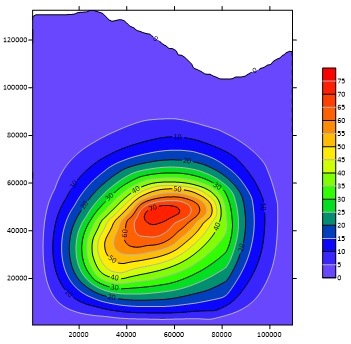  (i) |
| 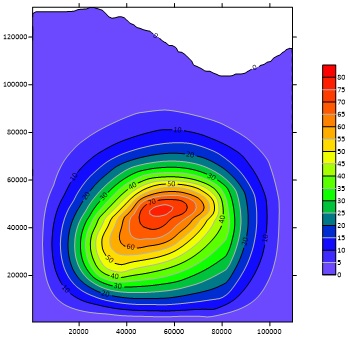  (j) |  |  |

**Figure A.2** Simulation groundwater level in the Moghra aquifer for scenario No.6: 1000 wells with abstraction rate 1500 m^3^/day/well through 100 test period: (a) 10, (b) 20, (c) 30, (d) 40, (e) 50, (f) 60, (g) 70, (h) 80, (i) 90, and (j) 100 years

**Seawater intrusion in the Moghra aquifer:**

**Cross section No.1:**

| 1. 1000 year | 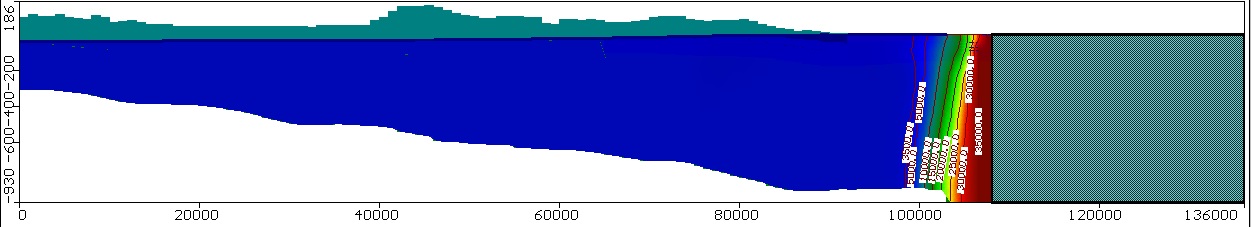 |
| --- | --- |
| 1. 2000 year | 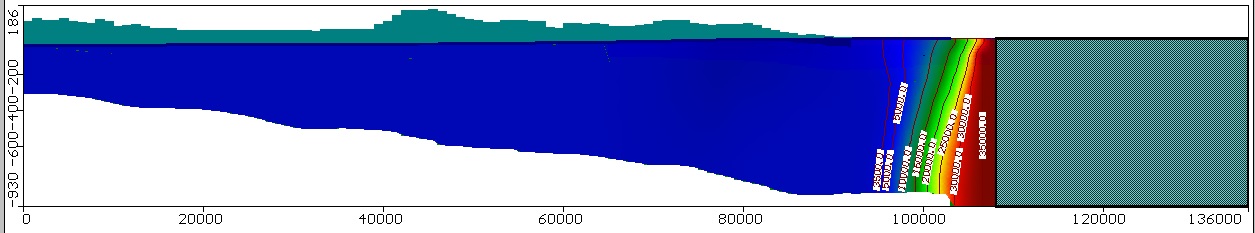 |
| 1. 3000 years | 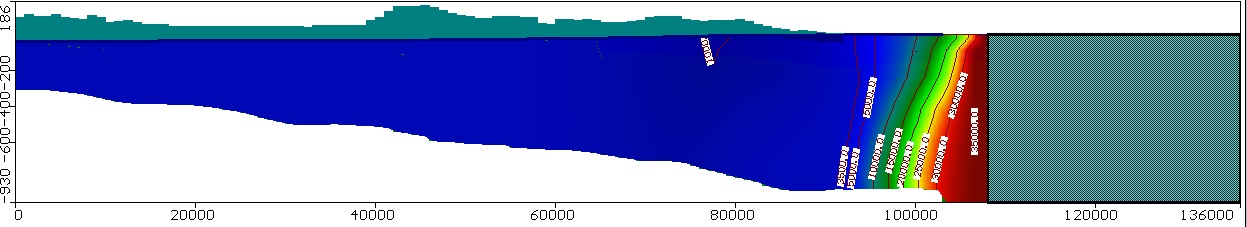 |
| 1. 4000 years | 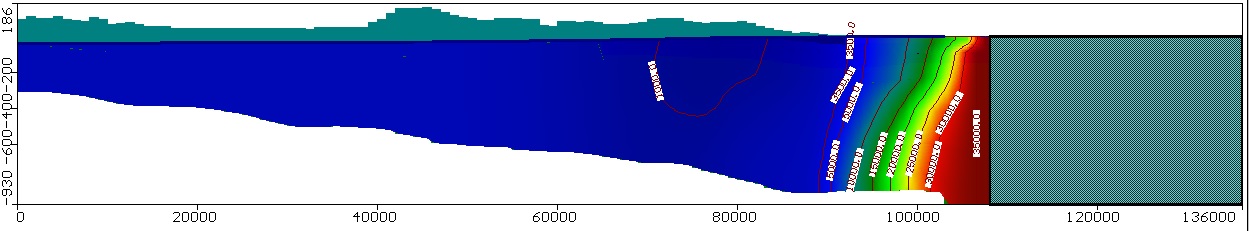 |
| 1. 5000 years | 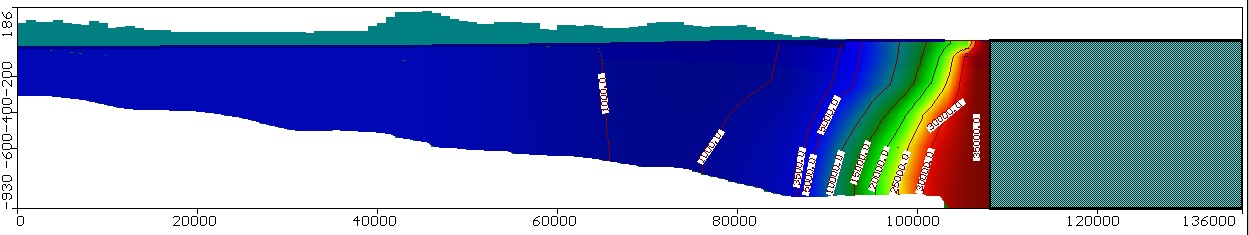 |
| 1. 6000 years | 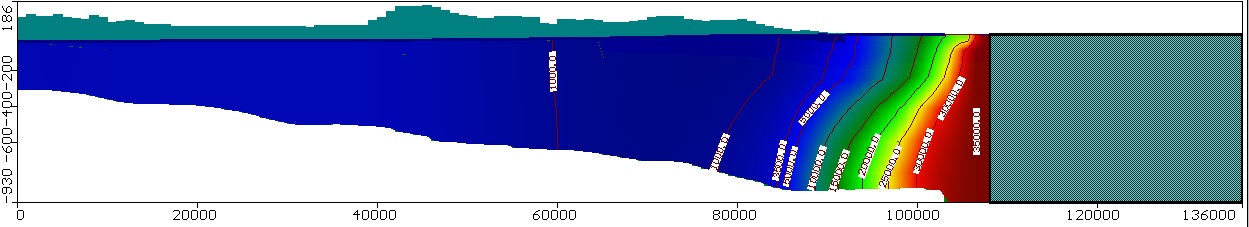 |
| 1. 7000 years | 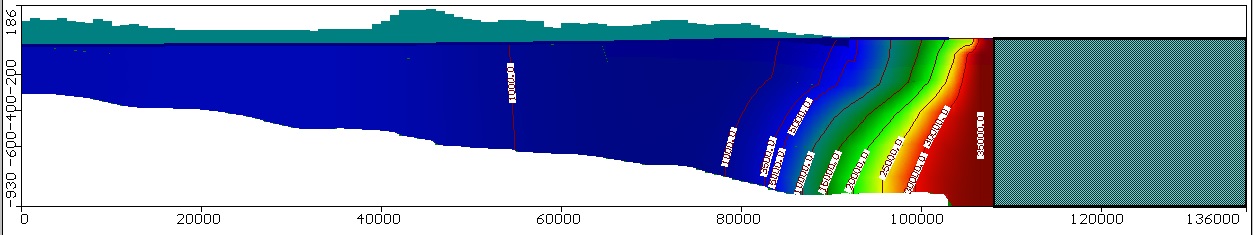 |
| 1. 8000 years | 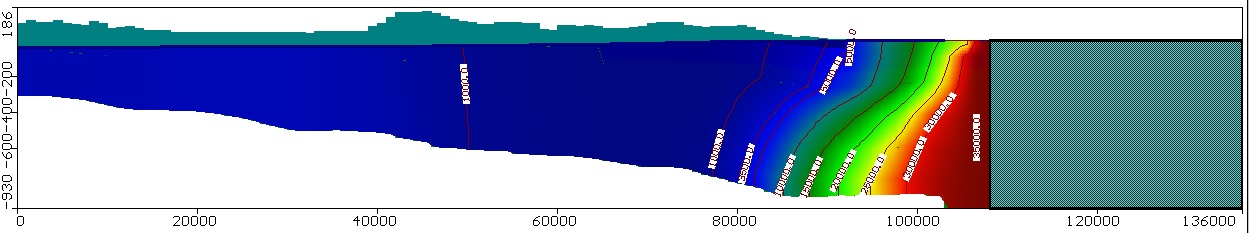 |

**Figure A.3** Advancement of seawater distribution in cross section No.1 in the Moghra aquifer for years (a) 1000, (b) 2000, (c) 3000, (d) 4000, (e) 5000, (f) 6000, (g) 7000, and (h) 8000 years

**Cross section No.2:**

| 1. 1000 year | 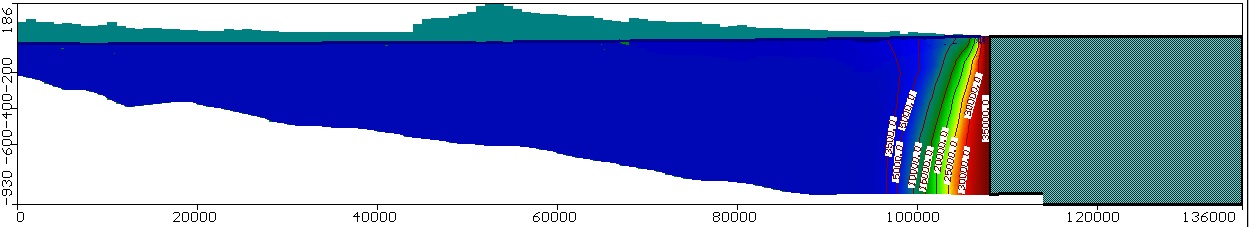 |
| --- | --- |
| 1. 2000 year | 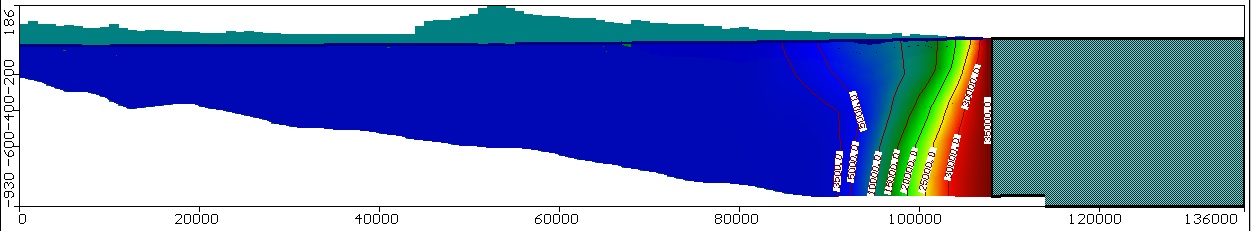 |
| 1. 3000 years | 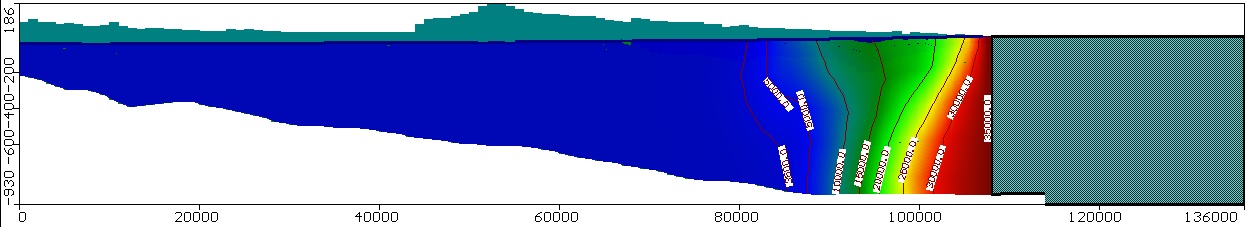 |
| 1. 4000 years | 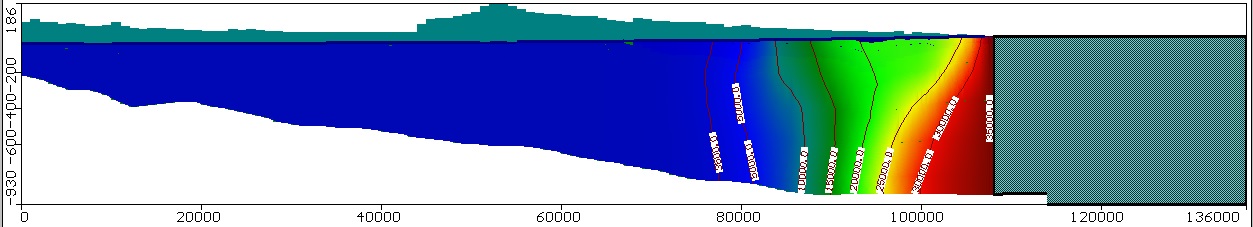 |
| (e) 5000 years | 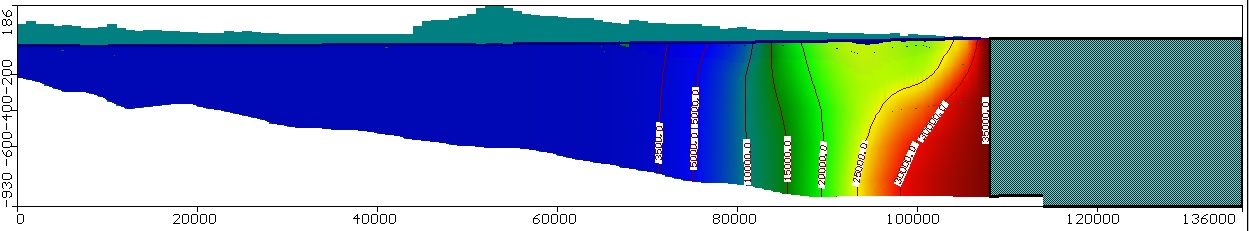 |
| 1. 6000 years | 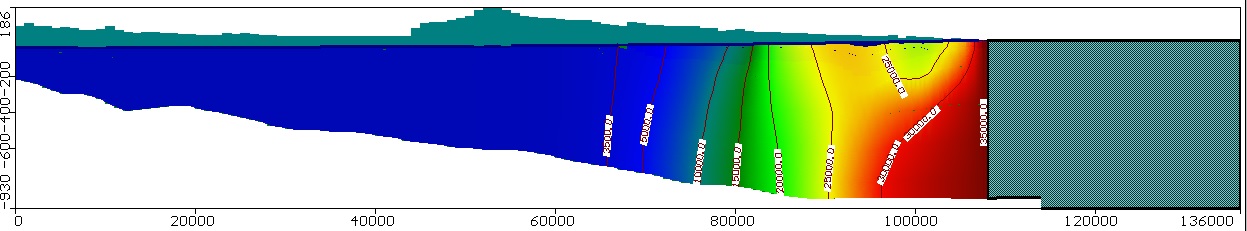 |
| 1. 7000 years | 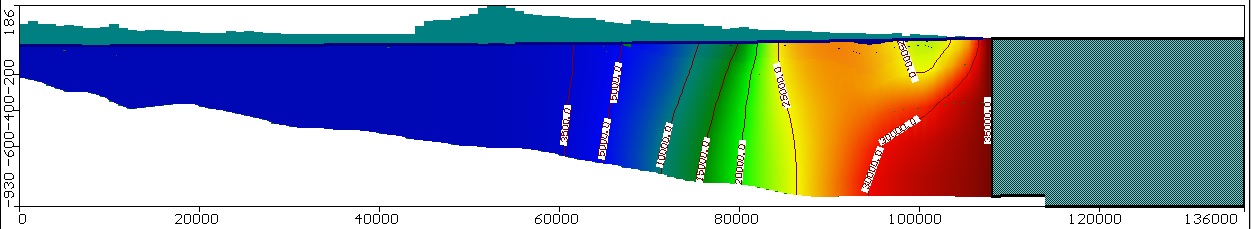 |
| 1. 8000 years | 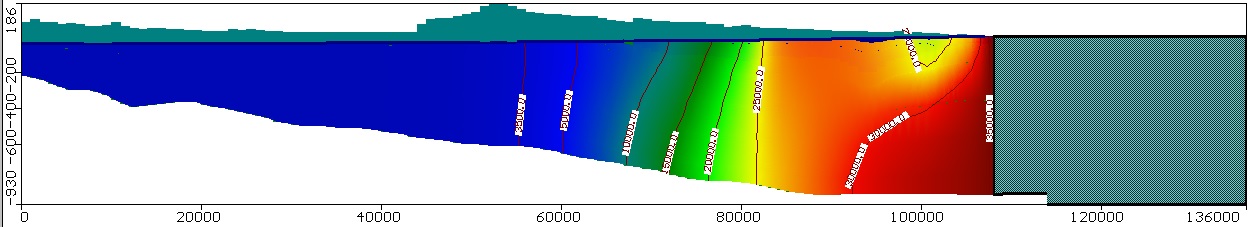 |

**Figure A.4** Advancement of seawater distribution in cross section No.2 in the Moghra aquifer for years: (a) 1000, (b) 2000, (c) 3000, (d) 4000, (e) 5000, (f) 6000, (g) 7000, and (h) 8000 years

**Cross section No.3:**

| 1. 1000 year | 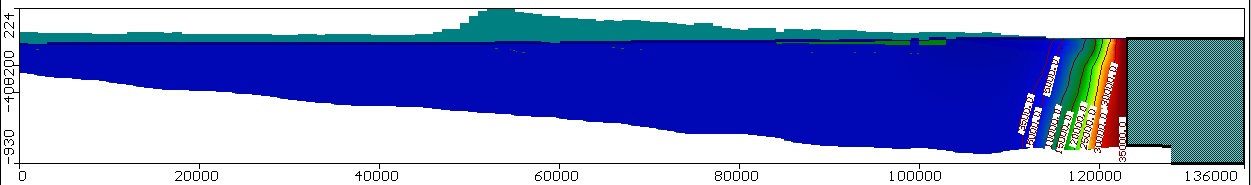 |
| --- | --- |
| 1. 2000 year | 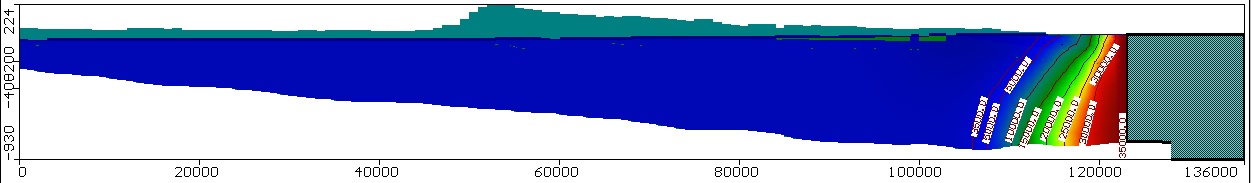 |
| 1. 3000 years | 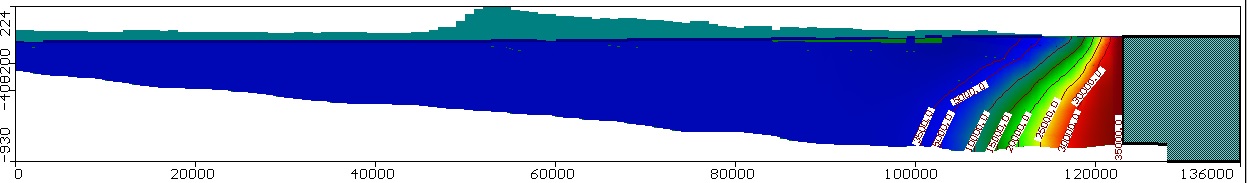 |
| 1. 4000 years | 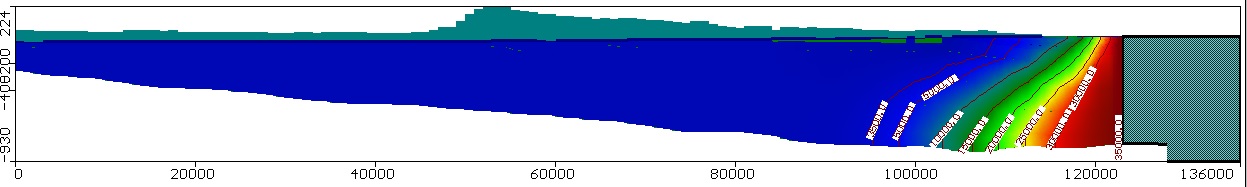 |
| 1. 5000 years | 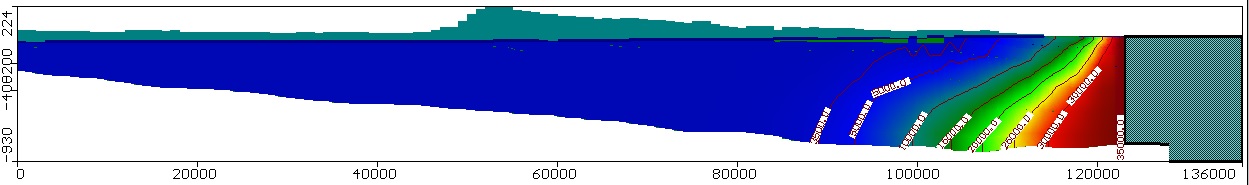 |
| 1. 6000 years | 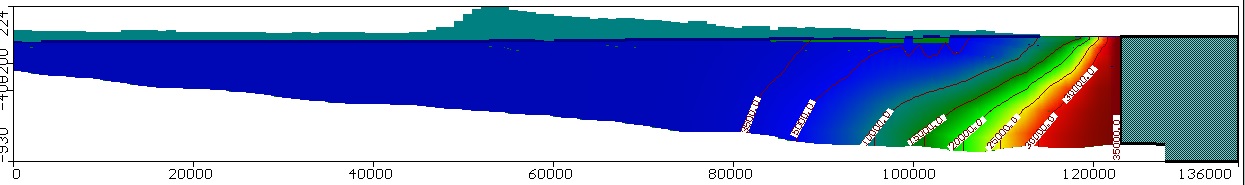 |
| 1. 7000 years | 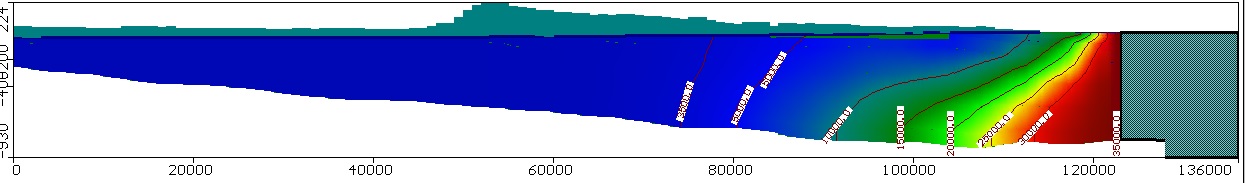 |
| 1. 8000 years | 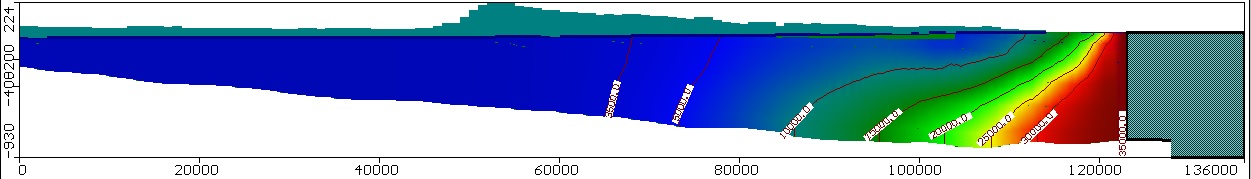 |

**Figure A.5** Advancement of seawater distribution in cross section No.3 in the Moghra aquifer for years: (a) 1000, (b) 2000, (c) 3000, (d) 4000, (e) 5000, (f) 6000, (g) 7000, and (h) 8000 years

**Impact of groundwater abstraction in seawater intrusion in Moghra aquifer**

**(Scenarios No.5, 6, 7 and 8):**

**Cross section No.1:**

| 1. **Steady state** | 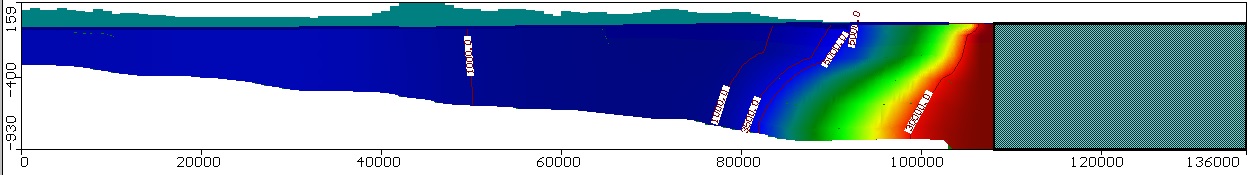 |
| --- | --- |
| 1. **1000**   **m^3^/day/well** | 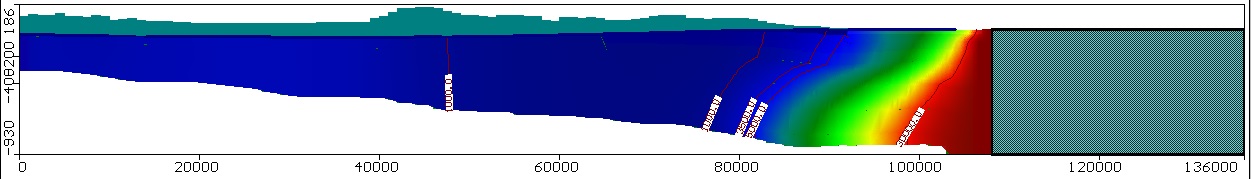 |
| 1. **1250**   **m^3^/day/well** | 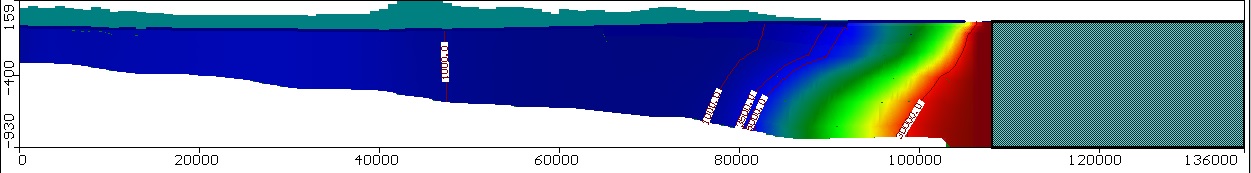 |
| 1. **1500**   **m^3^/day/well** | 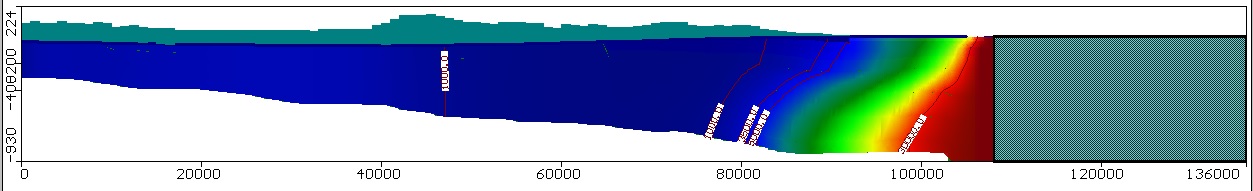 |
| 1. **1750**   **m^3^/day/well** | 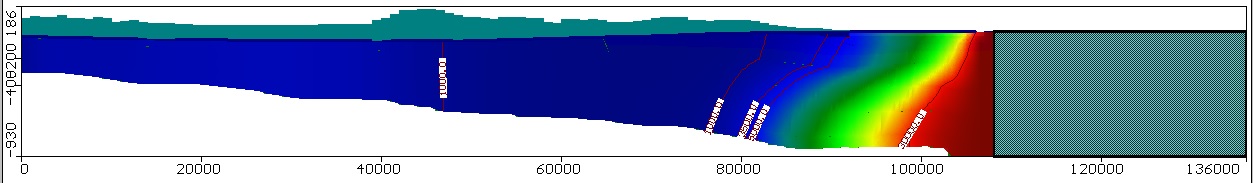 |
| 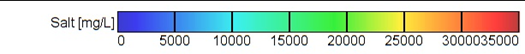 | |

Figure A.6 Seawater distribution in vertical cross-section No.1 in the Moghra aquifer for Scenarios 5 to 8 (1000 wells): (a) 1000 m^3^/day/well, (b) 1250 m^3^/day/well, (c) 1500 m^3^/day/well, and (d) 1750 m^3^/day/well

| 1. **Steady state** | 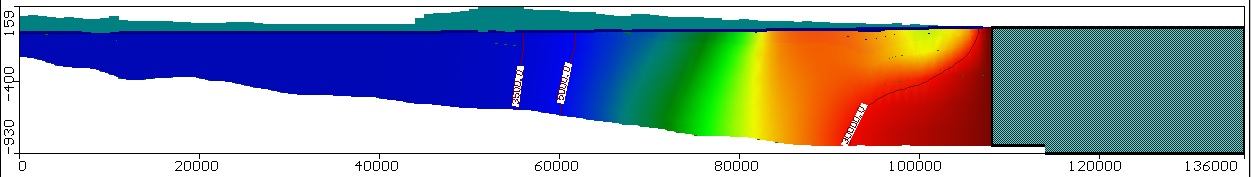 |
| --- | --- |
| 1. **1000**   **m^3^/day/well** | 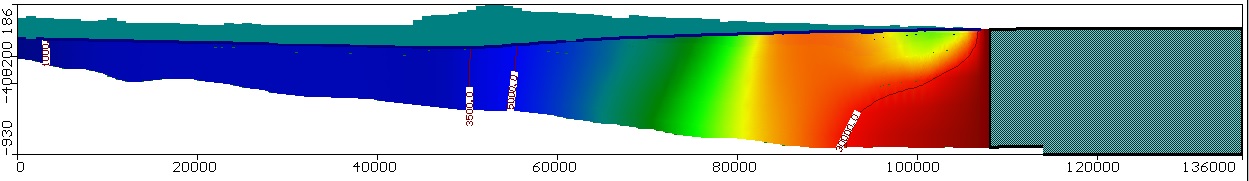 |
| 1. **1250**   **m^3^/day/well** | 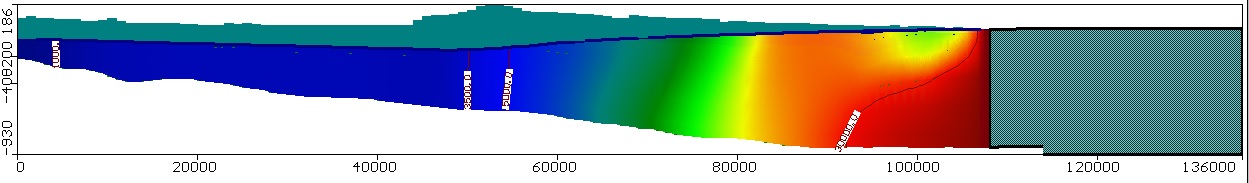 |
| 1. **1500**   **m^3^/day/well** | 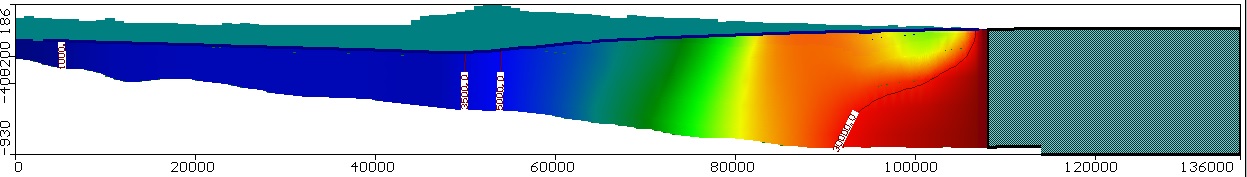 |
| 1. **1750**   **m^3^/day/well** | 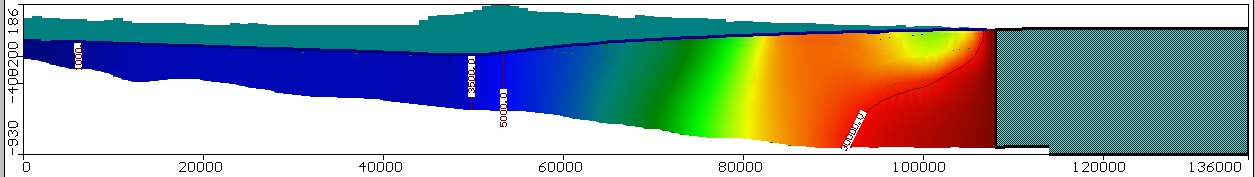 |
| 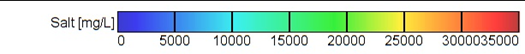 | |

Figure A.7 Seawater distribution in vertical cross-section No.2 in the Moghra aquifer for Scenarios 5 to 8 (1000 wells): (a) 1000 m^3^/day/well, (b) 1250 m^3^/day/well, (c) 1500 m^3^/day/well, and (d) 1750 m^3^/day/well

| 1. **Steady state** | 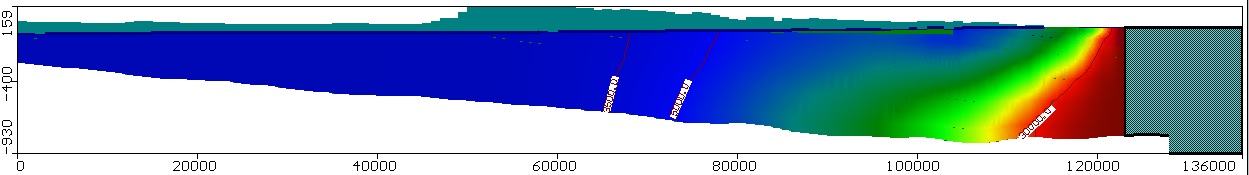 |
| --- | --- |
| 1. **1000**   **m^3^/day/well** | 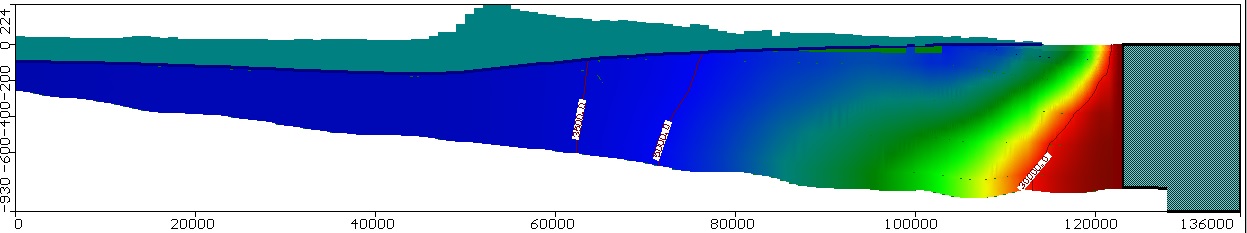 |
| 1. **1250**   **m^3^/day/well** | 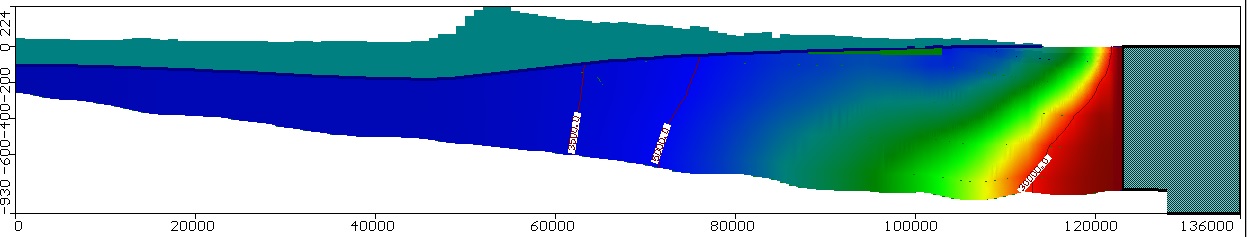 |
| 1. **1500**   **m^3^/day/well** | 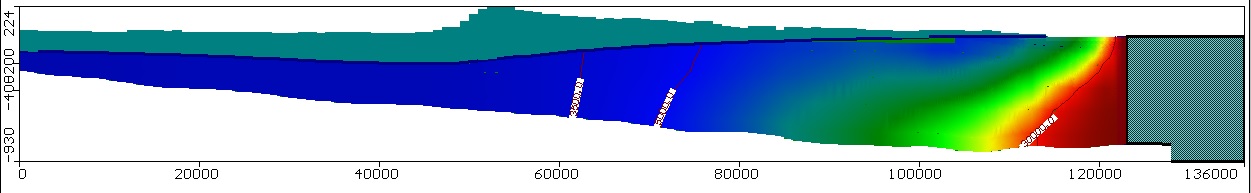 |
| **(e) 1750**  **m^3^/day/well** | 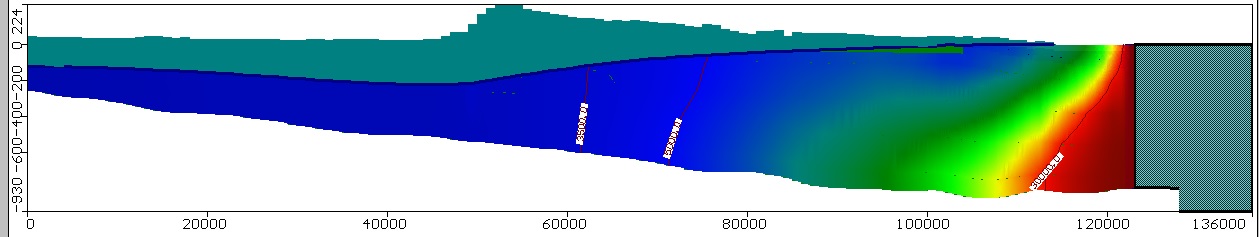 |
| 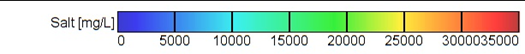 | |

Figure A.8 Seawater distribution in vertical cross-section No.3 in the Moghra aquifer for Scenarios 5 to 8 (1000 wells): (a) 1000 m^3^/day/well, (b) 1250 m^3^/day/well, (c) 1500 m^3^/day/well, and (d) 1750 m^3^/day/well

**Mitigation of seawater intrusion in Moghra aquifer:**

**Impact of saline water abstraction to control seawater intrusion in the Moghra aquifer**

**Scenarios 9, 10, 11, and 12**

**Cross section No.1:**

| 1. **Base-case** | 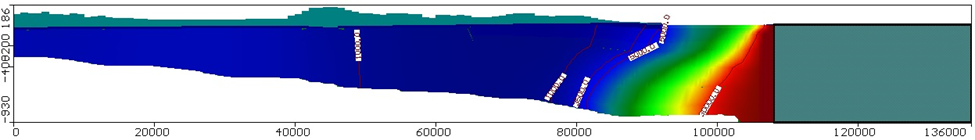 |
| --- | --- |
| 1. **1000**   **m^3^/day/well** | 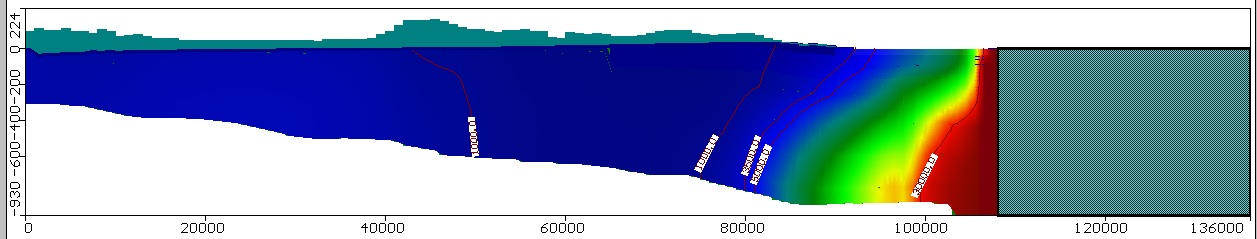 |
| 1. **1250**   **m^3^/day/well** | 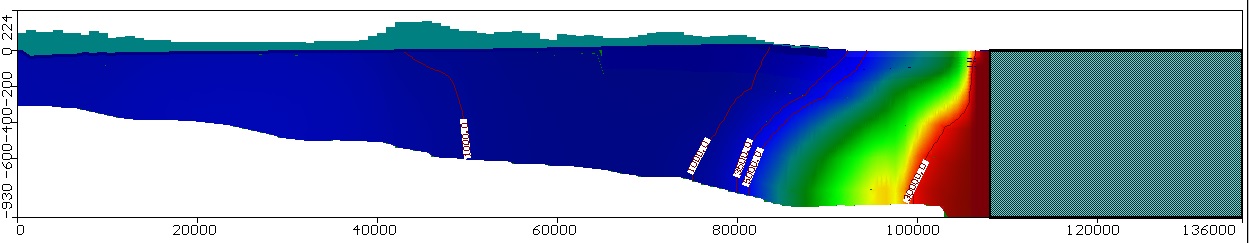 |
| 1. **1500**   **m^3^/day/well** | 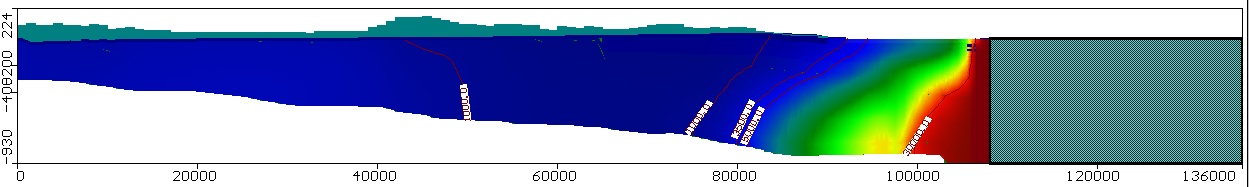 |
| 1. **1750**   **m^3^/day/well** | 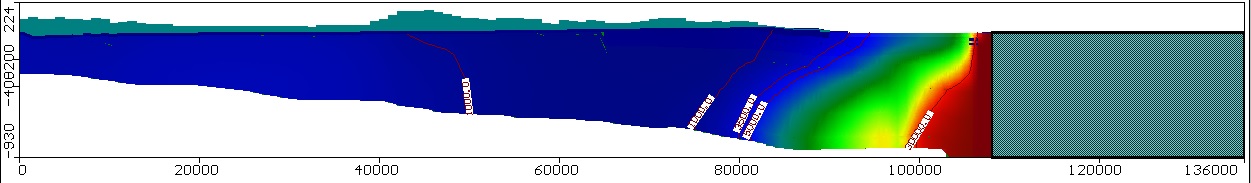 |
| 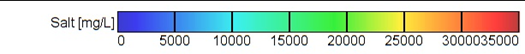 | |

**Figure A.9** Seawater distribution in vertical cross-section No.1 in the Moghra aquifer for the abstraction of saline water controlling scenarios 9 to 12 (1000 wells): (a) 1000 m^3^/day/well, (b) 1250 m^3^/day/well, (c) 1500 m^3^/day/well, and (d) 1750 m^3^/day/well

**Cross section No.2:**

| 1. **Base case** | 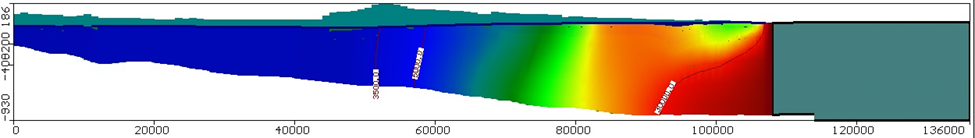 |
| --- | --- |
| 1. **1000**   **m^3^/day/well** | 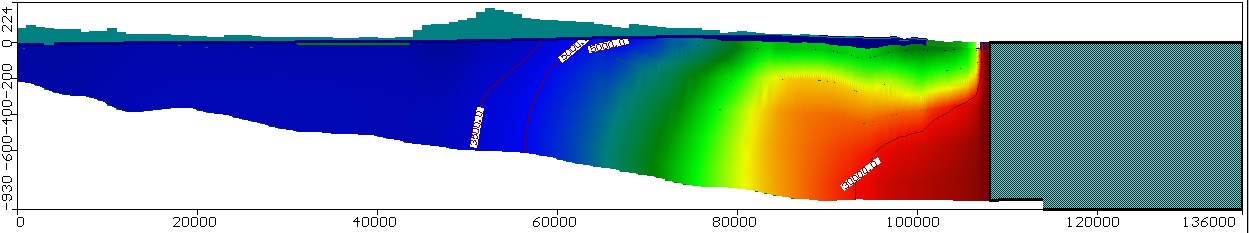 |
| 1. **1250**   **m^3^/day/well** | 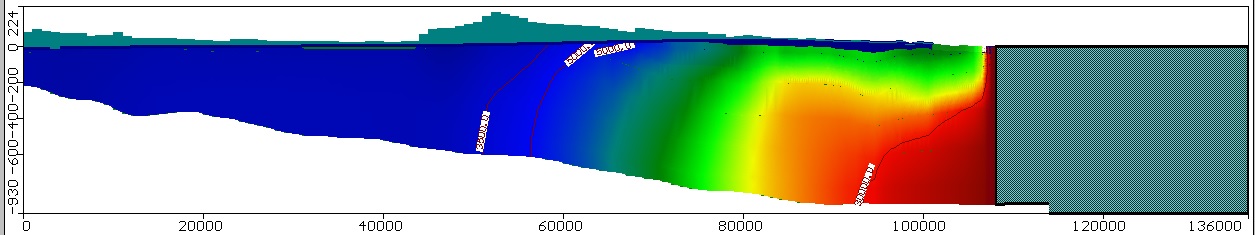 |
| 1. **1500**   **m^3^/day/well** | 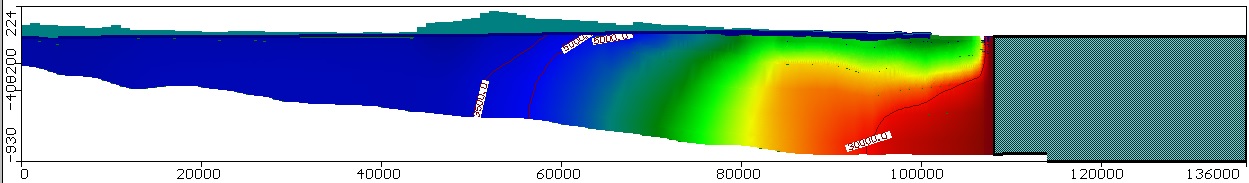 |
| 1. **1750**   **m^3^/day/well** | 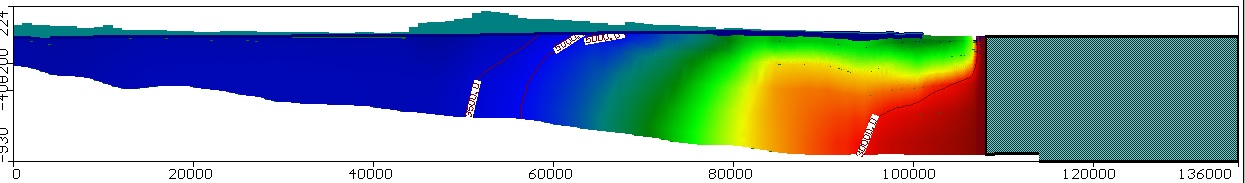 |
| 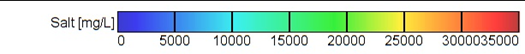 | |

**Figure A.10** Seawater distribution in vertical cross-section No.2 in the Moghra aquifer for the abstraction of saline water controlling scenarios 9 to 12 (1000 wells): (a) 1000 m^3^/day/well, (b) 1250 m^3^/day/well, (c) 1500 m^3^/day/well, and (d) 1750 m^3^/day/well

**Cross section No.3:**

| 1. **Base case** | 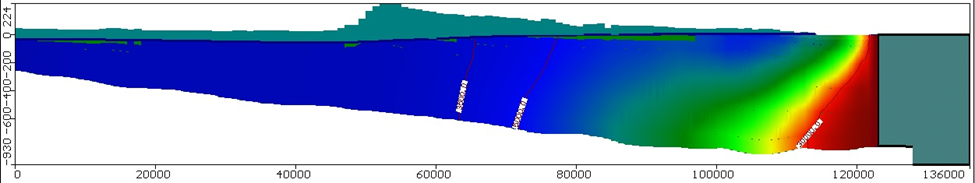 |
| --- | --- |
| 1. **1000**   **m^3^/day/well** | 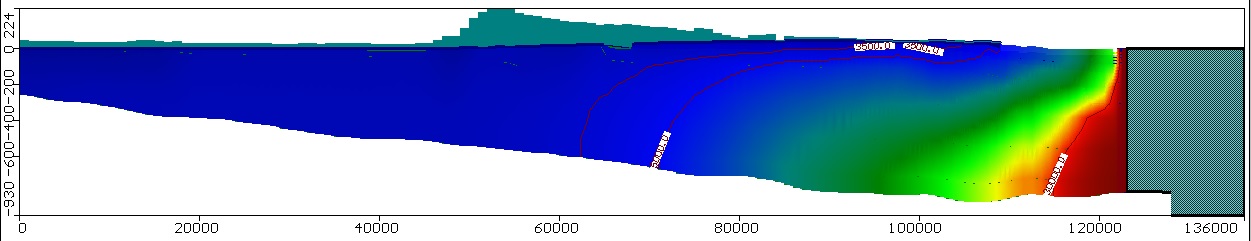 |
| 1. **1250**   **m^3^/day/well** | 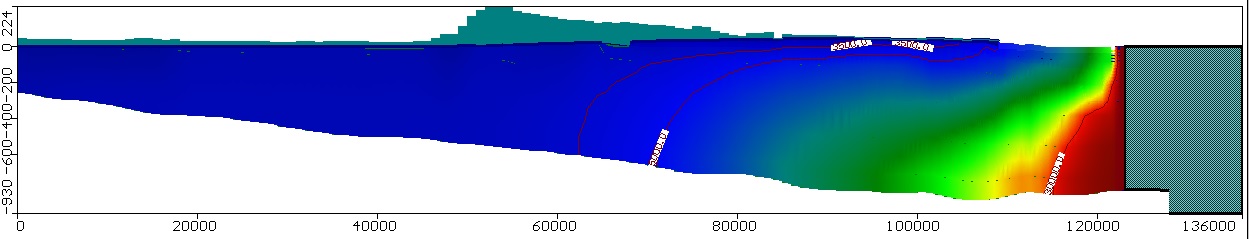 |
| 1. **1500**   **m^3^/day/well** | 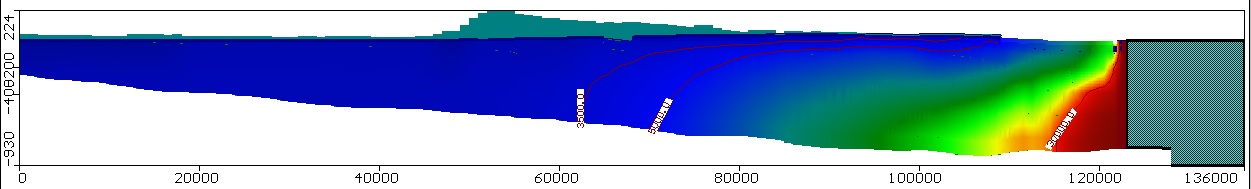 |
| 1. **1750**   **m^3^/day/well** | 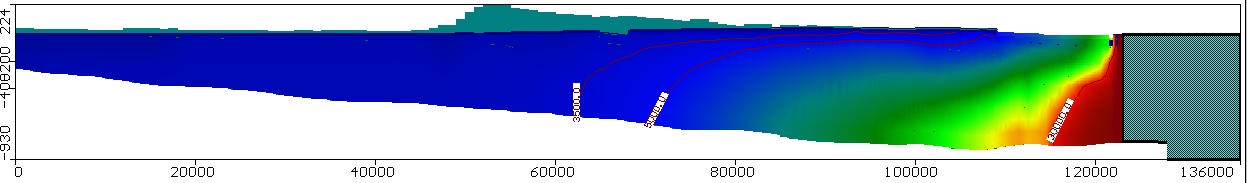 |
| 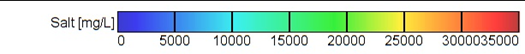 | |

**Figure A.11** Seawater distribution in vertical cross-section No.3 in the Moghra aquifer for the abstraction of saline water controlling scenarios 9 to 12 (1000 wells): (a) 1000 m^3^/day/well, (b) 1250 m^3^/day/well, (c) 1500 m^3^/day/well, and (d) 1750 m^3^/day/well

**Impact of Artificial recharge to control seawater intrusion in the Moghra aquifer**

**Scenarios 13, 14, 15, and 16**

**Cross section No.1:**

| 1. Base case | 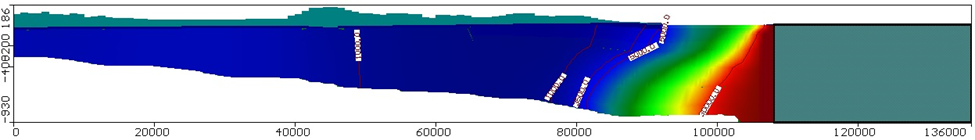 |
| --- | --- |
| 1. 1000   m^3^/day/well | 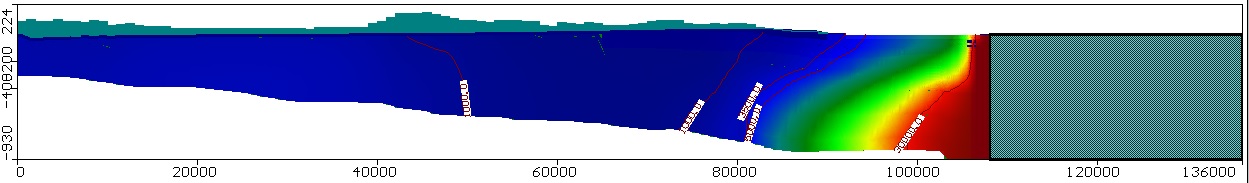 |
| 1. 1250   m^3^/day/well | 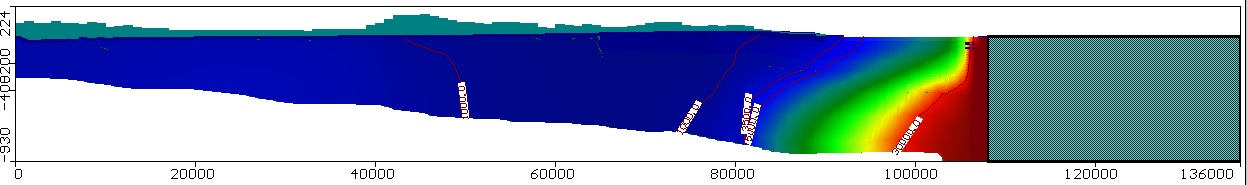 |
| 1. 1500   m^3^/day/well | 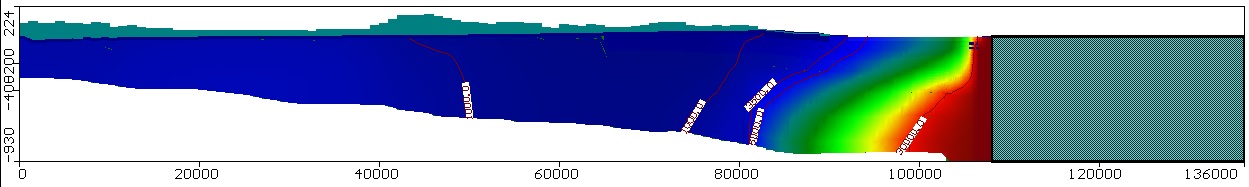 |
| 1. 1750   m^3^/day/well | 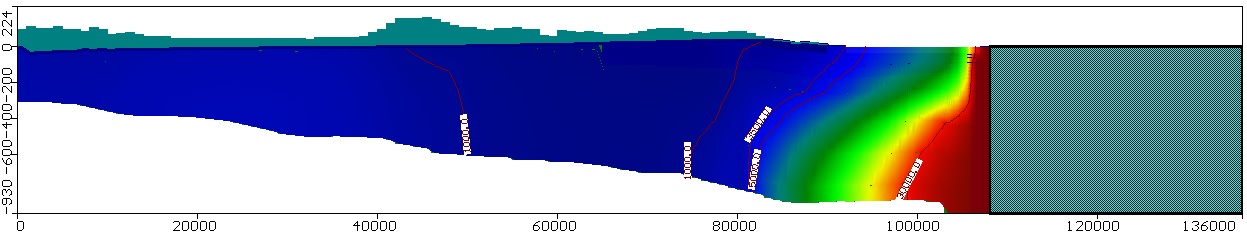 |
| 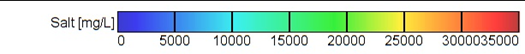 | |

**Figure A.12** Seawater distributions in vertical cross-section No.1 in the Moghra aquifer for artificial recharge controlling scenarios 13 to 16: (a) 1000 m^3^/day/well, (b) 1250 m^3^/day/well, (c) 1500 m^3^/day/well, and (d) 1750 m^3^/day/well

**Cross section No.2:**

| 1. **Base case** | 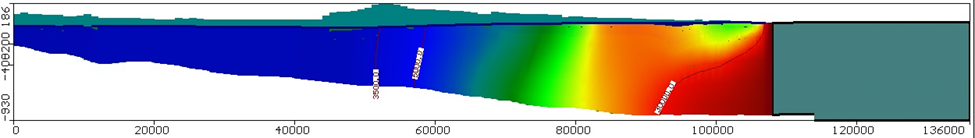 |
| --- | --- |
| 1. **1000**   **m^3^/day/well** | 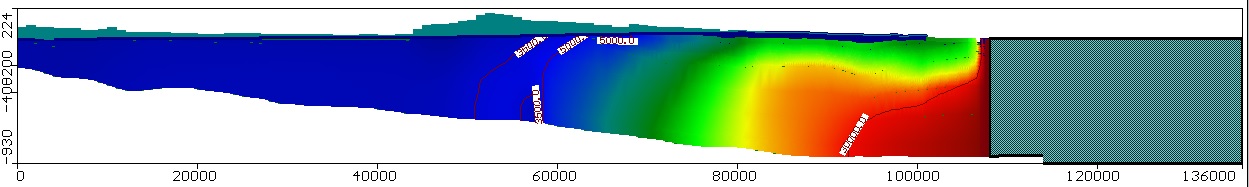 |
| 1. **1250**   **m^3^/day/well** | 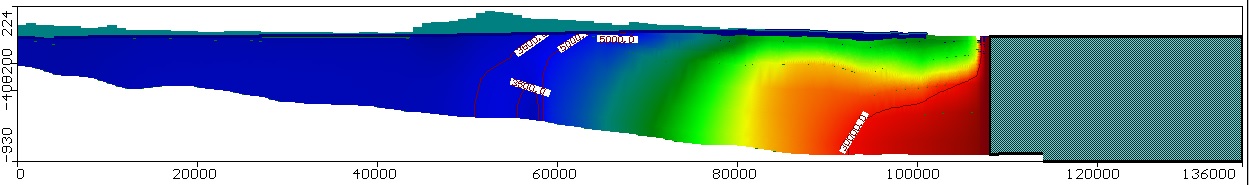 |
| 1. **1500**   **m^3^/day/well** | 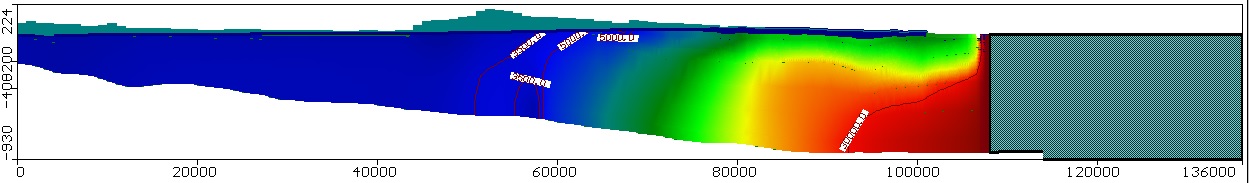 |
| 1. **1750**   **m^3^/day/well** | 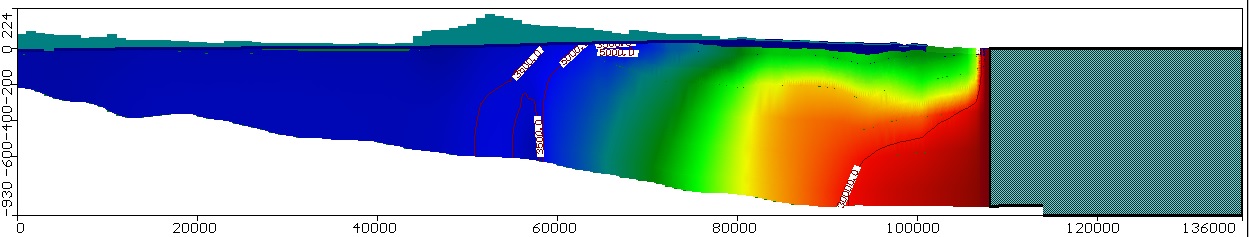 |
| 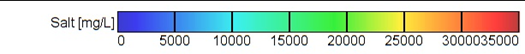 | |

**Figure A.13** Seawater distributions in vertical cross-section No.2 in the Moghra aquifer for artificial recharge controlling scenarios 13 to 16: (a) 1000 m^3^/day/well, (b) 1250 m^3^/day/well, (c) 1500 m^3^/day/well, and (d) 1750 m^3^/day/well

**Cross section No.3:**

| 1. **Base case** | 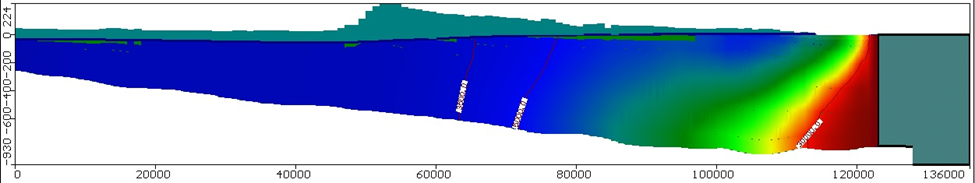 |
| --- | --- |
| 1. **1000**   **m^3^/day/well** | 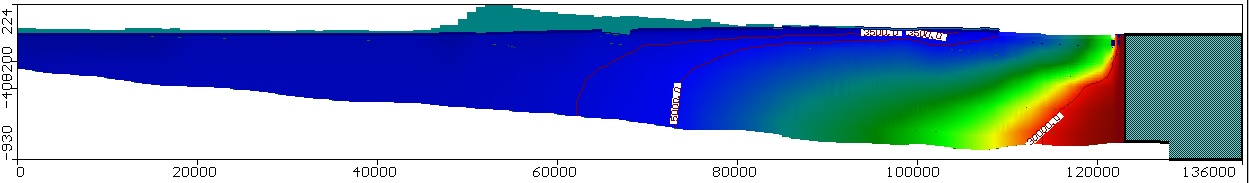 |
| 1. **1250**   **m^3^/day/well** | 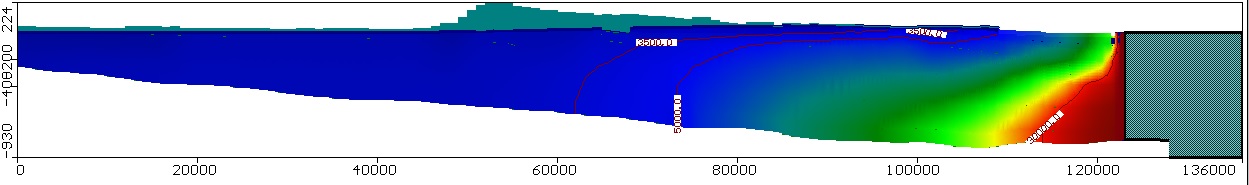 |
| 1. **1500**   **m^3^/day/well** | 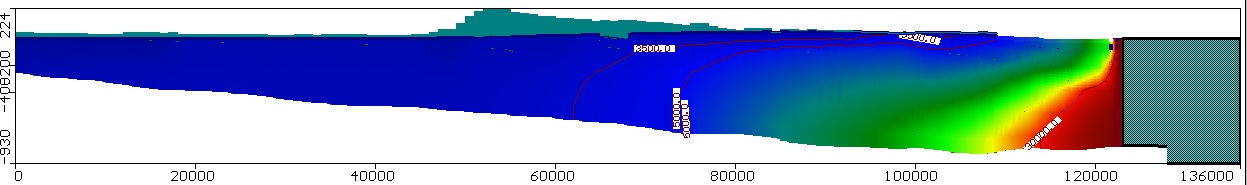 |
| 1. **1750**   **m^3^/day/well** | 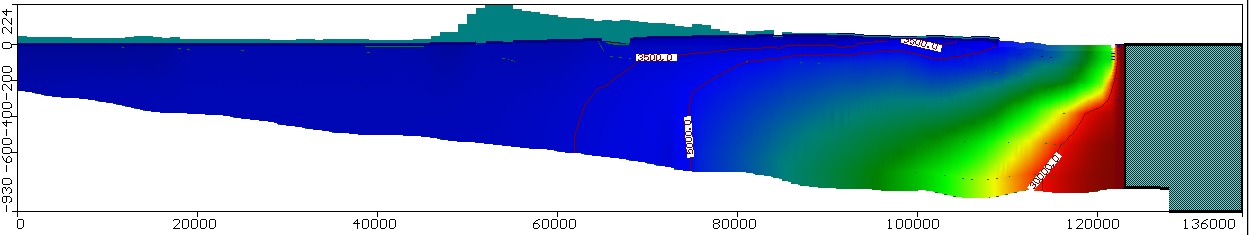 |
| 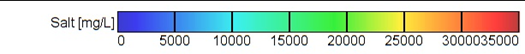 | |

**Figure A.14** Seawater distributions in vertical cross-section No.3 in the Moghra aquifer for artificial recharge controlling scenarios 13 to 16: (a) 1000 m^3^/day/well, (b) 1250 m^3^/day/well, (c) 1500 m^3^/day/well, and (d) 1750 m^3^/day/well
